# Dynamic Temporal Relationship Between Autonomic Function and Cerebrovascular Reactivity in Moderate/Severe Traumatic Brain Injury

**DOI:** 10.3389/fnetp.2022.837860

**Published:** 2022-02-16

**Authors:** Logan Froese, Alwyn Gomez, Amanjyot Singh Sainbhi, Carleen Batson, Kevin Stein, Arsalan Alizadeh, Frederick A. Zeiler

**Affiliations:** ^1^ Biomedical Engineering, Faculty of Engineering, University of Manitoba, Winnipeg, MB, Canada; ^2^ Section of Neurosurgery, Department of Surgery, Rady Faculty of Health Sciences, University of Manitoba, Winnipeg, MB, Canada; ^3^ Department of Human Anatomy and Cell Science, Rady Faculty of Health Sciences, University of Manitoba, Winnipeg, MB, Canada; ^4^ Centre on Aging, University of Manitoba, Winnipeg, MB, Canada; ^5^ Division of Anaesthesia, Department of Medicine, Addenbrooke’s Hospital, University of Cambridge, Cambridge, United Kingdom

**Keywords:** cerebrovascular reactivity (CVRx), time series analysis, traumatic brain injury, causality testing, autonomic nervous system responses

## Abstract

There has been little change in morbidity and mortality in traumatic brain injury (TBI) in the last 25 years. However, literature has emerged linking impaired cerebrovascular reactivity (a surrogate of cerebral autoregulation) with poor outcomes post-injury. Thus, cerebrovascular reactivity (derived through the pressure reactivity index; PRx) is emerging as an important continuous measure. Furthermore, recent literature indicates that autonomic dysfunction may drive impaired cerebrovascular reactivity in moderate/severe TBI. Thus, to improve our understanding of this association, we assessed the physiological relationship between PRx and the autonomic variables of heart rate variability (HRV), blood pressure variability (BPV), and baroreflex sensitivity (BRS) using time-series statistical methodologies. These methodologies include vector autoregressive integrative moving average (VARIMA) impulse response function analysis, Granger causality, and hierarchical clustering. Granger causality testing displayed inconclusive results, where PRx and the autonomic variables had varying bidirectional relationships. Evaluating the temporal profile of the impulse response function plots demonstrated that the autonomic variables of BRS, ratio of low/high frequency of HRV and very low frequency HRV all had a strong relation to PRx, indicating that the sympathetic autonomic response may be more closely linked to cerebrovascular reactivity, then other variables. Finally, BRS was consistently associated with PRx, possibly demonstrating a deeper relationship to PRx than other autonomic measures. Taken together, cerebrovascular reactivity and autonomic response are interlinked, with a bidirectional impact between cerebrovascular reactivity and circulatory autonomics. However, this work is exploratory and preliminary, with further study required to extract and confirm any underlying relationships.

## 1 Introduction

There has been little change in morbidity and mortality in moderate and severe traumatic brain injury (TBI) over the past 25 years (Carney et al., 2017; Maas et al., 2017; Donnelly et al., 2019; Steyerberg et al., 2019). TBI remains one of the leading burdens to global health (Maas et al., 2015), thus there is a need to investigate new ways to improve TBI care. Secondary injury mechanisms dictate ongoing neural injury during the acute phase of TBI care and take various forms. Such secondary injury pathways are essential targets for therapeutic intervention in moderate/severe TBI care. However, before developing precision therapeutics aimed at specific secondary injury mechanisms, we require a more comprehensive understanding of the inter-relationships between different aspects of cerebral physiology post-injury.

Impaired cerebral autoregulation in moderate/severe TBI is a secondary injury mechanism that leads to ongoing neural insult (Zeiler et al., 2020a). Literature has emerged demonstrating impaired cerebral autoregulation, assessed through cerebrovascular reactivity indices (surrogate measures for cerebrovascular autoregulation) after TBI are independently associated with mortality and poor functional outcome at 6 and 12 months post-injury (Czosnyka et al., 1997; Sorrentino et al., 2012; Zeiler et al., 2018a; Donnelly et al., 2019; Zeiler et al., 2019; Bennis et al., 2020; Zeiler et al., 2020b; Åkerlund et al., 2020). The pressure reactivity index (PRx) has emerged as one of the most commonly utilized methods for assessing cerebrovascular reactivity in the TBI literature (Zeiler et al., 2017). Despite the growing body of literature supporting the association of PRx with outcome (Czosnyka et al., 1997; Sorrentino et al., 2012; Zeiler et al., 2018a; Donnelly et al., 2019; Zeiler et al., 2019; Bennis et al., 2020; Zeiler et al., 2020b; Åkerlund et al., 2020), emerging literature suggests current guideline-based therapeutic interventions in moderate/severe TBI have little-to-no impact on the degree of impaired cerebrovascular reactivity seen (Donnelly et al., 2019; Froese et al., 2020a; Froese et al., 2020b; Zeiler et al., 2020b; Froese et al., 2020c). As such, more work is required to uncover the driving factors of impaired cerebrovascular reactivity.

Autonomic dysfunction after moderate/severe TBI has been well documented and is associated with poor global outcome (Hasen et al., 2019; Tymko et al., 2019; Fedriga et al., 2021a; Fedriga et al., 2021b). Furthermore, it is clear that autonomics and cerebrovascular function intersect (Ogoh et al., 2005; Hasen et al., 2019; Tymko et al., 2019; Fedriga et al., 2021a; Fedriga et al., 2021b). Recent literature demonstrates that PRx has an association with heart rate variability (HRV), including low frequency HRV (HRV_LF) and high frequency HRV (HRV_HF) (Lavinio et al., 2009; Sykora et al., 2016), with PRx also being connected to the baroreflex sensitivity (BRS) (Sykora et al., 2016). However, these studies had only a limited correlation between the autonomic variables and PRx, and did not examine the temporal profiles of autonomic and PRx measures. As such, a knowledge gap regarding the temporal and causal relationship between autonomic function and cerebrovascular reactivity exists.

Understanding the relationship between cerebrovascular reactivity and autonomic function is an important step to improve TBI care. The directional relationship between PRx and autonomic response portends to future targeted therapeutic development that is aimed at the prevention and reduction of secondary injury insult burden. Past work has shown that autonomic response drives factors associated with cerebrovascular reactivity, however dysautonomia has shown to be exacerbated by increases in intracranial pressure (Baguley et al., 1999; Baguley et al., 2008; Hasen et al., 2019). Beyond this, a deeper understanding of this relationship may enable the development of a more complete and accurate prognostic model that accounts for both cerebral autoregulatory and autonomic dysfunction. Ultimately, a robust understanding of how these secondary factors interconnect will improve our ability to predict patients at risk for cerebral autoregulation failure and ANS dysfunction.

Thus, using the prospectively maintained high-resolution data set from the Winnipeg Acute TBI Laboratories, we aim to examine the temporal and causal relationship between PRx and autonomic functionality in more detail using advanced time-series methodologies. The goal of this project is to comprehensively evaluate the time-series statistical properties of cerebrovascular reactivity and autonomics, focusing on the impact they have on each other. This will leverage the fact that circulatory phenomena respond in a fashion which may be assessed using the approach of linear interdependent time-series. Thus, using time-series analysis allows us to comment on what aspects of autonomic function drives cerebrovascular reactivity and gives a more complete picture of physiological response.

## 2 Methods and Materials

### 2.1 Patients

Data were accessed retrospectively from the maintained TBI database at the Winnipeg Acute TBI Laboratories, University of Manitoba. For this study, patient data were collected from June 2018 up to December 2020. All patients suffered from moderate to severe TBI (moderate = Glasgow Coma Score (GCS) 9—12, and severe = GCS of 8 or less). All patients in this cohort were admitted to the intensive care unit where they were sedated, intubated and were on volume-controlled mode of ventilation (with constant PEEP), during the course of cerebral physiologic data collection. All patients had both invasive intracranial pressure (ICP) and arterial blood pressure (ABP) monitoring conducted, per the Brain Trauma Foundation guidelines (Carney et al., 2017).

### 2.2 Ethics

Data were collected following a full approval by the University of Manitoba Health Research Ethics Board (H2017:181, H2017:188, and H2020:118).

### 2.3 Data Collection

For this study, admission demographic information was extracted following the existing prognostic models in TBI (Dijkland et al., 2021). Such demographic data collected was: age, sex, admission pupillary response (bilaterally reactive, unilaterally reactive, bilaterally unreactive) and admission GCS (both total and motor).

All patients had high-frequency digital signals recorded throughout their ICU stay. ABP was obtained through radial or femoral arterial lines connected to pressure transducers (Baxter Healthcare Corp. CardioVascular Group, Irvine, CA, or similar devices). ICP was acquired *via* an intra-parenchymal strain gauge probe (Codman ICP MicroSensor; Codman and Shurtlef Inc., Raynham, MA). All signals were captured simultaneously, synchronized and digitized via an A/D converter (DT9804; Data Translation, Marlboro, MA), sampled at a frequency of 100 Hertz (Hz) or higher using the Intensive Care Monitoring (ICM+) software (Cambridge Enterprise Ltd., Cambridge, United Kingdom, http://icmplus.neurosurg.cam.ac.uk). Signal artifacts were removed using manual methods before further processing and analysis. This ensured that all analyzed blood pressure had the distinct full wave beat, with all other data removed.

### 2.4 Signal Processing

#### 2.4.1 Cerebrovascular Reactivity

Post-acquisition processing of the above signals was conducted using ICM+, in keeping with our previously published methodology (Froese et al., 2020b; Froese et al., 2020c). First, 10-s moving averages (updated every 10 s to avoid data overlap) were calculated for all recorded signals: ICP and ABP (which produced MAP). PRx was derived *via* the moving correlation coefficient between 30 consecutive 10-s mean windows of the parent signals (ICP and MAP), updated every minute according to previously validated methods (Czosnyka et al., 1997; Sorrentino et al., 2012; Zeiler et al., 2018b; Donnelly et al., 2019; Depreitere et al., 2021).

#### 2.4.2 Autonomic Response Variables

To determine autonomic functionality, we used three categories of autonomic response that can be derived from a continuous ABP waveform. The categories were heart rate variability (HRV), blood pressure variability (BPV) and the baroreflex sensitivity (BRS). Each of these autonomic response variables (ARVs) were determined for each minute, calculated over both a 5 and 15-min window. These time windows were chosen because 5 min is a common time window for HRV (30) and a 15-min window is the minimum for a short BPV (Mena et al., 2005; Höcht, 2013; Parati et al., 2013). For spectral BPV the Lomb-Scargle periodogram was used to calculate spectral power of the ABP waveform over the 5 and 15-min window to derive the subsequent minute-by-minute updated ARV values (note these result in the power of mmHg^2^) (Sykora et al., 2016; Szabo et al., 2018). For HRV, the original ABP was processed through a peak detection algorithm based on Pan-Tomkins method (Pan and Tompkins, 1985; Sykora et al., 2016; Szabo et al., 2018). This results in an irregularly sampled peak-to-peak time series value over the 5 and 15-min window. From this the Lomb-Scargle periodogram was used over the window to calculate spectral power values for the resulting HRVs, updated every minute (note these result in the power of milliseconds^2^) (Electrophysiology, 1996; Sykora et al., 2016; Szabo et al., 2018). Thus, each variable can be time series linked to the minute-by-minute update interval of PRx and allows the implementation of times series methodologies of these spectral variables.

Due to the similarity in the final results between the time windows, all further data demonstrated will be of the 15-min windows.

HRV was derived from ABP by finding the power in 3 bandwidth categories; very low frequency (HRV_VLF; frequency less than 0.04 Hz), low frequency (HRV_LF; frequency of 0.04–0.15 Hz) and high frequency (HRV_HF; frequency of 0.15–0.4 Hz) (Electrophysiology, 1996; Berntson et al., 1997; Shaffer and Ginsberg, 2017). The interpretation of these frequencies is still up for debate (Electrophysiology, 1996; Berntson et al., 1997; Hayano and Yuda, 2019), although common interpretations are; HRV_VLF reflects slow mechanisms of sympathetic activity (though this warrants further elucidation), HRV_LF is a marker of sympathetic modulation or parameter that includes both sympathetic and vagal influences, and HRV_HF reflects parasympathetic (vagal) activity (Electrophysiology, 1996; Shaffer and Ginsberg, 2017). Due to the nature of spectral analysis of ABP waveforms the individual variables can be influenced by physiological responses that are adjunct or entirely separate from autonomic response (Electrophysiology, 1996; Berntson et al., 1997; Hayano and Yuda, 2019). Thus, any correlations must be taken as interpretations more than direct responses.

We also calculated the ratio between low and high frequency, (HRV_LF_HF; HRV_LF divided by HRV_HF) which represents minor sympathetic vagal balance or sympathetic modulations (though further investigation is still required) (Electrophysiology, 1996; Hayano and Yuda, 2019). The root mean square differences between consecutive heart beat period a heartbeat waveform (HRV_RMS) was found, which estimates the vagally mediated changes in autonomics (Electrophysiology, 1996; Hasen et al., 2019; Hayano and Yuda, 2019). The total power (HRV_TOT; which is the sum of the three spectral bands power) is a non-specific variable that reflects the overall autonomic activity (Electrophysiology, 1996; Shaffer and Ginsberg, 2017).

There were two methods of BPV found; the standard deviation of BPV in the time domain and the spectral domain analysis of BPV. The standard deviation of BPV was found in three main groups; mean blood pressure (BPV_M), systolic blood pressure (BPV_S) and diastolic blood pressure (BPV_D) over the moving time window (Höcht, 2013).

Furthermore, we assessed the spectral domain of the systolic blood pressure variability in three domain frequency ranges: low frequency (SBPV _LF; frequency of 0.077–0.15 Hz), high frequency (SBPV _HF; frequency of 0.15–0.4 Hz) and total (SBPV _TOT; total power over the full frequency range) (Höcht, 2013). Though these variables have a limited understanding, current assessments show the following: SBPV_LF variability is modulated by the sympathetic/baroreflex of vascular/vasomotor tone, total peripheral resistance and the Mayer wave (Stauss, 2007; Aletti et al., 2009; Aletti et al., 2012; Aletti et al., 2013) and SBPV_HF variability is mainly influenced by changes in cardiac output, parasympathetic and respiration action (Janssen et al., 1995; Aletti et al., 2009; Aletti et al., 2013).

Finally, the baroreflex sensitivity (BRS) was calculated using a modification of the sequential cross-correlation method. ABP was used to find systolic peaks and the heart beat period, then a linear regression between the 10-s series of heart beat period and the corresponding 10-s series of systolic blood pressure over the time window results in the BRS (Westerhof et al., 2004). BRS may provide a useful synthetic index of neural regulation at the sinus atrial node, though limitations still exist with this interpretation (Kardos et al., 2001; La Rovere et al., 2008; Pinna et al., 2015).

### 2.5 Time-Series Analyses

We implemented a wide variety of time-series tests and models to assess both functionality and associated causality between the individual ARVs and PRx. To help elucidate the uses of these methods as well as the limitations and pitfalls of such techniques, we will give an overview of the methods. However, a full conceptual understanding of these individual methodologies can be found from their respective literature (McQuitty, 1966; Granger, 1969; Lütkepohl, 2005; Rokach et al., 2005; Murtagh and Legendre, 2014; Kilian and Lütkepohl, 2017; Chatfield and Xing, 2019; Thelin et al., 2019).

The three major methods used were: vector autoregressive integrated moving average (VARIMA) impulse response function plots (IRF), Granger Causality Testing and Hierarchical Clustering. These methods were chosen for their exploratory nature and their use in previous analyses of temporal physiology within TBI (Zeiler et al., 2018c; Zeiler et al., 2018d; Thelin et al., 2019).

#### 2.5.1 Vector Autoregressive Integrative Moving Average Impulse Response Functions Analysis

IRF are used to graphically demonstrate the causal effect of an impulse on a system. For our uses we created VARIMA models to represent the relationship between PRx and each ARV. Then we created a simulated impulse on the VARIMA model from each respective ARV on PRx and vice versa. In this way we graphically demonstrated the patient specific interaction between each ARV and PRx.

##### 2.5.1.1 Autoregressive Integrative Moving Average Structure Analysis

In order to derive a VARIMA model in an effective manner (due to the heavy computational requirements of such a method) but also to evaluate the accuracy of a VARIMA model, we performed a Box-Jenkin’s autoregressive integrative moving average (ARIMA) model for each patients’ PRx and all ARVs (Lütkepohl, 2005; Zeiler et al., 2018c; Chatfield and Xing, 2019).

Initially, PRx and ARVs were evaluated for time stationarity using the: autocorrelation function (ACF) plots, partial autocorrelation function (PACF) plots and Kwiatkowski–Phillips–Schmidt–Shin (KPSS). The augmented Dickey-Fuller (ADF) was used to test for root trend. Note we assume that all variables have some aspect that is both interdependent and linear, due to the interconnection of circulatory/vascular function.

Next, the optimal ARIMA structure for PRx and ARVs were derived for each patient. Initially, the *auto.arima* (a pre-built R function) was used to determine the upper order limit for tested ARIMA models (auto.arima, 2021). Based on this, autoregressive order (p) was varied from 1 to 10, and the moving average order (q) was varied from 0 to 10, while the integrative order (d) was held at 0. The integrative order was held at 0 as the tests of ACF, PACF, KPSS, and ADF suggested that all the signals were stationary. This is in keeping with previous time-series work in TBI literature (Zeiler et al., 2018c; Zeiler et al., 2018d; Thelin et al., 2019). All the permutations of the ARIMA orders were assessed using the Akaike Information Criterion (AIC), and Log-Likelihood (LL) recorded for every model.

Using the AIC and LL, the optimal ARIMA structures for PRx and ARVs were compared in the datasheets, with the lowest AIC and highest LL values indicating superior models. A general Box-Jenkin’s ARMA model for PRx can be found in Supplementary Appendix SA1.

**FIGURE 1:**
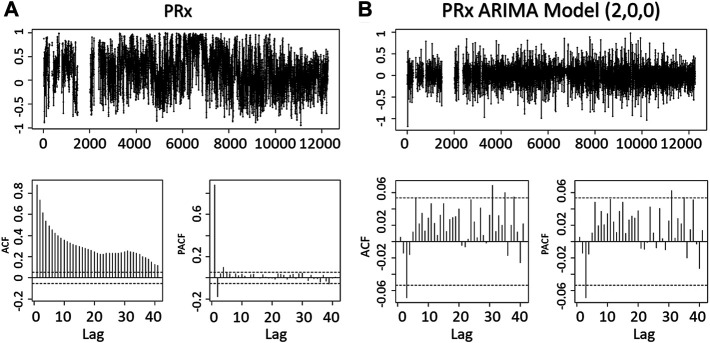
The Residuals, ACF, PACF of the PRx and PRx ARIMA Model. The figures demonstrate the minute-by-minute PRx data and an optimal ARIMA model (autoregressive order of 2, integrative order of 0 and moving average order of 0) obtained from the Akaike Information Criteria and Log Likelihood. The reduced significant lags in ACF or PACF of the ARIMA model shows that the ARIMA model in part accounts for the residual relationship between the PRx data. ACF, autocorrelation function; PACF, partial autocorrelation function; PRx, pressure reactivity index; ARIMA, autoregressive integrated moving average.

A patient example of the serial ARIMA model testing with AIC and LL outputs can be found in Supplementary Appendix SB in Supplementary Material. Similarly, an example in Figure 1 is given of the raw signal ACF and PACF plots, followed by the plots for the residuals of the optimal ARIMA model (found through LL and AIC), indicating that the autocorrelative structure has been adequately accounted for.

##### 2.5.1.2 Vector Autoregressive Integrative Moving Average (VARIMA) Models

Next, we derived multi-variate VARIMA models to evaluate the impulse response of ARVs on PRx and vice versa. These models explore the behavior of two-time series variables, recorded simultaneously, and are derived through the extension of the standard Box-Jenkin’s models into multi-variate systems (further descriptions can be found in the references) (Lütkepohl, 2005; Kilian and Lütkepohl, 2017; Chatfield and Xing, 2019). A formula representing the vector autoregressive moving average model (VARMA) of PRx and an ARV can be found in Supplementary Appendix SA2, which is a VARIMA with the integrative order held at 0.

Since the ACF and PACF did not indicate any cyclical trends in the variables, with ADF and KPSS indicating that all variables were stationary, we employed basic VARMA models with autoregressive order of four and moving average order of four. This was based on the findings from individual patient ARIMA models of ARVs for each patient and past work evaluating the ARIMA models of ICP and MAP (Thelin et al., 2019; Zeiler et al., 2020c; Zeiler et al., 2020d).

A VARIMA model autoregressive order of four was chosen given the optimal ARIMA models for many ARVs was less than two, as well as previous studies found that most patient’s ICP and MAP had an autoregressive order of two (Thelin et al., 2019; Zeiler et al., 2020c; Zeiler et al., 2020d). Thus, as suggested by Helmut Lutkepohl, taking the product of the ARIMA autoregressive orders for VARIMA modelling is a method to ensure adequate model structure (Lütkepohl, 2005), and thus the order of four. For the moving average order for the VARIMA model, a value of 4 was chosen based on the previous study of ICP and MAP (52,59,60) and ARVs optimal order being below two in many patients. Thus, the sum of the two ARIMA moving average orders was used to get a final VARIMA moving average order of 4.

##### 2.5.1.3 Impulse Response Function Analysis

Next, the coefficients derived from these VARIMA models were employed to derive the IRF plots between PRx and an individual ARV. The IRF plots provide a descriptive graphical representation of the impact of PRx on an ARV, and an ARV on PRx, by using the previously generated VARIMA models and modelling a one unit orthogonal impulse of one variable on the other, and vice versa (Kilian and Lütkepohl, 2017). Depicted in the plot is how much one variable will fluctuate as a response to the impulse from the other variable. A bootstrapping method was used to derive the confidence interval of the population using sampled data. Bootstrapping involves using only part of the sample data for a run, then comparing all the runs, in this design a standard percentile bootstrap interval of 100 runs was used (Efron and Tibshirani, 1993).

Due to the high variability in the IRF plots and the difficulty that arises with simple graphical interpretations of large datasets, a simple method to help separate models into two categories (“more responsive” verses “less responsive”) was used. To do this the impulse response was normalized with respect to its original variable data. Then identified if the response was greater than an absolute value of 0.001 threshold (chosen as at least a 0.1% change in the normalized response) after 10 min (one complete cycle of PRx after the initial impulse, i.e., 5 min post PRx calculation window). In this way, we could infer if the impulse created a stronger response within the subsequent variables and differentiate responses.

#### 2.5.2 Granger Causality Testing

Granger causality testing is used to identify the assistance of one interdependent variable to predict another interdependent variable, beyond the degree to which the variable predicts itself (Barnett et al., 2009). In this case, the ability for PRx to predict an ARV (beyond the ability for the ARV to predict itself) and vice versa. Thus, with the minute-by-minute time series linked ARV data to PRx we could perform a Granger causality test between these interdependent variables.

For the Granger causality test, we recorded the response for every patient, both F-test statistic value and *p*-values for all ARVs vs. PRx (Granger, 1969). The Granger causality responses were assessed to identify the reciprocal influences between PRx and ARVs.

#### 2.5.3 Co-Variance Cluster Analysis

Finally, to confirm our findings regarding the relationship between ARVs and PRx, a hierarchical clustering method was used on each patient to identify which ARVs and PRx was most closely associated. Using a divisive method, we separated the variables using the Euclidean distance of the normalized variables and the *hclust* (a prebuilt R function) (McQuitty, 1966; Rokach et al., 2005; Murtagh and Legendre, 2014).

#### 2.5.4 Sub-Group Analysis

The entire database was subdivided based on some simple parameters and re-evaluated to see if the VARIMA IRF analysis or Granger causality test displayed any outlying groups. Parameters included were age (<60 vs. age ≥60; moderate vs old age), Glasgow outcome scale extended (GOSE) at 6 months (<2 vs. ≥ 2; dead vs alive), Marshall computer tomography (CT) score (<4 vs. ≥ 4; mass lesion vs diffuse injury), sex (male vs. female), the first 24 h only and the first 72 h only. Due to the similarity between the first 24 h only, 72 h only and the full time; all data presented will be of the full time.

### 2.6 Statistics

All statistical analyses were conducted using R (R Core Team (2016). R: A language and environment for statistical computing. R Foundation for Statistical Computing, Vienna, Austria. URL https://www.R-project.org/). The alpha was set at 0.05 for significance. No multiple error correction test was performed at this time as the analysis is in the preliminary exploratory phase. The patient population was summarized using simple descriptive statistics, including median/mean and standard deviation/IQR where applicable.

For the Granger causality test, we performed a Mann-Whitney U comparison test between the F-statistics of PRx on an ARV and an ARV on PRx over the entire population. As well we bar plotted the number of significant *p*-values for each variable. This gives the overarching relationship between the PRx and each ARV.

## 3 Results

### 3.1 Patient Demographics

A total of 47 patients were included in this study. The mean age was 43.5 ± 23.5 years, with 37 (80.9%) being males. The median admission total GCS score was 6 (IQR: 3.5–9), and motor sub-scores were 4 (IQR: 1.5–5). Six patients (12.8%) presented with bilaterally unreactive pupils, and ten (21.3%) unilaterally unreactive pupils. The median duration of digital signal recording was 2.67 (IQR: 1.35–5.74) days. Table 1 displays the patient admission demographics and injury information. Supplementary Appendix SC shows the median value for each measured variable over the full data set.

**TABLE 1:** 47 Patient demographics.

Demographics	Mean (Interquartile range)
Age	38 (28.5–51)
Sex (% Male)	80.9%
Best admission GCS—total	6 (3.5–9)
Best admission GCS—motor	4 (1.5–5)
Number with hypoxia episode	20
Number with hypotension episode	5
Number with traumatic SAH	45
Number with epidural hematoma	5
Pupils	—
Bilateral unreactive	6
Unilateral unreactive	10
Bilateral reactive	31
Admission marshall CT	—
V	12
IV	8
III	14
II	3
1 month GOSE	6 (4.5–6)
30-Day mortality	27.7%
Average ICU stay (days)	6.34 (5.32–8.25)

CT, computerized tomography; GOSE, extended Glasgow outcome scale; GCS, Glasgow comma score; ICU, intensive care unit; SAH, subarachnoid hemorrhage.

### 3.2 VARIMA and Impulse Response

To assess the relationship between PRx and ARVs, we employed VARIMA modelling with an IRF. VARIMA models of autoregressive order 4 and moving average order 4 were employed for each patient for the first 24 h only, 72 h only and the full data. IRF plots provide a descriptive visualization of the relationship between each ARV and PRx (examples seen in Supplementary Appendix SD). These IRF plots allowed us to visually determine the temporal relationship between PRx and ARVs, assessing the impact of one unit impulse on the respective variable.

Overall, there was high variability in absolute changes in the PRx and ARVs. However, ARVs demonstrated a higher magnitude in impulse response in PRx than the alternative. This was explored through the number of patients that exceeded an absolute value threshold of 0.001, with PRx responses being greater in amplitude for an ARV orthogonal impulse than the converse.

BRS demonstrated the most consistent number of patients in the “more responsive” cohort, with BRS impulse on PRx resulting in 9 “more responsive” patients vs PRx impulse on BRS resulting in 7 “more responsive” patients. Other ARVs on PRx that had at least 10% of the population (over five patients) in the “more responsive” category were; HRV_VLF, SBPV_HF, SBPV_TOT, BPV_M, and BPV_D. Likewise for PRx on APVs demonstrating over five patients in the “more responsive” category only had BRS and HRV_VLF.

### 3.3 Granger Causality

**FIGURE 2:**
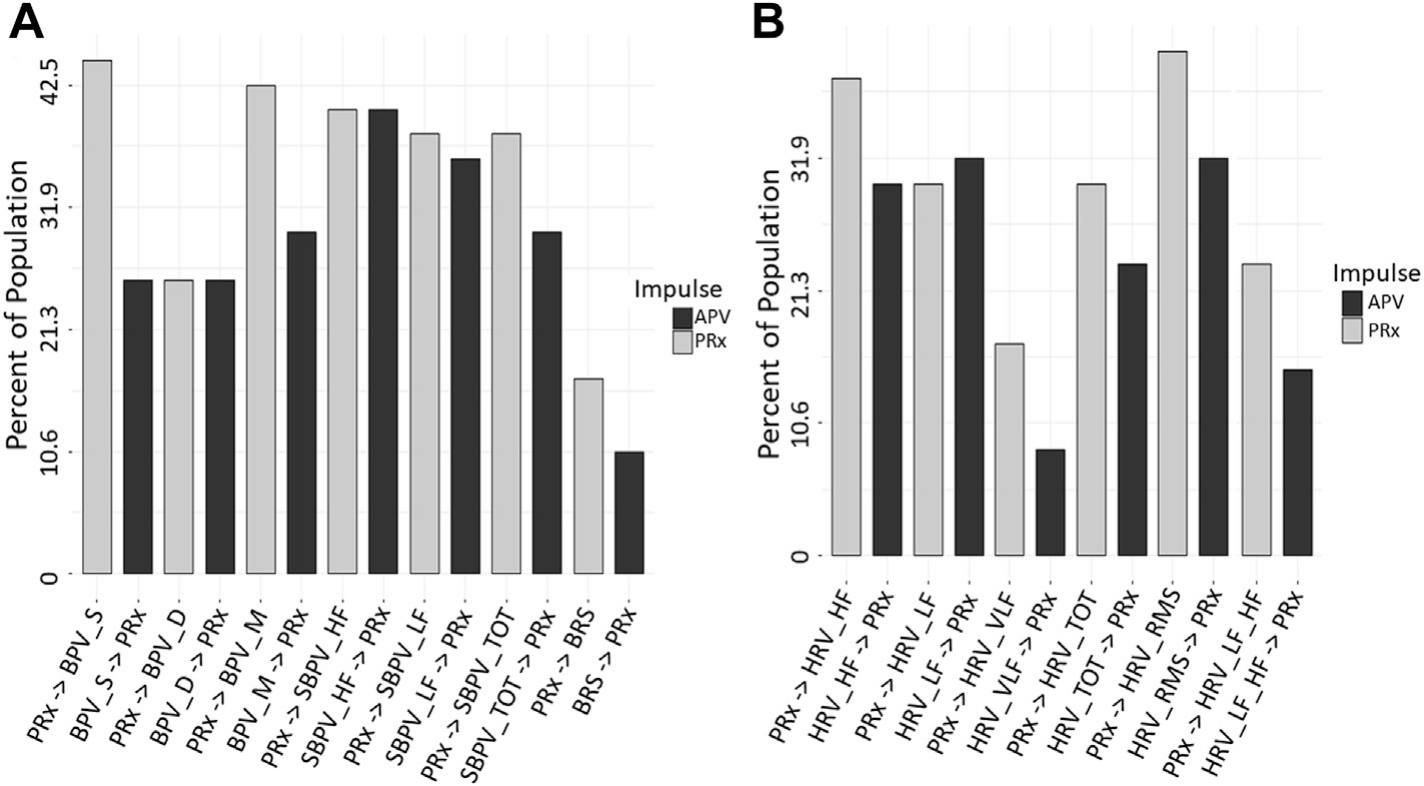
Granger Causality *p*-value Comparisons (*n* = 47). The bar graphs show the number of significant Granger Causality *p*-values of PRx on an ARV and an ARV on PRx, in this way the impulse that has more significant responses may be considered to have a greater influence on causality. Image **(A)** shows all BPV and Baroreflex, **(B)** shows all HRV. ARV, autonomic response variable; BPV_D, standard deviation of diastolic blood pressure variability; BPV_M, standard deviation of mean blood pressure variability; BPV_S, standard deviation of systolic blood pressure variability; HRV, heart rate variability; HRF_HF, heart rate variability high frequency; HRV_LF, heart rate variability low frequency; HRV_LF_HF, heart rate variability ratio between low/high frequency; HRV_RMS, heart rate variability root mean square; HRV_TOT, heart rate variability total; HRV_VLF, heart rate variability very low frequency; PRx, pressure reactivity; SBPV_HF, spectral blood pressure variability high frequency; SBPV_LF, spectral blood pressure variability low frequency; SBPV_TOT, spectral blood pressure variability total.

To assess the directional response between ARVs and PRx, we performed a Granger causality test comparing data sets for all patients. The Mann-Whitney U comparisons test between PRx and ARVs are in Supplementary Appendix SE, with Figure 2 showing the number of patients with significant *p*-values (demonstrating a causal connection). In general, across the population, we found that the Granger causality test was inconclusive, with bidirectional causal features between PRx and ARVs seen across the cohort. In addition, some patients favored PRx impacting ARVs, though some showed the alternative causal relationship and not all directional relationships reached significance. Supplementary Appendix SF provides the Granger test results, including F-test and *p*-values, for every patient using the entire data set of full time. Of note, the causal direction of the relationship was not significantly changed when evaluating only the first 24 or 72 h of data.

The only variable that had a significant *p*-value was HRV_RMS in the first 24 and 72 h only. From Figure 2 variables of HRV_VLF, HRV_LF_HF, BPV_S and BRS all had a moderate reduction in *p*-value significant patients from PRx on APV to APV on PRx. This may indicate a more impactful response from PRx in these relationships.

From the VARIMA IRF, PRx impulse most often caused a decrease in BRS. All other variables failed to have a consistent common PRx impulse response.

### 3.4 Hierarchical Cluster

**FIGURE 3:**
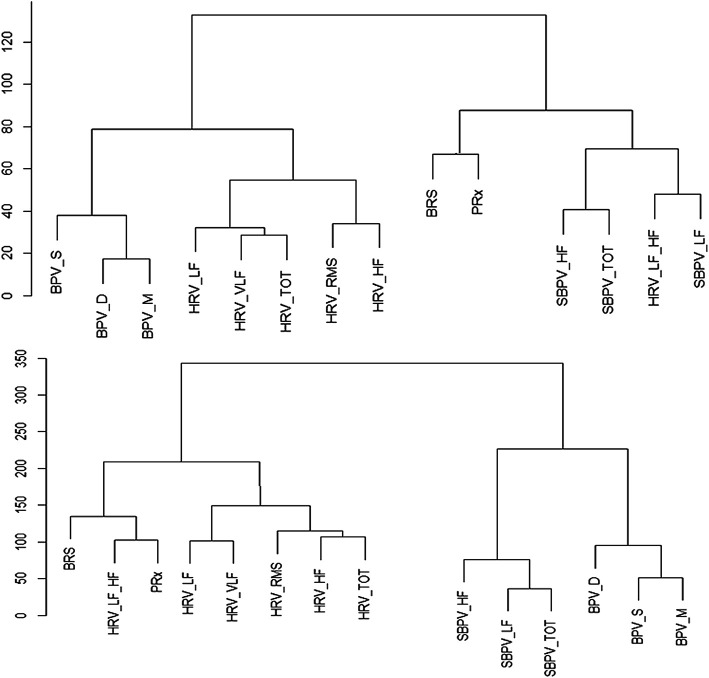
Example of Hierarchical Clusters of Two Patients. Hierarchy cluster example from two patients, the data is normalized, and the distance between the variables is Euclidean. The distance between two variables shows the height of the dendrogram where the two branches merge into a single branch, thus two variables that diverge on the last branch may be more closely linked. BPV_D, standard deviation of diastolic blood pressure variability; BPV_M, standard deviation of mean blood pressure variability; BPV_S, standard deviation of systolic blood pressure variability; HRF_HF, heart rate variability high frequency; HRV_LF, heart rate variability low frequency; HRV_LF_HF, heart rate variability ratio between low/high frequency; HRV_RMS, heart rate variability root mean square; HRV_TOT, heart rate variability total; HRV_VLF, heart rate variability very low frequency; PRx, pressure reactivity; SBPV_HF, spectral blood pressure variability high frequency; SBPV_LF, spectral blood pressure variability low frequency; SBPV_TOT, spectral blood pressure variability total.

Finally, the hierarchical clustering analysis helped confirm the connection that the variables had with one another. Though there was significant heterogeneity in the individual plots, PRx was most associated with BRS and HRV_LF_HF in a majority of patients. For an example plot, Figure 3. With the full data cophenetic correlation and dendrogram displayed in Supplementary Appendix SG.

### 3.5 Age, GOSE and Significant Patients

Sub-group analysis failed to demonstrate any significant findings or finding that would be consider different from the full data. Dividing patients into sex, <60 vs. ≥60 age groups and six-month GOSE of <2 vs. ≥2 did not identify any significant outliers or an increased percentage of responsive patients. However, nearly all VARIMA IRF plots with a significant response (either PRx on an ARV or vice versa) had a Marshall CT score of 4 or higher.

## 4 Discussion

We compared cerebrovascular reactivity as measured through PRx with various ARVs like HRV, BPV and BRS. We evaluated underlying behaviors by employing complex time-series analyses, like: ARIMA, VARIMA, IRF plots, Granger causality testing, and hierarchical clustering. Through this evaluation of PRx and ARVs, some exploratory insights into the association in physiological responses were illustrated. The preliminary evidence supports the idea that certain ARVs may have a clinically relevant association with impaired PRx. Further, results here help outline the framework for the role that systemic autonomic response has on cerebral autoregulation. However, the insights here must be tempered by the following limitations inherent with the use of any continuous ABP waveform method but in particular those of a spectral nature.

Foremost is the fact that significant changes to HRV, BPV or BRS may have occurred due to concomitant incidents outside of physiological autonomic response. In order to limit this, we followed the outlined method to achieve an optimal HRV, which are; signal capturing should allow signal reconstruction without amplitude and phase distortion, individual subjects should be recorded under fairly similar conditions and environments, and complete signals should be carefully edited using visual checks (Electrophysiology, 1996). Patients were all in the ICU with TBI, and were sedated, intubated and were on volume control mode of ventilation, with constant PEEP, during the course of cerebral physiologic data collection. The sympathetic nervous system seems to attenuate the CO_2_-induced increases in CBF (Jordan et al., 2000), which can be mediated by ventilation in the ICU. Though it has been noted that powerful actions of mechanical ventilation induces periodical modifications of the intrathoracic pressure, modulating venous return which has shown to alter cardiovascular and cerebrovascular interactions (Innes et al., 1993; Elstad et al., 2011; Porta et al., 2021). With literature assessing the relationship between PEEP and cerebral reactivity in pigs, demonstrating that static PEEP improved the assessment of impaired/intact cerebrovascular reactivity (Brady et al., 2012; Fraser et al., 2013). Furthermore, the ABP changes slower then 30 s were linked to PRx, with improved consistency, when PEEP was constant (Fraser et al., 2013). Taken together, this highlights the need for more study between respiratory control and vascular influences. Finally, when the rate of change in blood pressure or cardiac output is rapidly altered, cerebral autoregulation has a reduced ability to regulate CBF (Levine et al., 1994; Zhang et al., 2002; Ogoh et al., 2005; Ogoh et al., 2007). Though it is impossible to limit all situations of extreme systemic circulatory response in the ICU, as part of critical TBI targets, ICP and cerebral prefusion pressure are kept in moderate ranges (Carney et al., 2017). With this in mind the results can be summarized as the following.

First PRx, appears to causally impact ARVs as displayed through Granger causality, with the IRF plots of ARVs on PRx demonstrating a greater change then the inverse. This implies that derangements in cerebrovascular reactivity may, in turn, cause fluctuations in systemic circulatory autonomic responses. However, based on IRF results, any fluctuations in systemic circulatory autonomic responses result in larger derangements to cerebrovascular reactivity than the inverse. In a past study that assessed MAP and CBF, there is a slight bidirectional interaction between MAP and CBF but MAP mostly had a unidirectional impact on CBF (assessed over ∼2 min windows) (Schiatti et al., 2014). Further in patients either in a normal or tilt table posture, there was association between MAP and CBF (Bari et al., 2017). Thus, underlying patient characteristics could influence the directionality of the relationship between cerebrovascular reactivity and autonomic function.

Second, certain ARVs like BRS, HRV_LF_HF and HRV_VLF demonstrated a directional impact on PRx as assessed through Granger causality and a strong impulse response in PRx, in over 10% of patients (*n* > 5). This implies that autonomics may, in fact, be responsible for causing derangements in cerebrovascular reactivity and may be important in achieving optimal CBF. Furthermore, from the associated nature of BRS, HRV_VLF and HRV_LF_HF, the sympathetic autonomic efferent response may be the primary autonomic factor associated with PRx response (Lindvall et al., 1978; Karemaker, 2017). This however is still up for debate as previously stated the direct interpretation of ARV response is limited, with the ARV being most commonly associated with sympathetic response (HRV_LF) remaining similarly connected to PRx as other ARVs. This may be due to the limited number of patients and requires further evaluation.

Despite this, previous literature has linked PRx to HRV_LF (Sykora et al., 2016), with other studies that demonstrated HRV_LF and PRx independently correlated with outcome (Lavinio et al., 2009; Gao et al., 2016). Moreover, sympathetic nerves modulating resistance vessels tone has been demonstrated (Baumbach and Heistad, 1983). As well the sympathetic tone during spinal cord stimulation confirmed indirectly its role in mediating the CBF (Visocchi, 2006; Visocchi, 2008). Likewise CBF autoregulation is shifted towards higher blood pressure levels during sympathetic activation (Bill and Linder, 1976; Sadoshima et al., 1985). Furthermore, two independent studies suggested that stimulation of the sympathetic nerves can extend the limit of autoregulation (Fitch et al., 1975; MacKenzie et al., 1979). Thus, derangements of sympathetic autonomics may, in turn, interfere with cerebral vascular reactivity in certain patients.

However, previous work has demonstrated that HRV_HF can predict impaired cerebrovascular reactivity (PRx > 0.2) (Lavinio et al., 2009). Likewise, a study performed by Fedriga et al. indicated that CBF is maintained by the baroreflex and the parasympathetic autonomic response (as assessed by the association of BRS and HRV_HF with the upper limits of ICP values) during plateau waves of ICP (Fedriga et al., 2021a). In conjunction with this, HRV_HF and BRS in the past have shown a connection with ICP and cerebral perfusion pressure (Goldstein et al., 1998; Ogoh et al., 2007; Kox et al., 2012; Hasen et al., 2019; Fedriga et al., 2021a). However, the only situations where HRV_HF (representing the parasympathetic autonomic response) and PRx are connected is when cerebrovascular reactivity is already impaired, for example, during extremes of ICP elevation. In such states where ICP is at extreme levels or cerebrovascular reactivity is heavily impaired, the natural homeostasis of cerebral autoregulation is already heavily deranged, and thus any subsequent variation in systemic blood pressure (especially those of a higher frequency nature) would be reciprocated in the ICP response. This may account for why previous studies have linked the parasympathetic response to cerebrovascular reactivity and encourages the idea that the derangement of PRx is linked primarily to the sympathetic response of the autonomic system. However, we must acknowledge that the results found in this manuscript are preliminary and require much further validation.

Finally, nearly all VARIMA IRF plots with a significant response (either PRx on an ARV or vice versa) had a Marshall CT score of 4 or higher. This may indicate intracranial injury burden as a driver of the autonomic/cerebrovascular reactivity relationships, which is in keeping with recent literature supporting the strong association between diffuse acceleration-deceleration injury patterns and the development of cerebral autoregulation impairment/failure (Hiler et al., 2006; Zeiler et al., 2018e; Zeiler et al., 2020e).

In summation, there appears to be a directional impact of PRx on ARVs as assessed through Granger causality and IRF plots in some patients. Despite corroboration through various statistical approaches, the outcome of this study should be interpreted as only a preliminary exploration into the interconnected nature between ARVs and cerebrovascular reactivity. The responses themselves are significantly heterogeneous from patient to patient, with the IRF showing both positive and negative responses in PRx values. Thus, future work continuously analyzing PRx and ARVs would benefit from large cohorts separated into key demographical groups, with the use of clustering methodology to isolate homogeneous physiological factors. Key among these groups would be patients with impaired vs intact cerebrovascular reactivity. The heterogeneity in patient response, coupled with the small cohort size, leaves these statistical models quite limited in their overall assessment.

## 5 Limitations

As this was an exploratory analysis of the ARVs and PRx, many overarching limitations could be assessed. First, this is a retrospective analysis of a relatively small prospectively collected dataset. As such, our findings should only be considered exploratory and preliminary. Further, the results here may not be generalizable to other TBI populations and requires validation in larger multi-center high-frequency physiologic datasets. Second, patient injury severity and treatment heterogeneity could have influenced the physiologic signal response and is something that will require more tailored and refined datasets, with the use of clustering methodology to isolate homogeneous physiological factors. Third, the nature of these ARVs and their connection with cerebral autoregulation is severely limited, with the individual variables themselves still up to interpretation as to which aspect of the autonomic nervous system they truly represent (Hayano and Yuda, 2019). Thus, avenues that focus on the more extreme cases of autonomic and PRx change may provide more useful insights as to the effect of such variable responses, as demonstrated in the limited previous literature on plateau waves in moderate/severe TBI cohorts (Hasen et al., 2019; Tymko et al., 2019; Fedriga et al., 2021a; Fedriga et al., 2021b). Likewise there is a known influence of mechanical ventilation on ARV, and thus the evaluation of patient populations outside of intensive care may allow for more conclusive results.

Finally, the statistical methodology employed was computationally tasking and, as such, implementing such methodologies on larger cohorts would benefit from more robust central computing services.

## 6 Conclusion

Using statistical methods like ARIMA, VARIMA IRF, Granger causality and hierarchical clustering, we evaluated the temporal relationship between ARVs and cerebrovascular reactivity (as measured through PRx) in moderate/severe TBI patients. Granger causality testing demonstrated inconclusive results, with bidirectional relationships between PRx and ARVs in most of the cohort studied. However, the ARVs of BRS, HRV_LF_HF and HRV_VLF all demonstrated a stronger connection to PRx than other ARVs, indicating that the sympathetic autonomic response may be connected to cerebrovascular reactivity derangements. Finally, BRS was consistently one of the most responsive ARVs to PRx, possibly demonstrating a unique connection. However, this work is exploratory and preliminary, with further examination required to extract any underlying relationships.

## Data Availability

The original contributions presented in the study are included in the article/Supplementary Material, further inquiries can be directed to the corresponding author.
