# Supplementary material for: Dynamic Temporal Relationship Between Autonomic Function and Cerebrovascular Reactivity in Moderate/Severe Traumatic Brain Injury
Source: Front Netw Physiol. 2022 Feb 16;2:837860. doi: 10.3389/fnetp.2022.837860 (PMC10013014; doi:10.3389/fnetp.2022.837860)
Supplement: Supplementary file 1 [file DataSheet4.DOCX]

Appendix C. VARIMA IRF Plots for Patients

*The following is all the IRF plots for some patients, demonstrating an ARV on PRx then PRx on an ARV. The confidence interval is derived using a bootstrap method of 100 models taking the 95% confidence interval from them. ARV, autonomic response variable; BPV_D, standard deviation of diastolic blood pressure variability; BPV_M, standard deviation of mean blood pressure variability; BPV_S, standard deviation of systolic blood pressure variability; HRV, heart rate variability; HRF_HF, heart rate variability high frequency; HRV_HF_LF, heart rate variability ratio between high/low frequency; HRV_LF, heart rate variability low frequency; HRV_LF_HF, heart rate variability ratio between low/high frequency; HRV_RMS, heart rate variability root mean square; HRV_TOT, heart rate variability total; HRV_VLF, heart rate variability very low frequency; IRF, impulse response function; PRx, pressure reactivity; SBPV_HF, spectral blood pressure variability high frequency; SBPV_LF, spectral blood pressure variability low frequency; SBPV_TOT, spectral blood pressure variability total; VARIMA, vector autoregressive integrated moving average;*

**Patient 1 – APV on PRx**

**
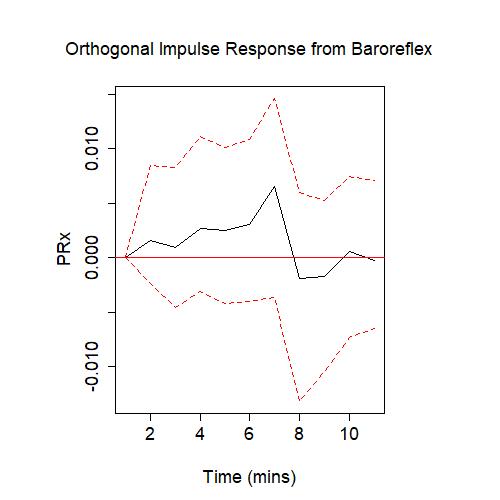

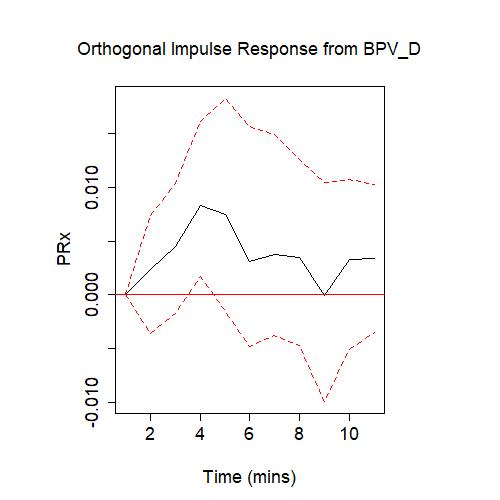

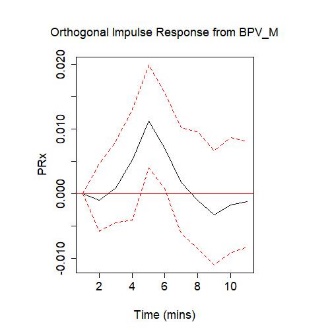

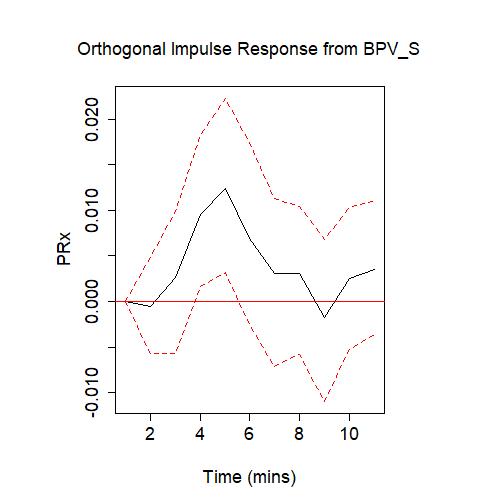

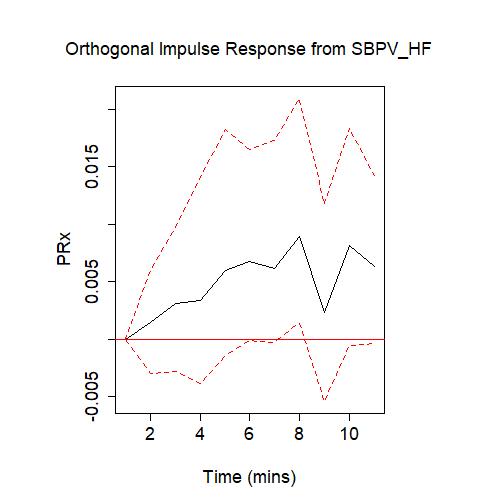

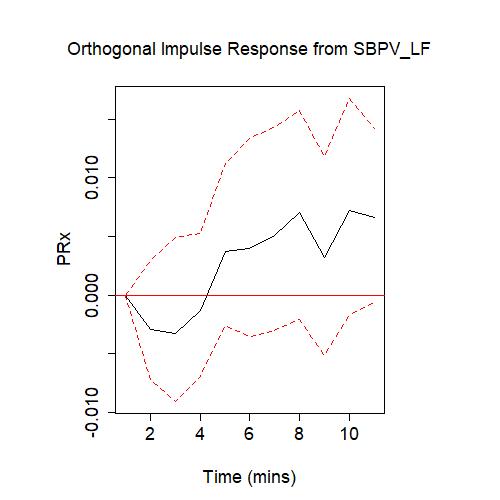

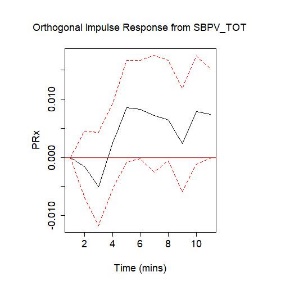

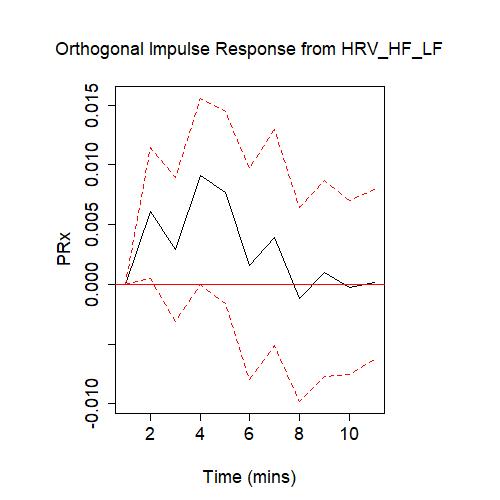

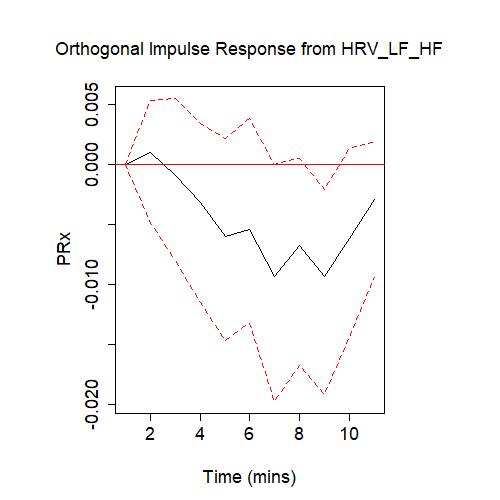

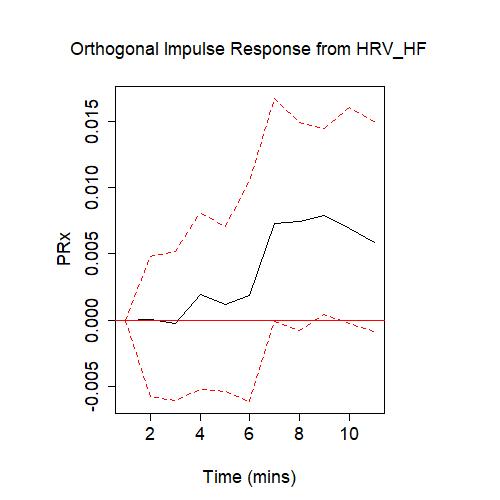

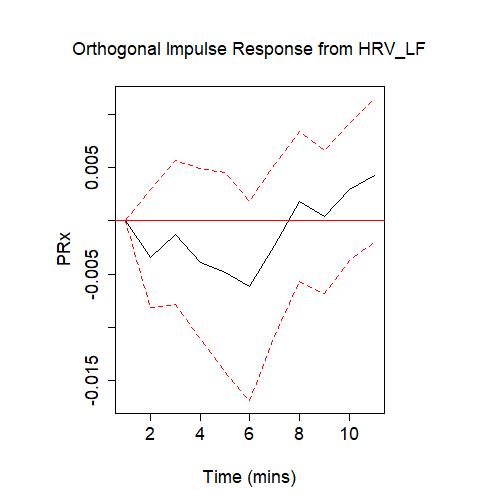

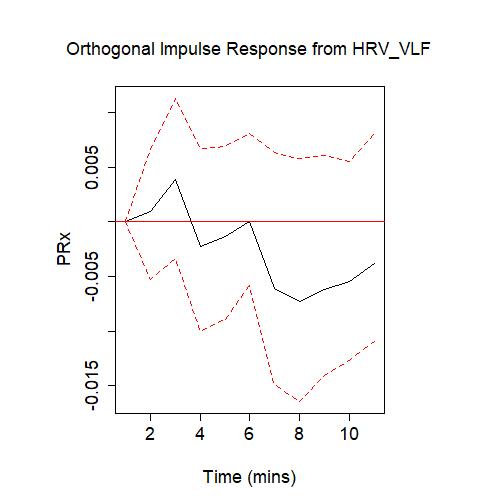

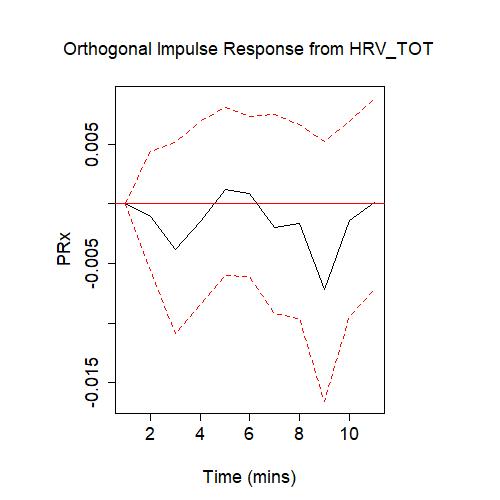

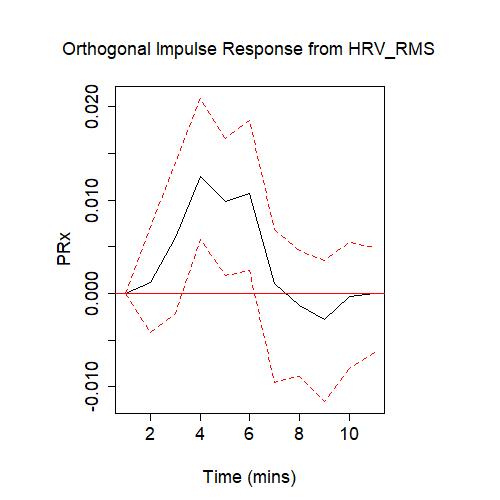
**

**Patient 1 – PRx on APV**

**
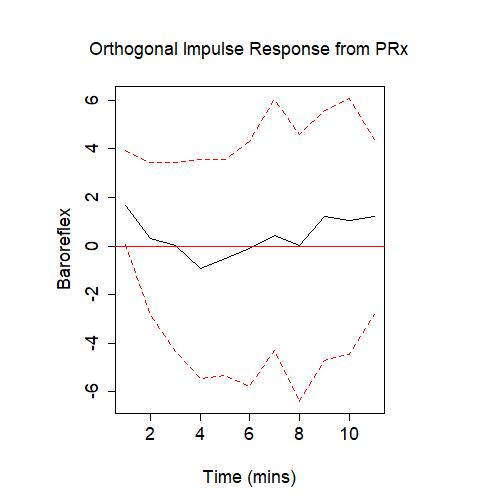

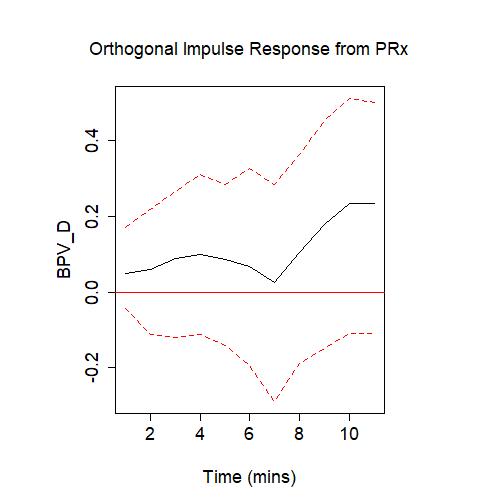

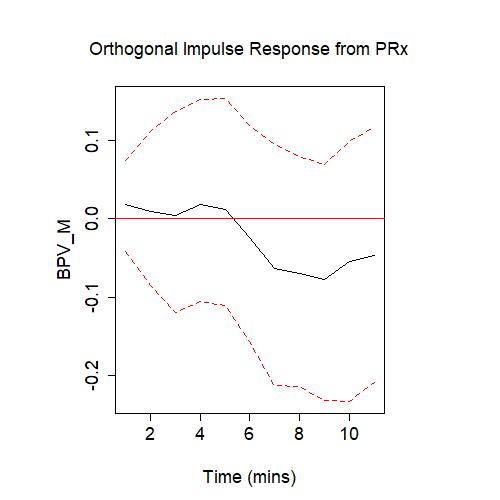

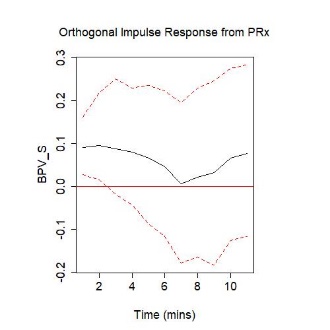

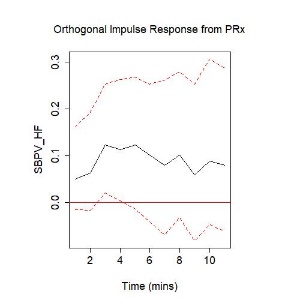

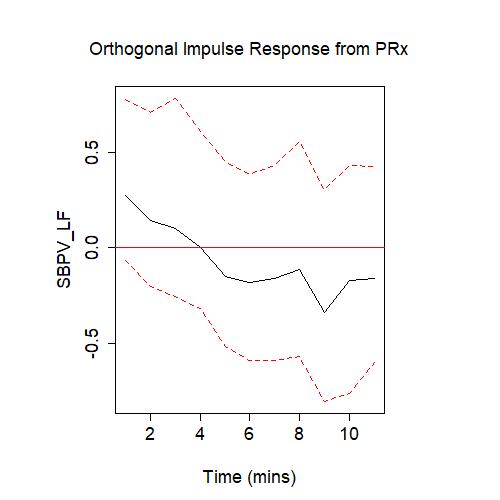

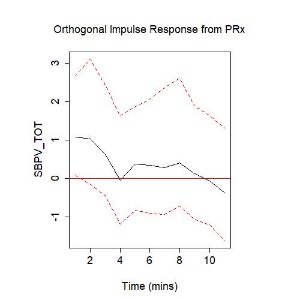

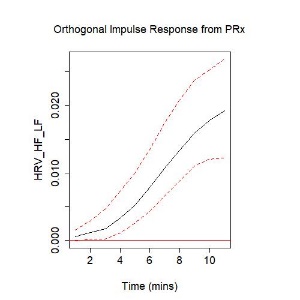

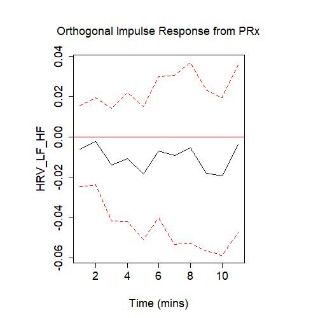

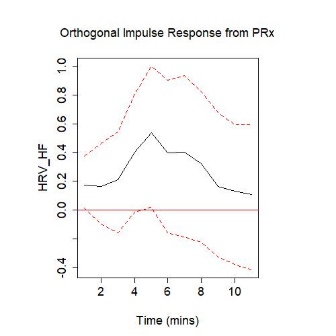

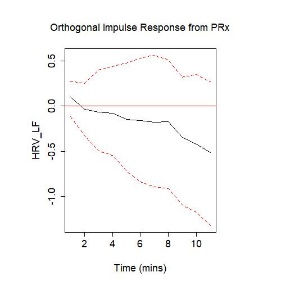

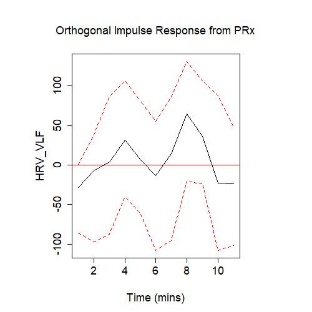

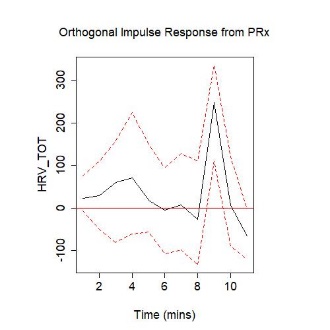

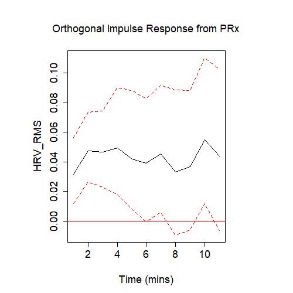
**

**Patient 2 – ARV on PRx**

**
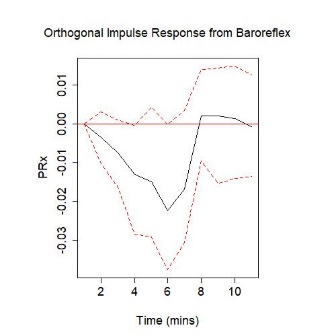

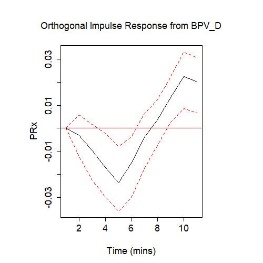

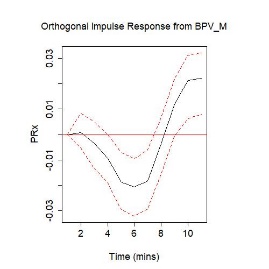

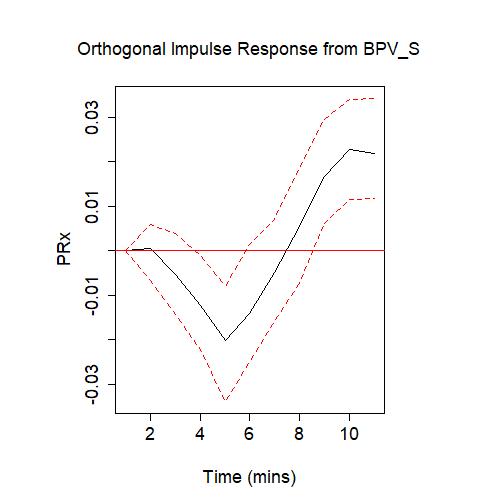

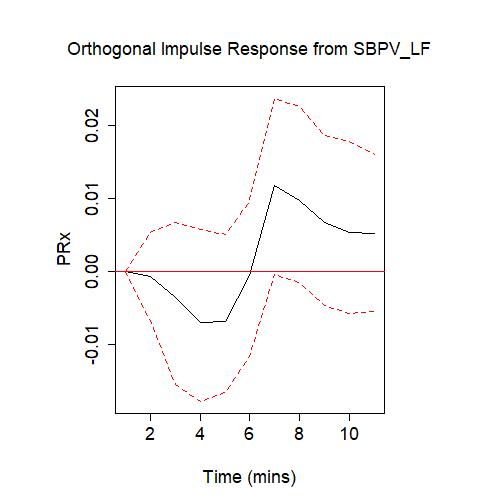

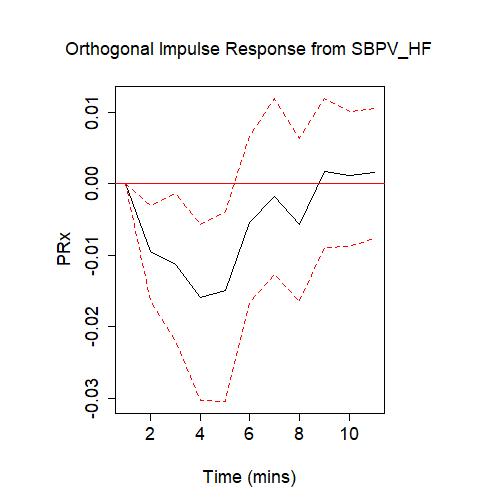

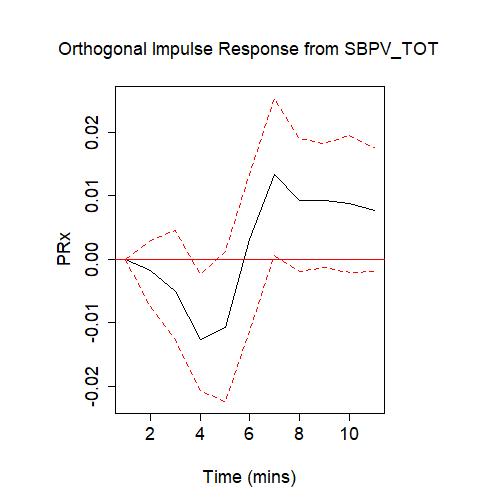

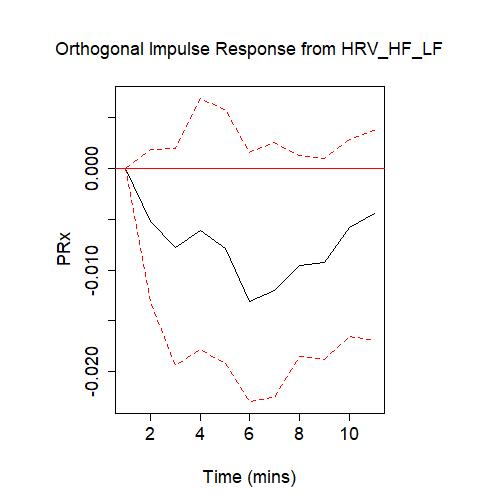

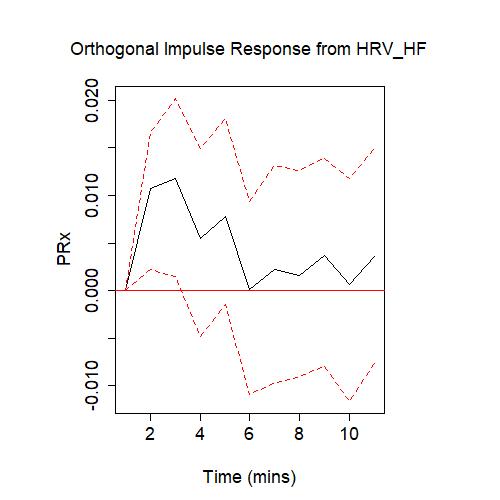

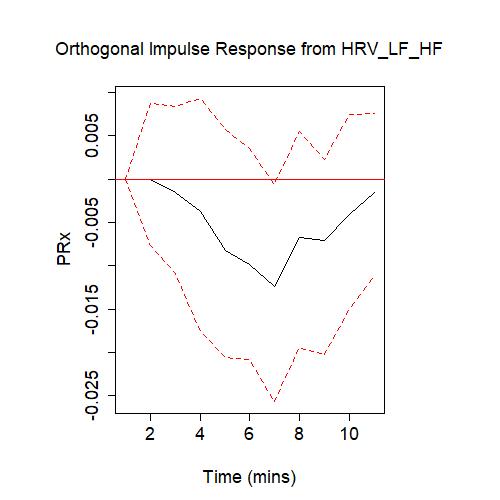

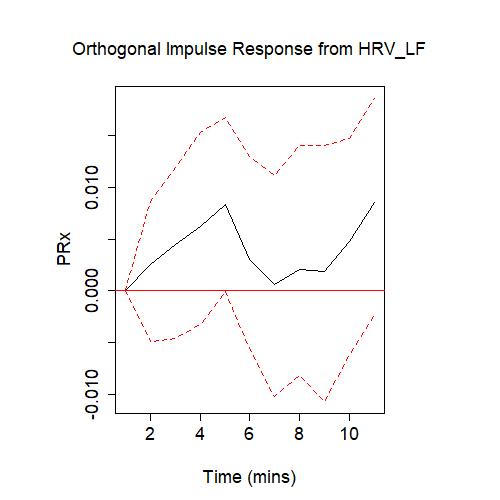

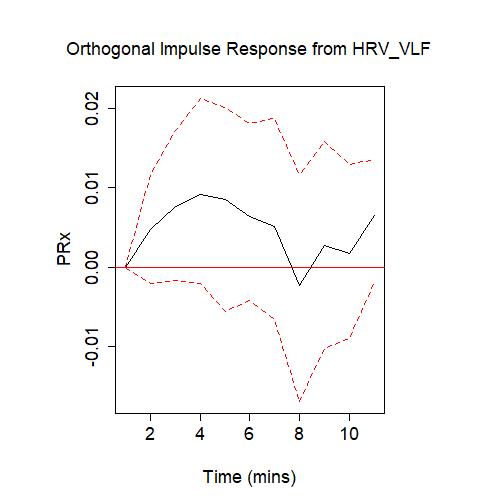

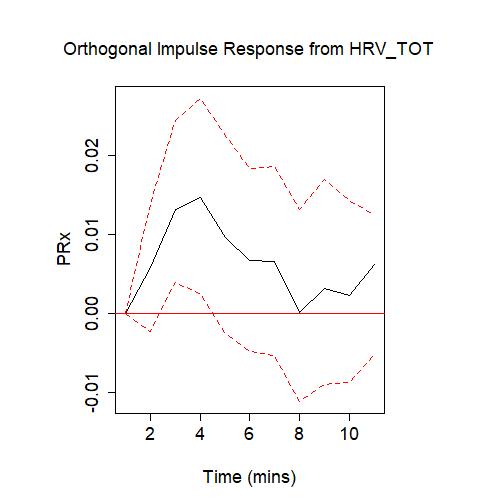

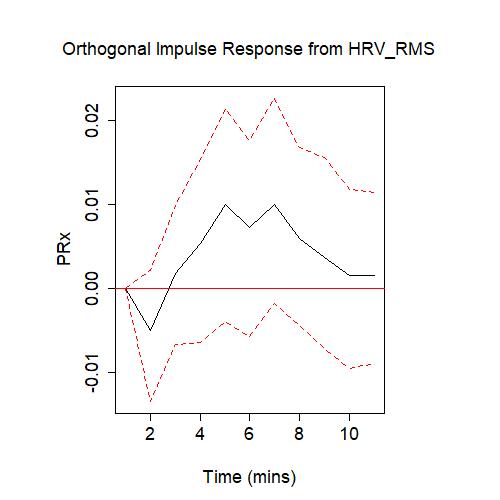
**

**Patient 2 – PRx on ARV**

**
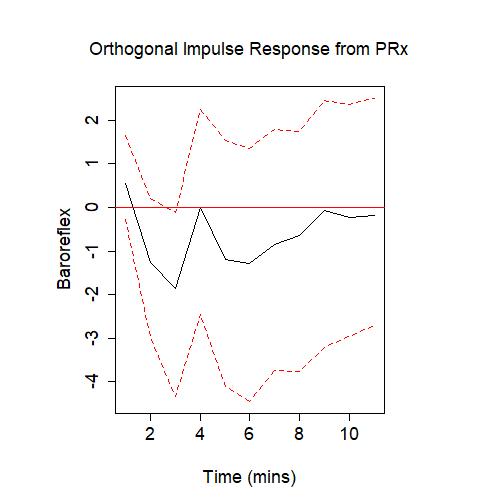

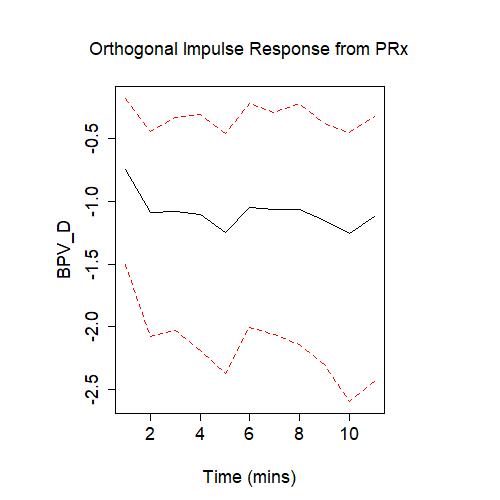

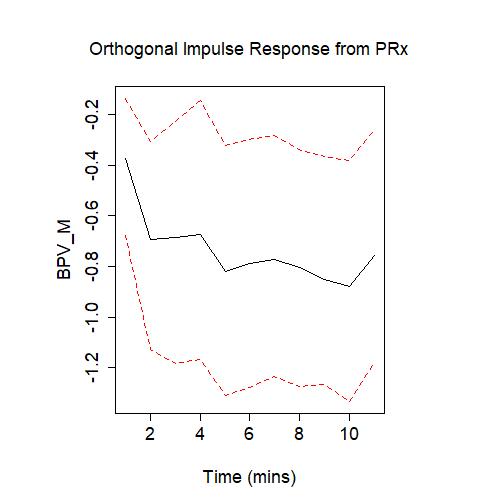

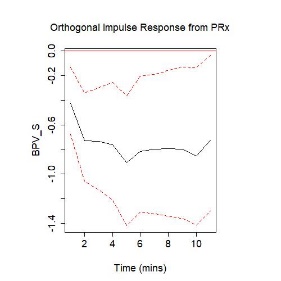

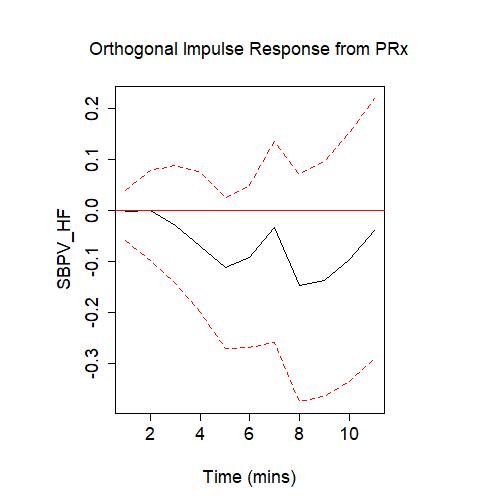

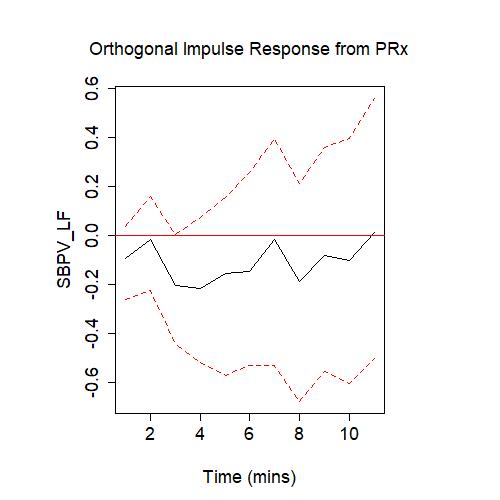

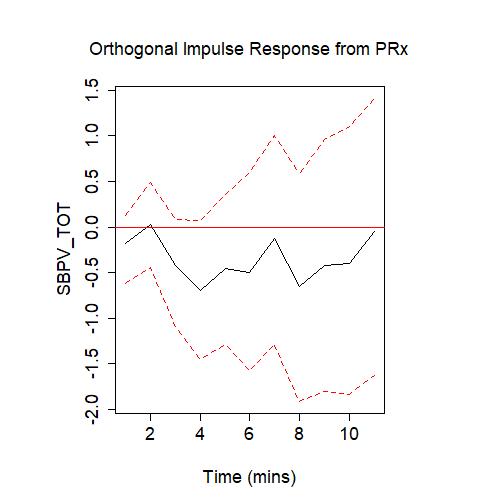

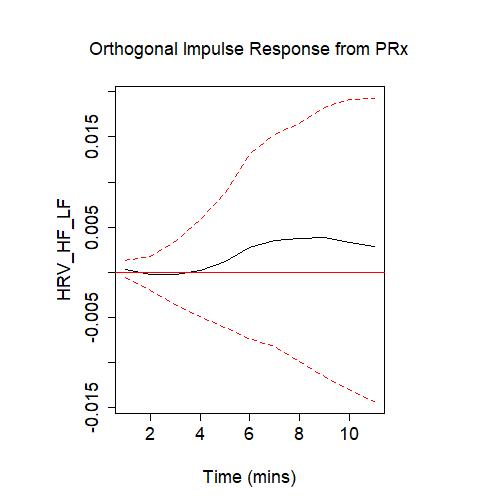

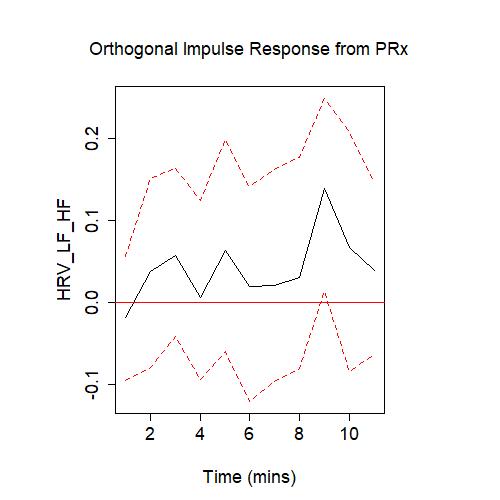

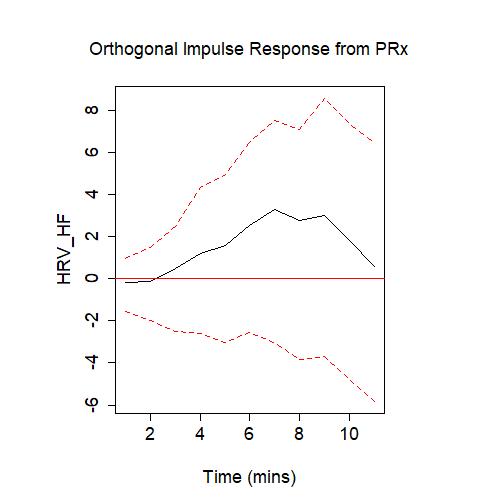

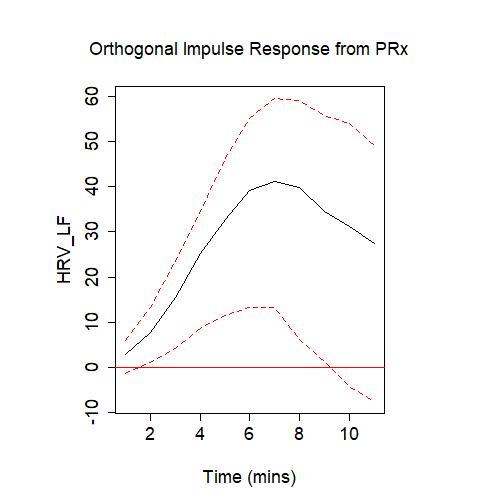

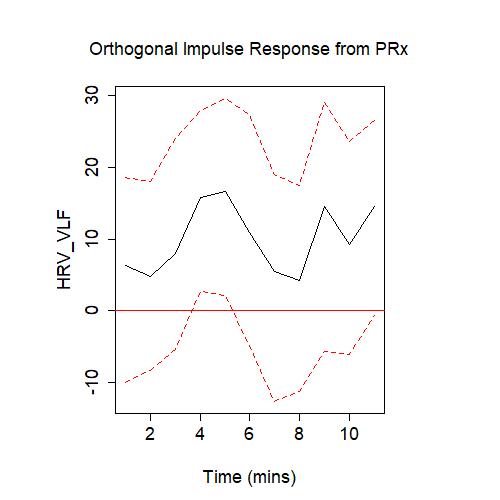

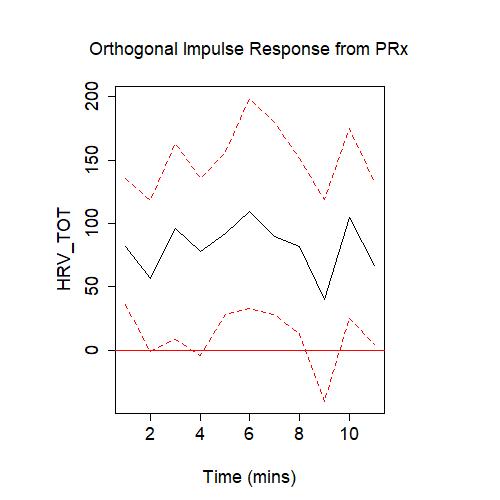

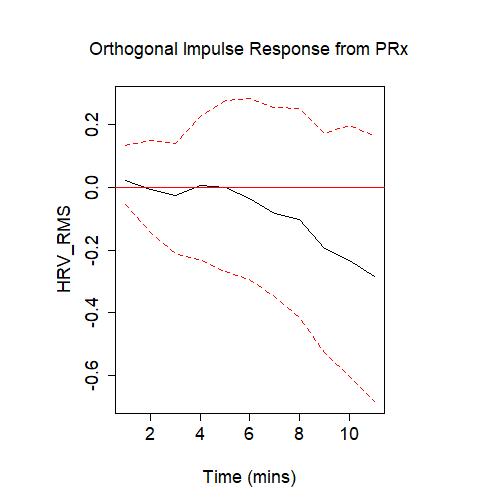
**

**Patient 4 – APV on PRx**

**
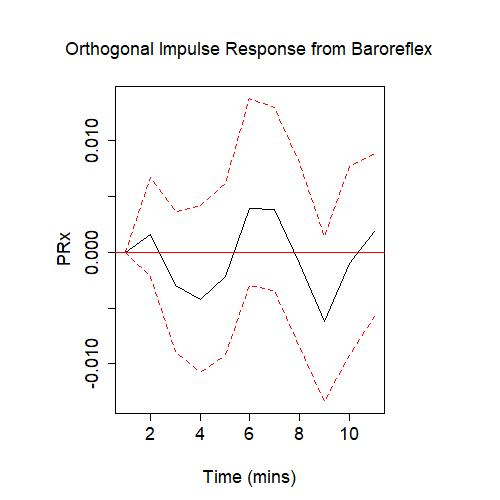

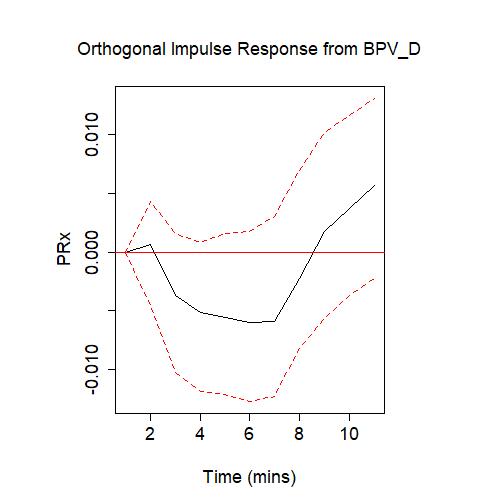

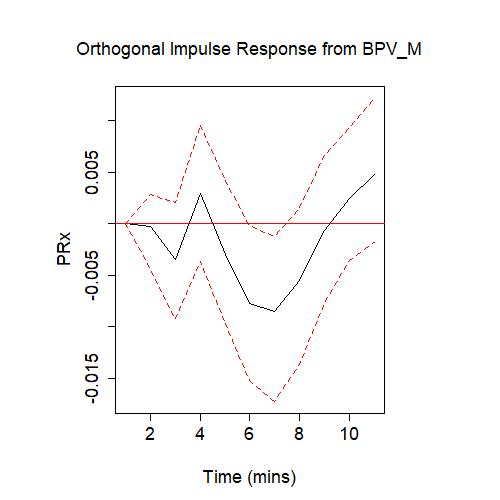

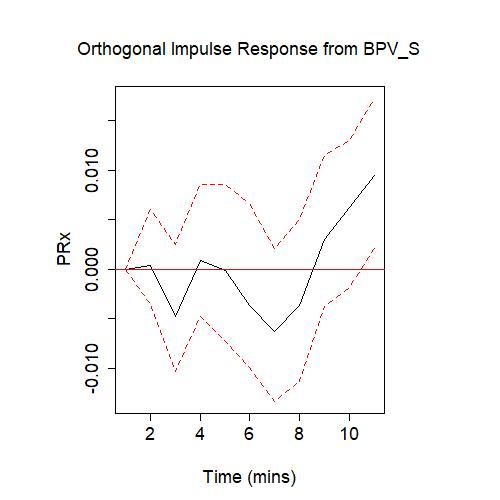

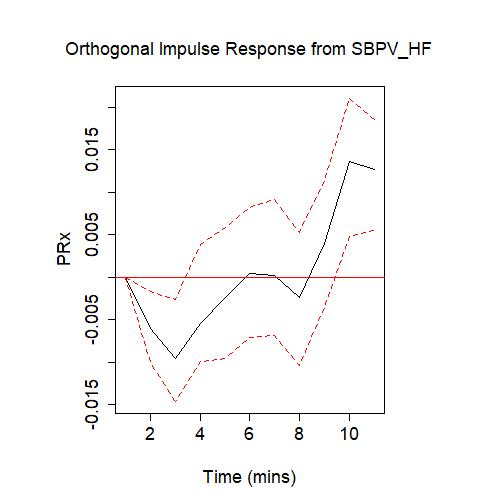

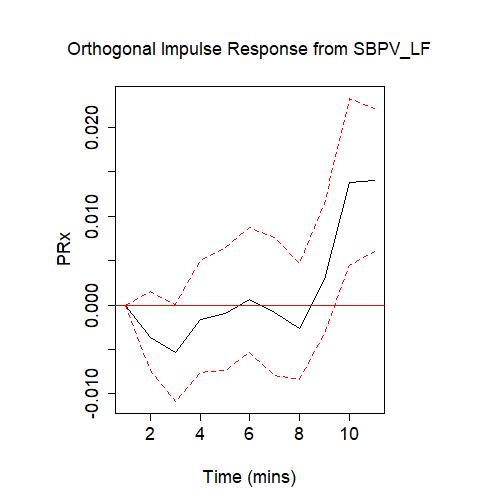

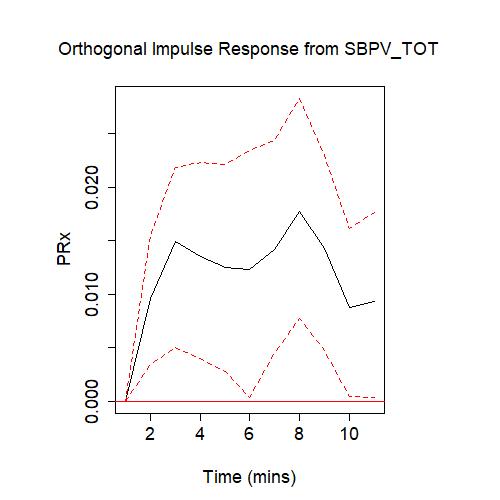

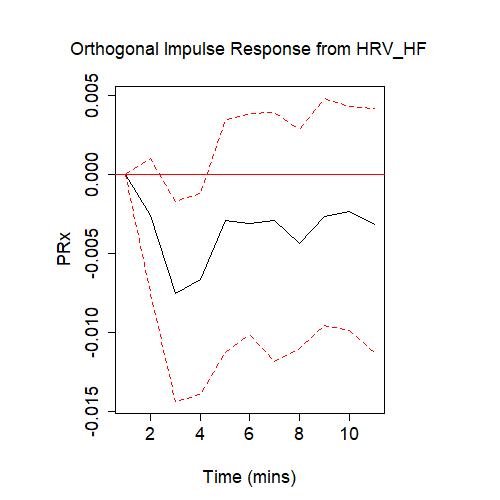

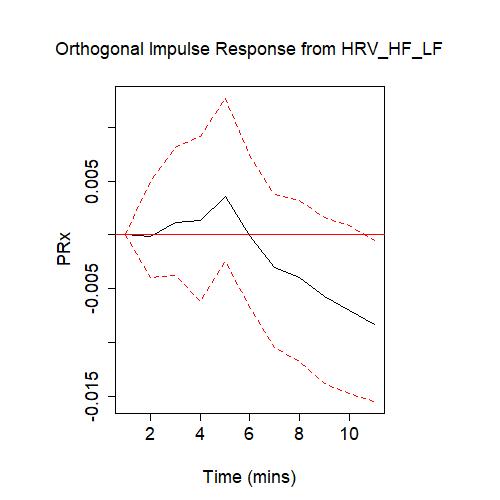

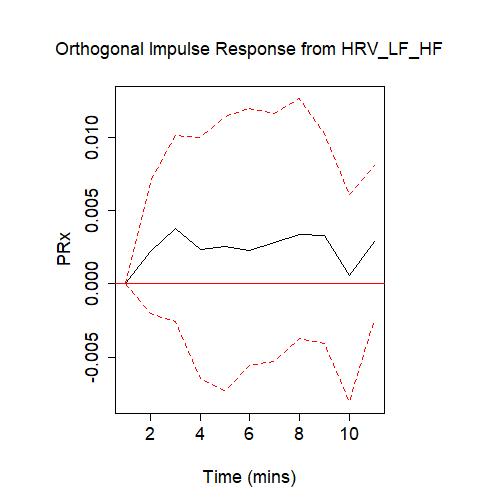

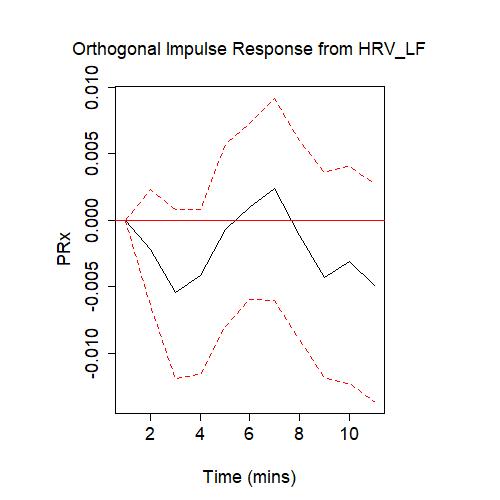

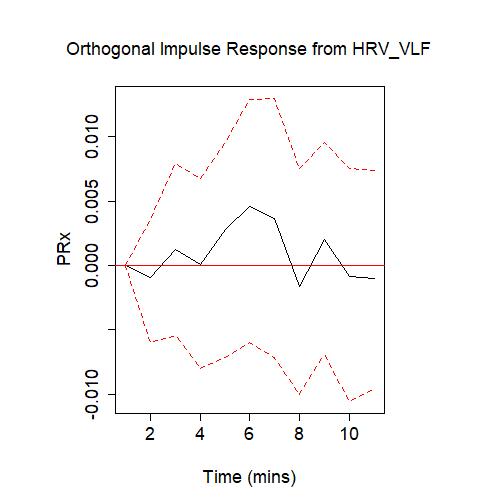

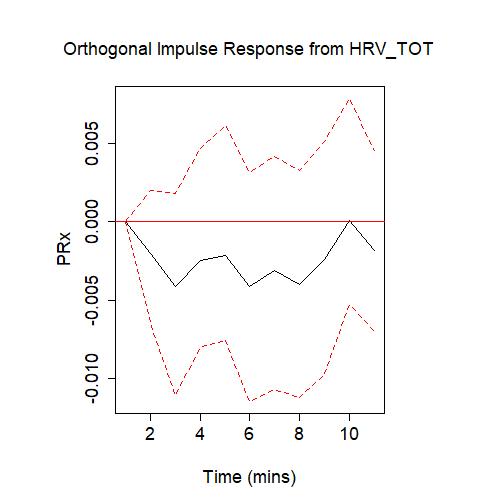

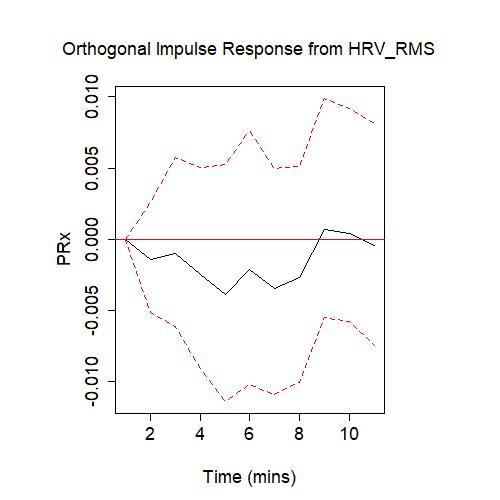
**

**Patient 4 – PRx on APV**

**
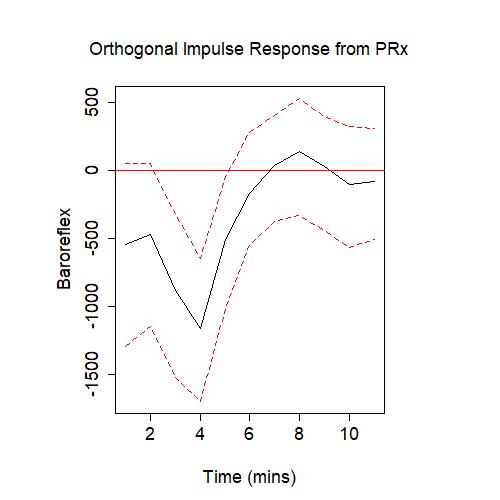

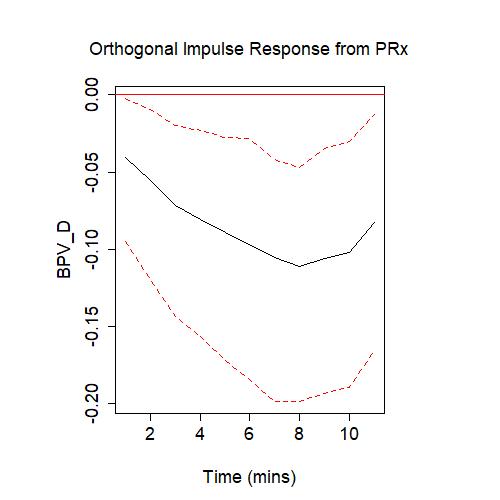

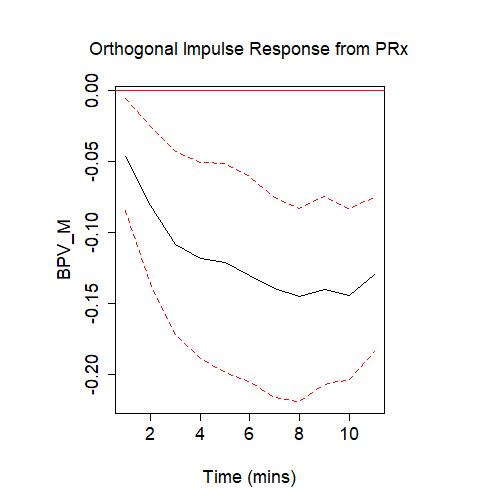

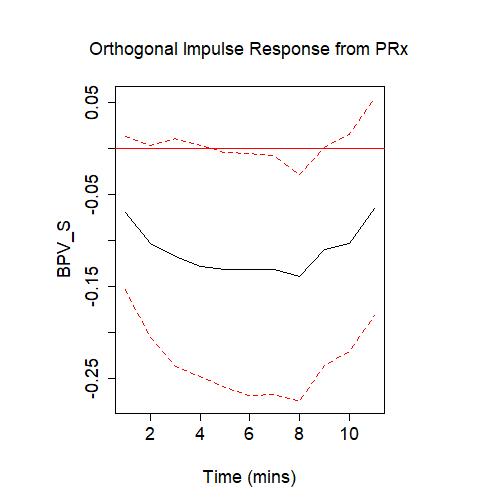

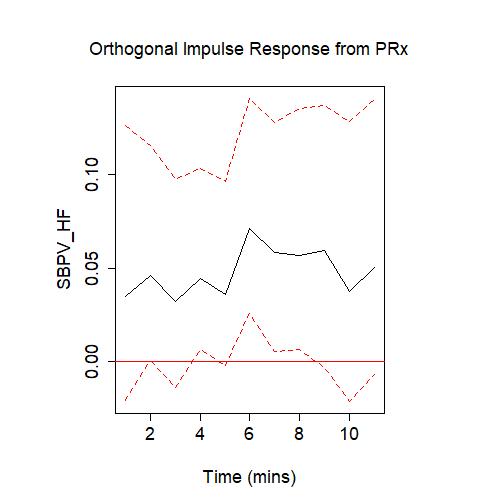

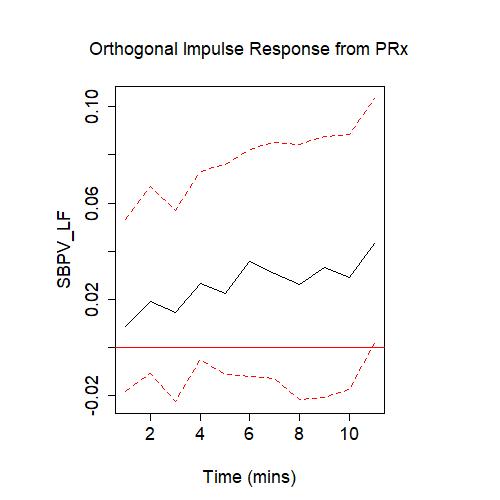

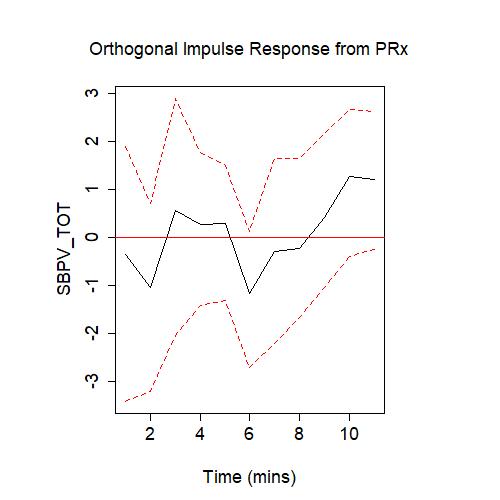

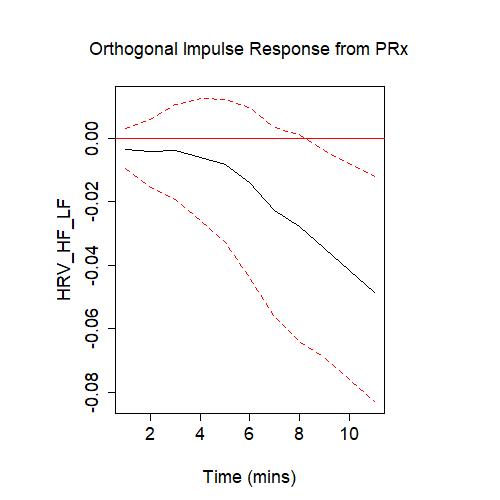

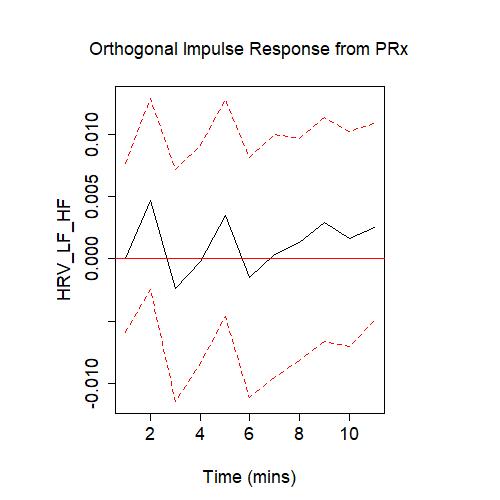

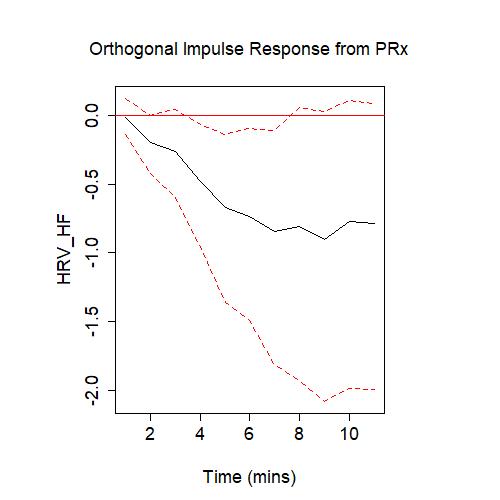

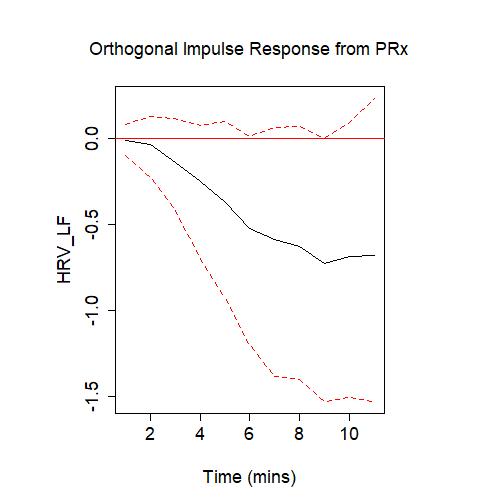

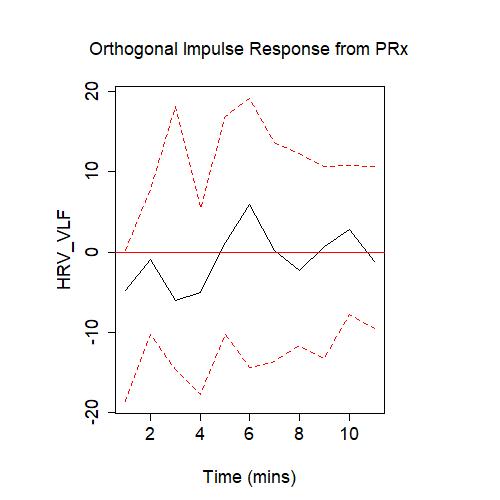

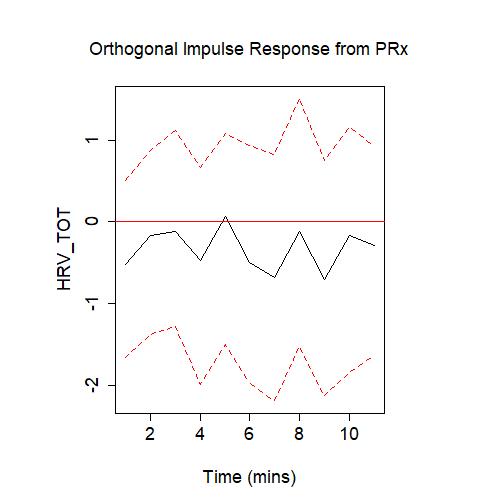

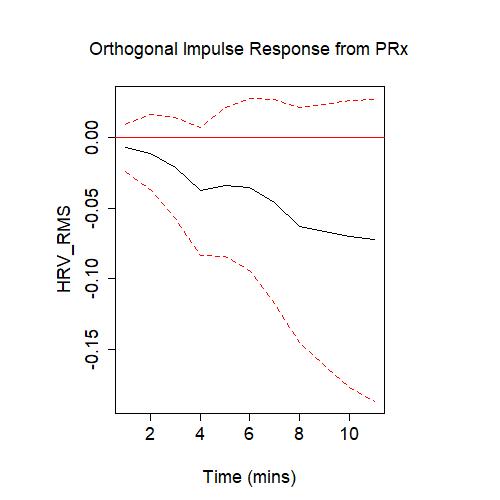
**

**Patient 5 – APV on PRx**

**
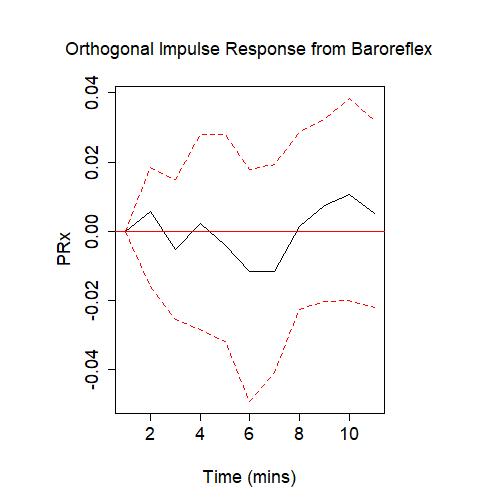

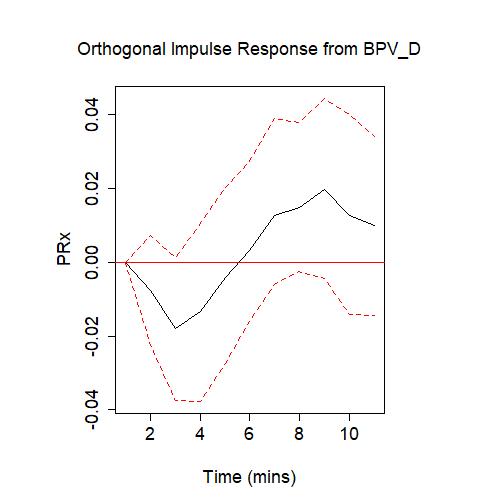

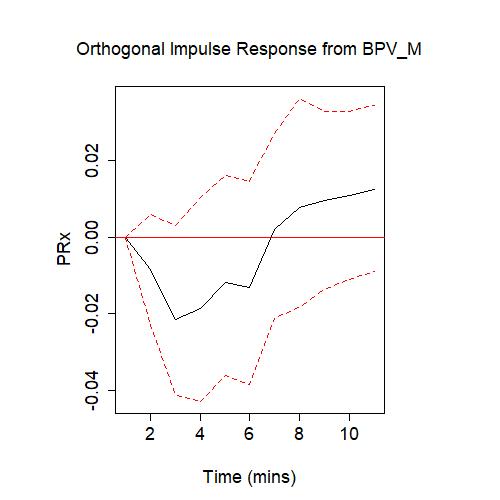

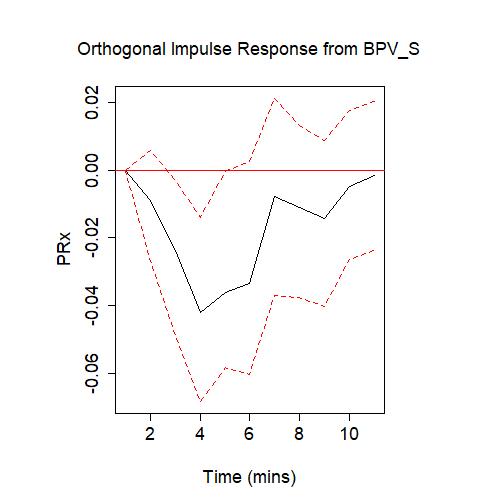

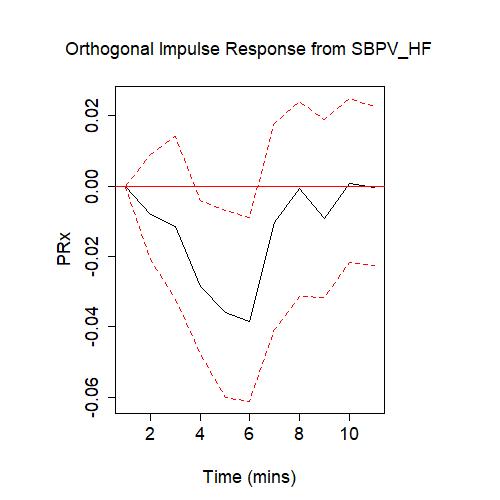

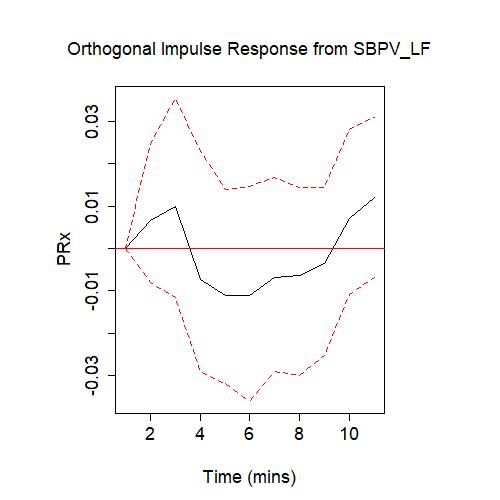

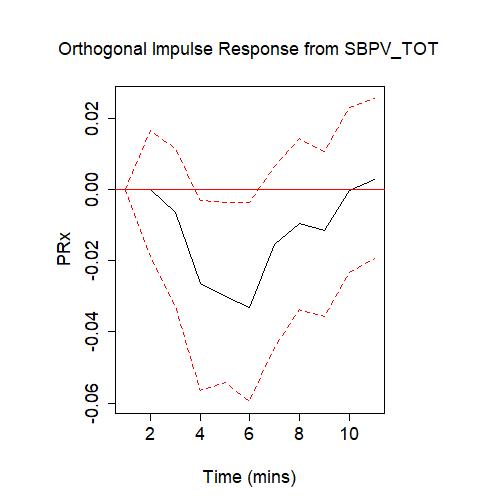

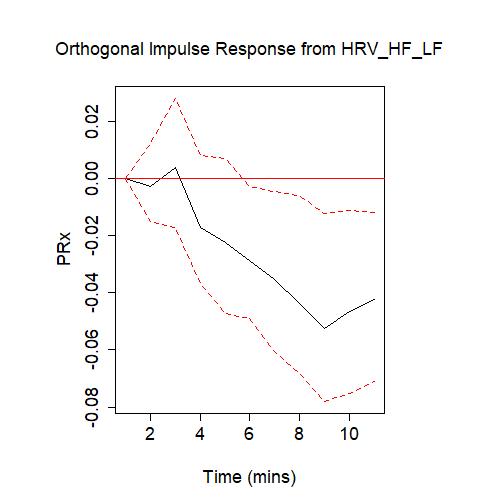

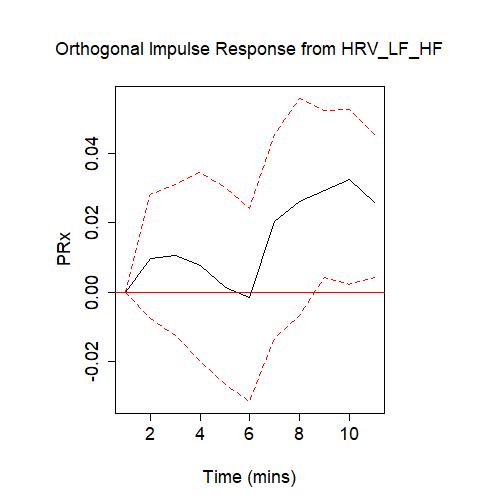

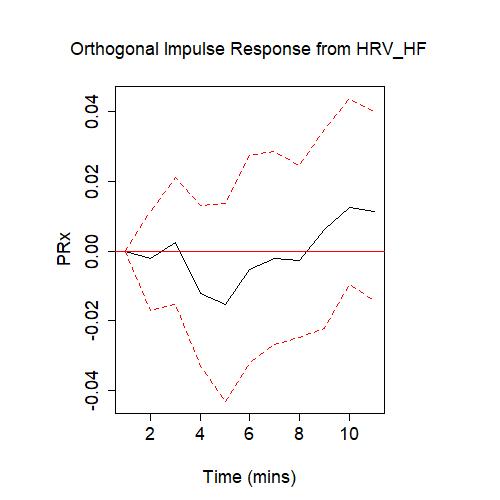

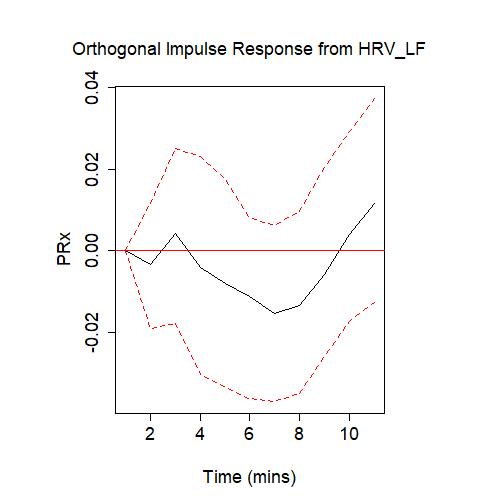

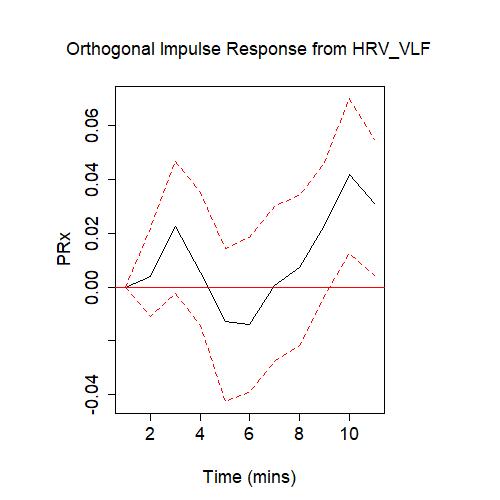

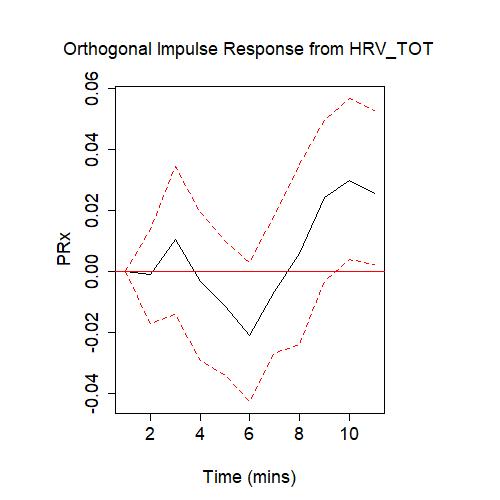

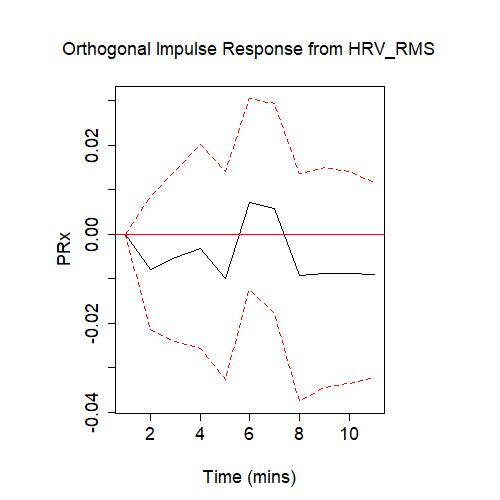
**

**Patient 5 – PRx on APV**

**
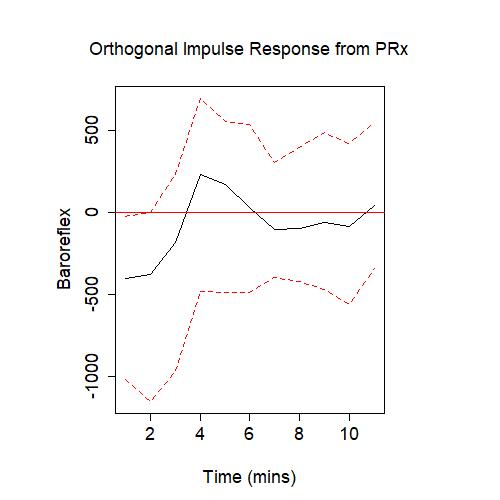

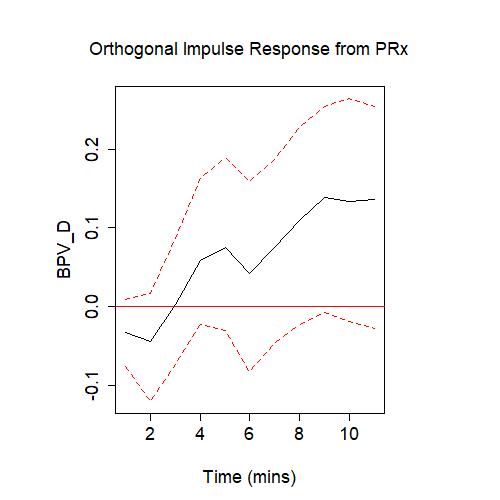
**

**Patient 6 – APV on PRx**

**Patient 6 PRx on APV**

**Patient 7 – APV on PRx**

**Patient 7 – PRx on APV**

**Patient 8 – APV on PRx**

**Patient 8 – PRx on APV**
