# Supplementary material for: Dynamic Temporal Relationship Between Autonomic Function and Cerebrovascular Reactivity in Moderate/Severe Traumatic Brain Injury
Source: Front Netw Physiol. 2022 Feb 16;2:837860. doi: 10.3389/fnetp.2022.837860 (PMC10013014; doi:10.3389/fnetp.2022.837860)

Appendix F. Dendrogram and Cophenetic Correlation for All Patients (n=47)

*The cophenetic correlation is a measure of the similarity between two variables and is a comparison of the pairwise distances between the original data points.* *The distance between two variables shows the height of the dendrogram where the two branches merge into a single branch, thus two variables that diverge on the last branch may be more closely linked. BPV_D, standard deviation of diastolic blood pressure variability; BPV_M, standard deviation of mean blood pressure variability; BPV_S, standard deviation of systolic blood pressure variability; BRS, baroreflex sensitivity; HRF_HF, heart rate variability high frequency; HRV_HF_LF, heart rate variability ratio between high/low frequency; HRV_LF, heart rate variability low frequency; HRV_LF_HF, heart rate variability ratio between low/high frequency; HRV_RMS, heart rate variability root mean square; HRV_TOT, heart rate variability total; HRV_VLF, heart rate variability very low frequency; PRx, pressure reactivity; SBPV_HF, spectral blood pressure variability high frequency; SBPV_LF, spectral blood pressure variability low frequency; SBPV_TOT, spectral blood pressure variability total;*
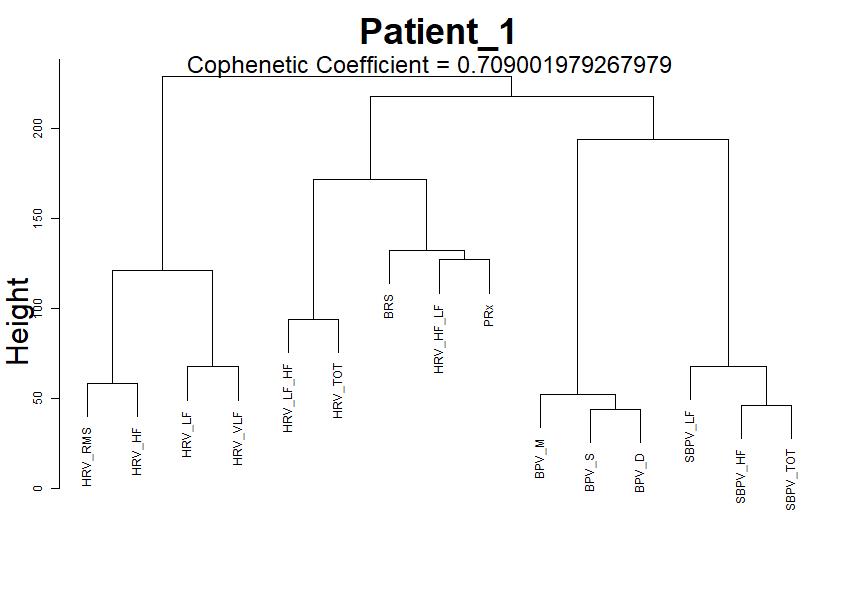

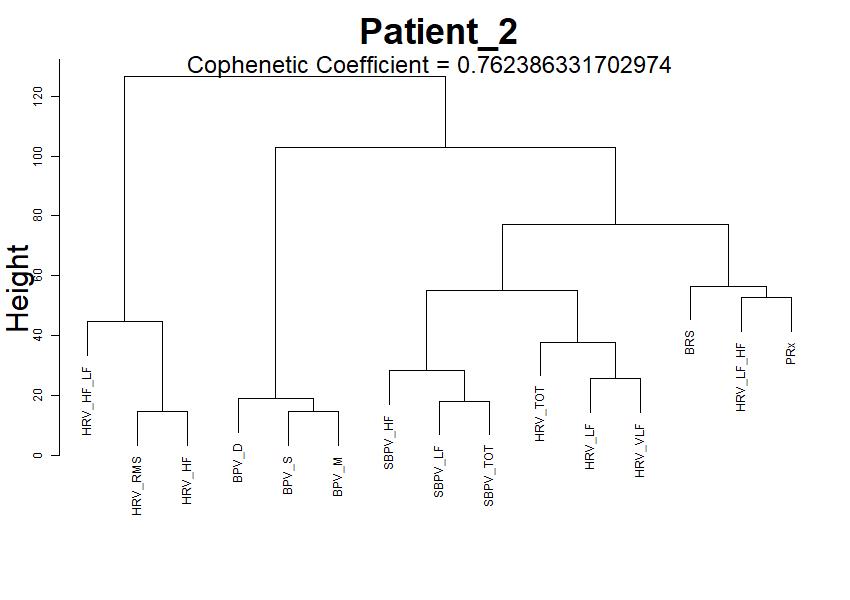

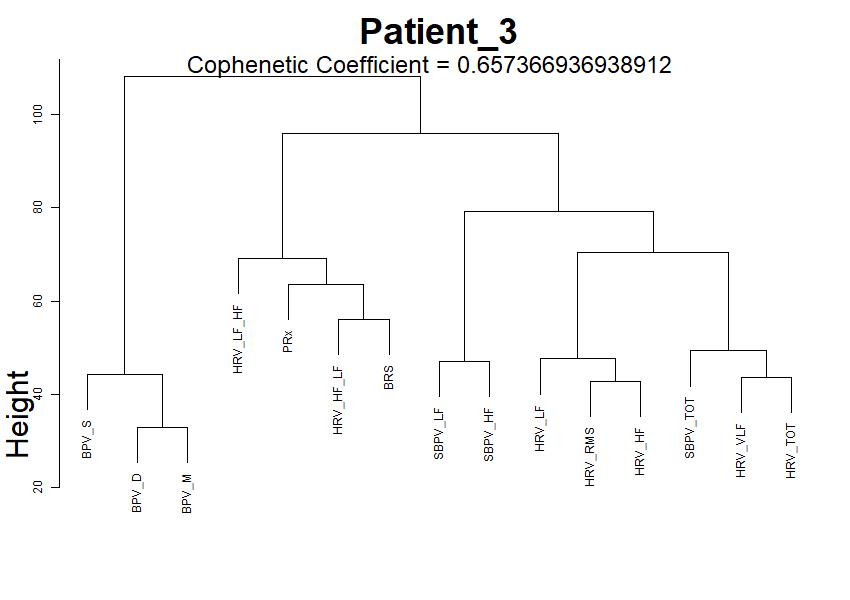

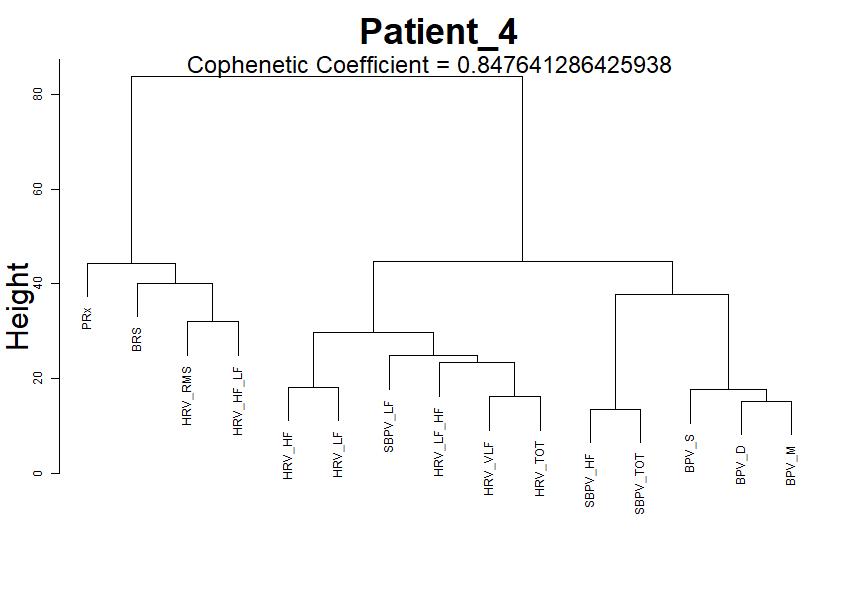

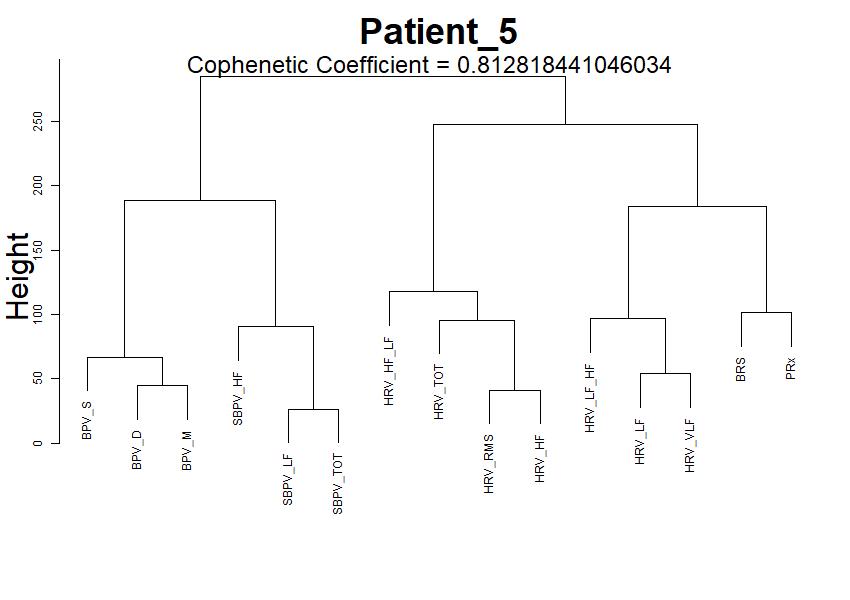

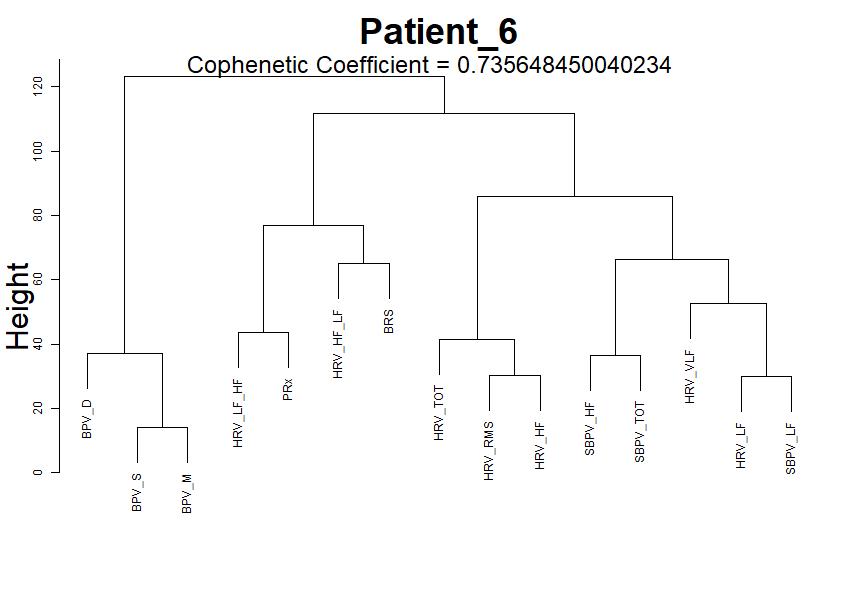

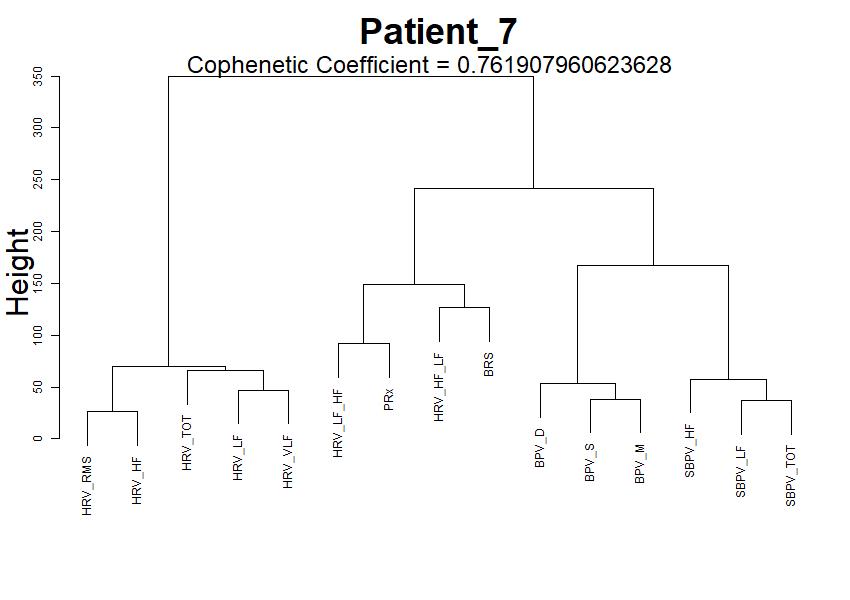

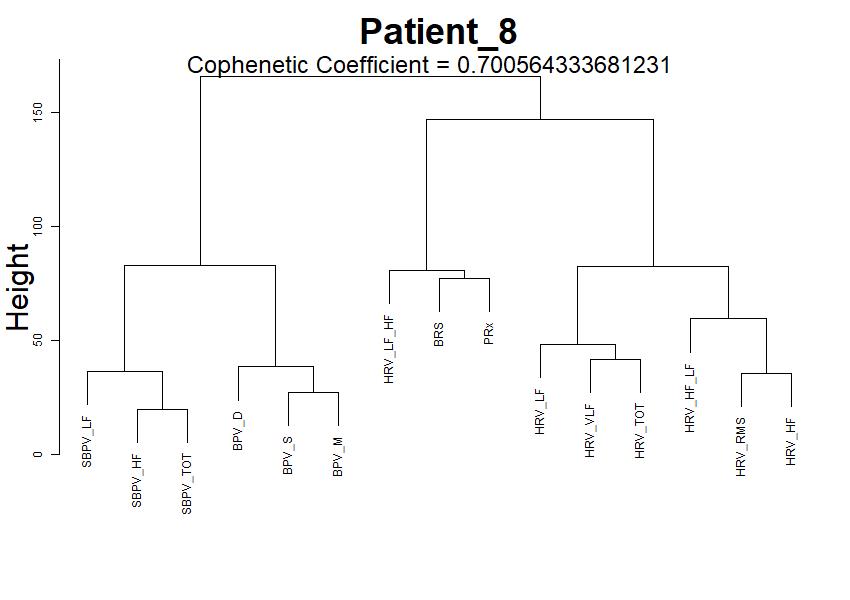

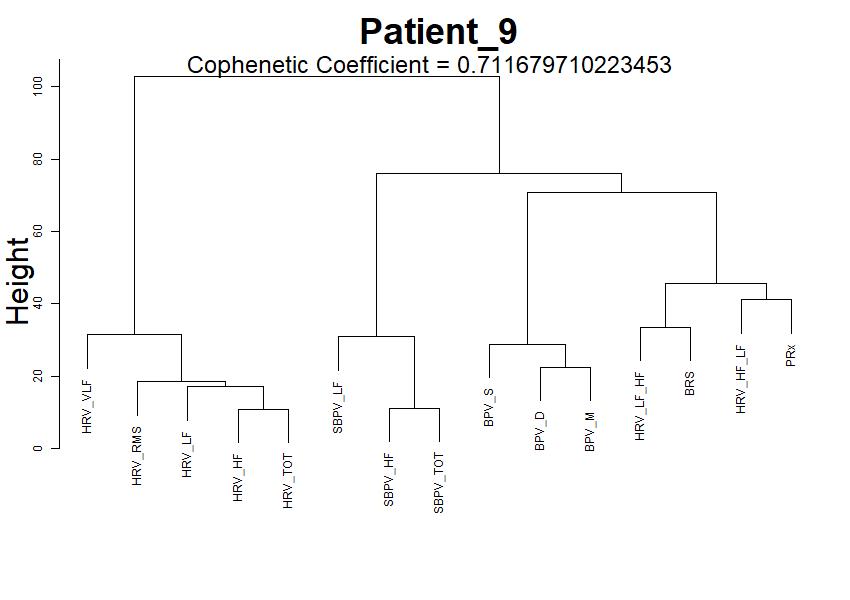

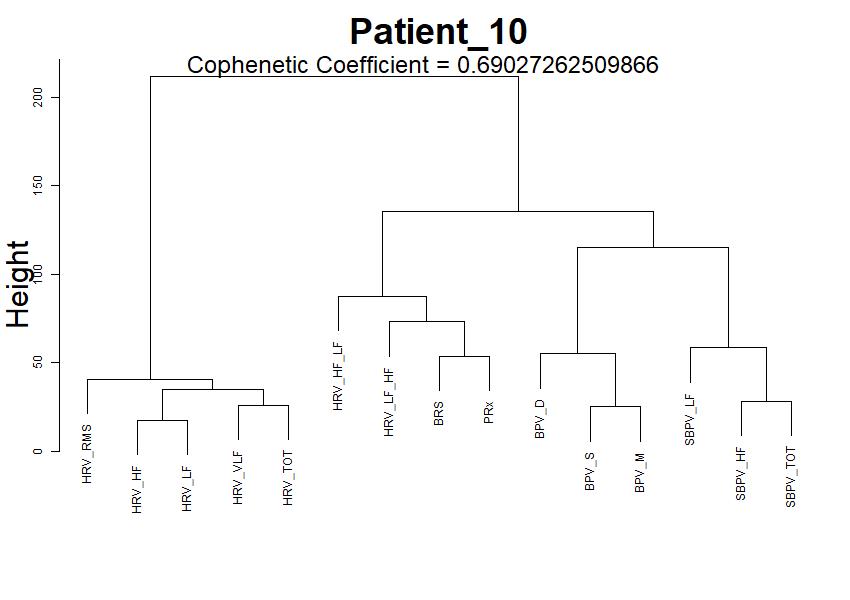

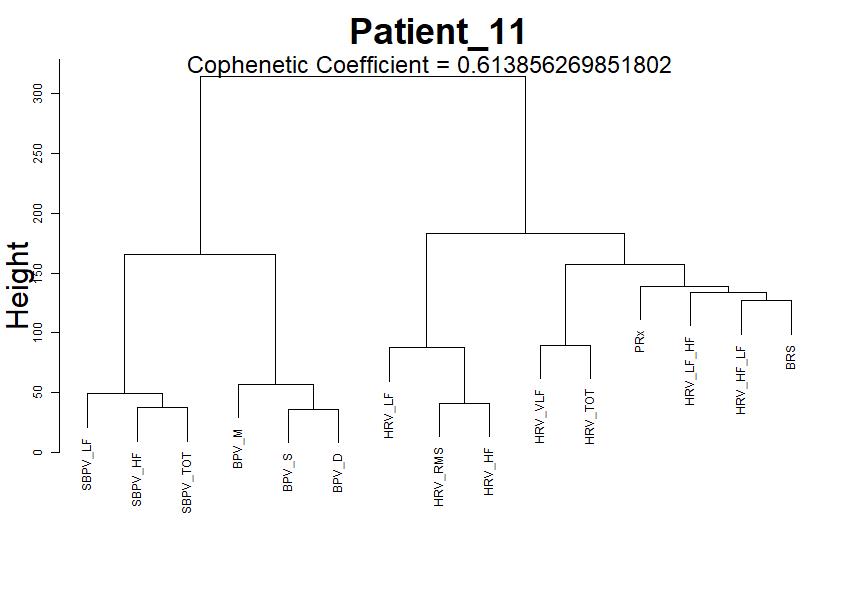

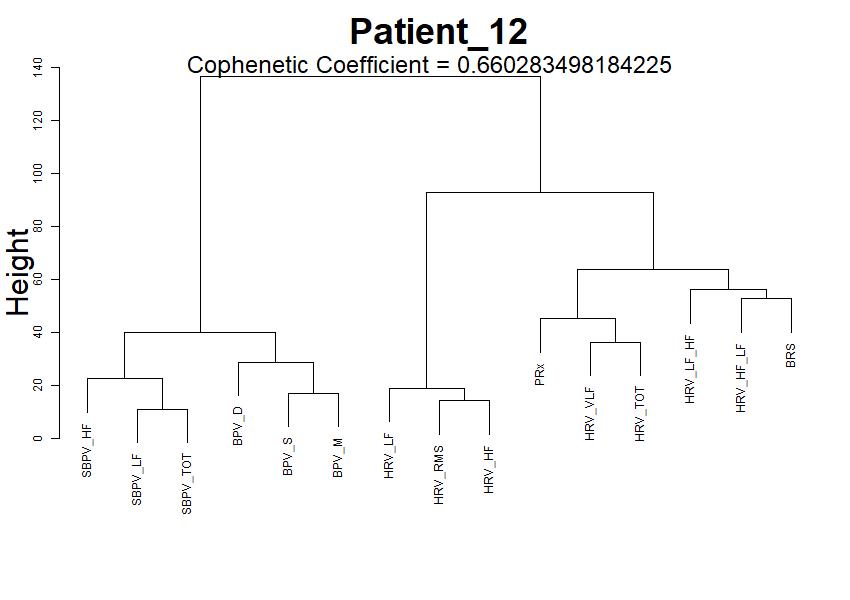

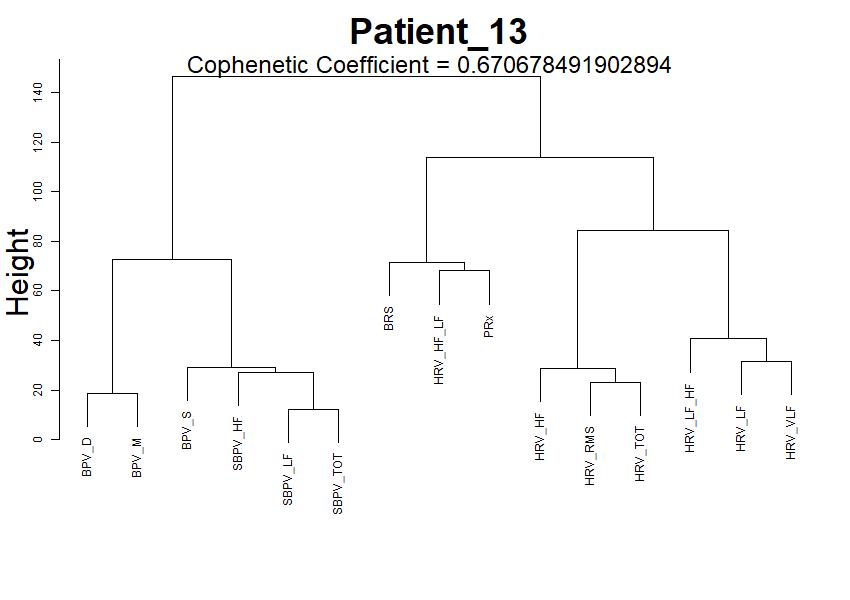

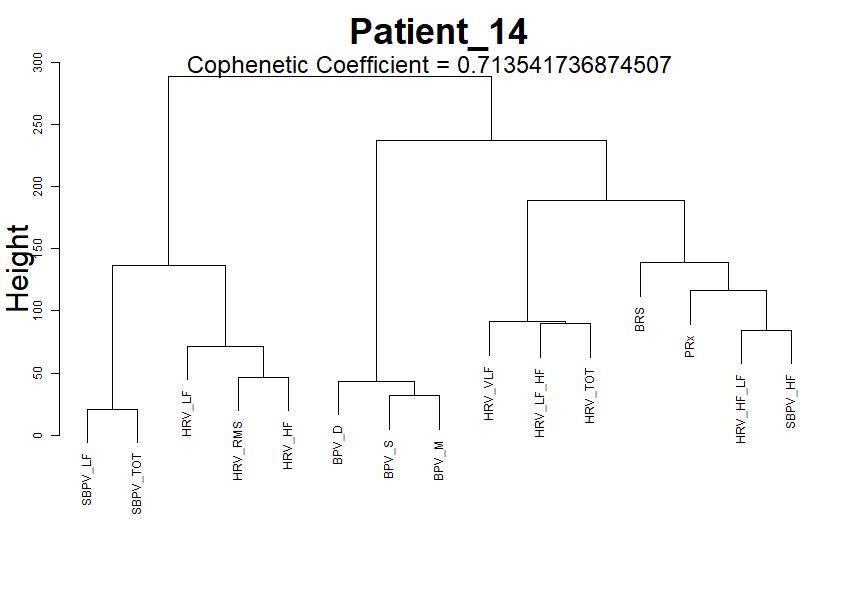

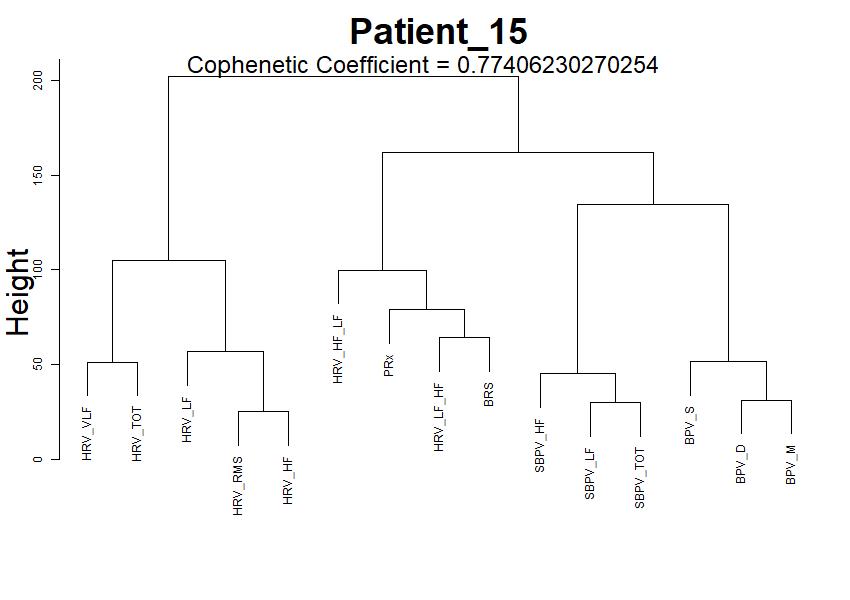

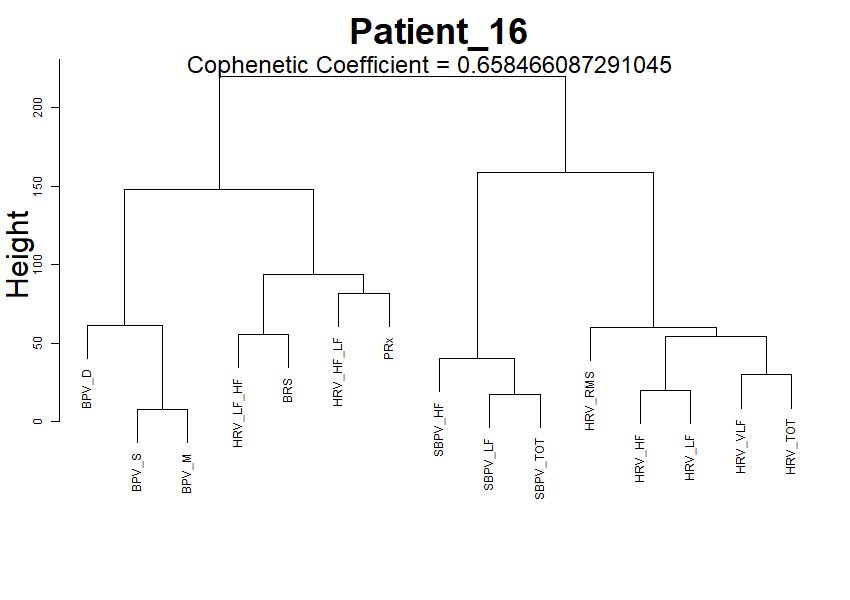

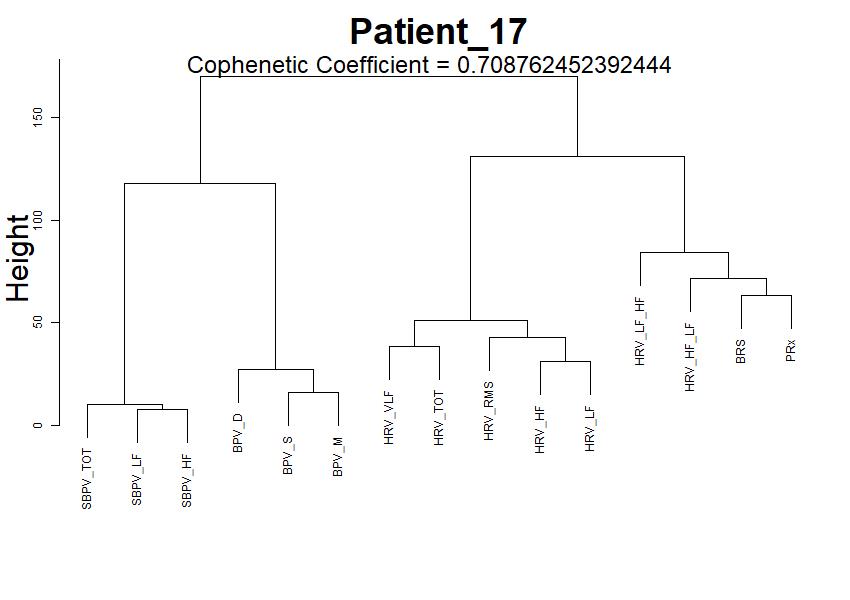

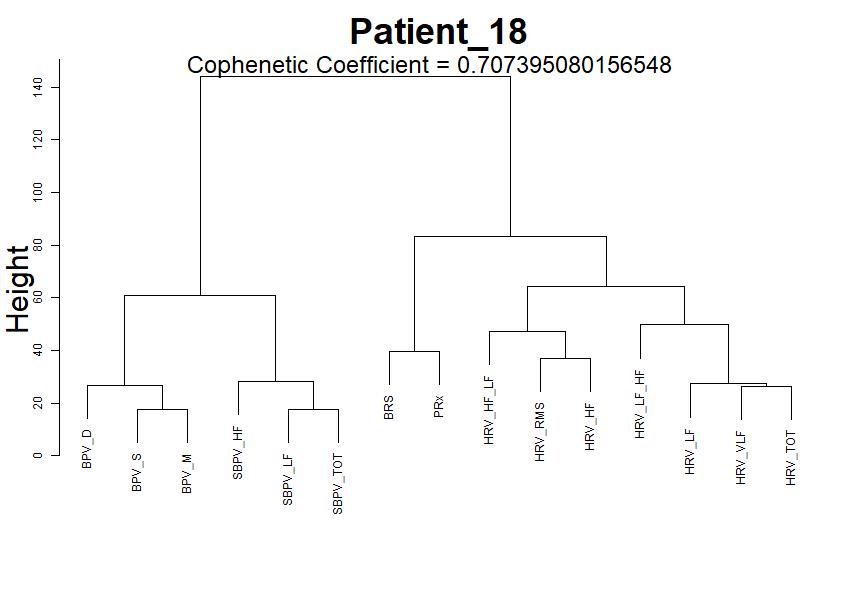

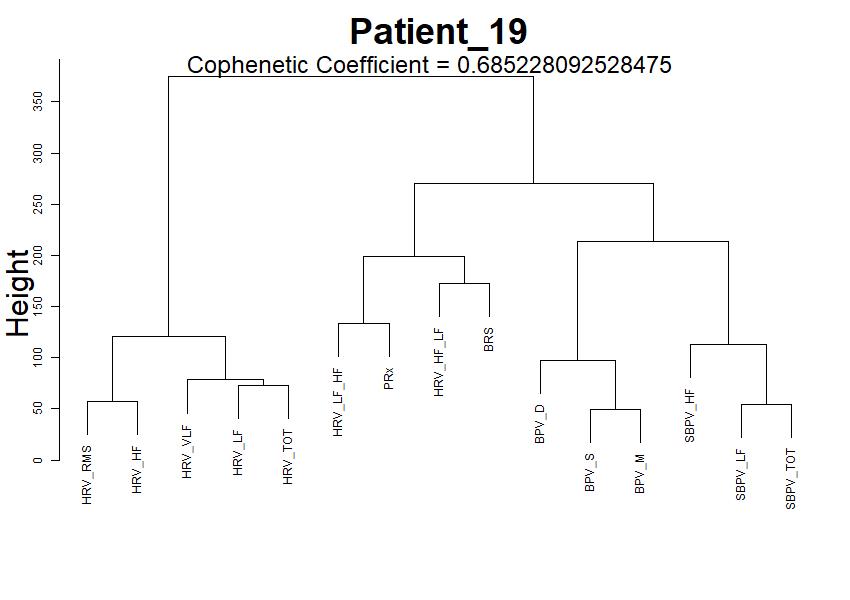

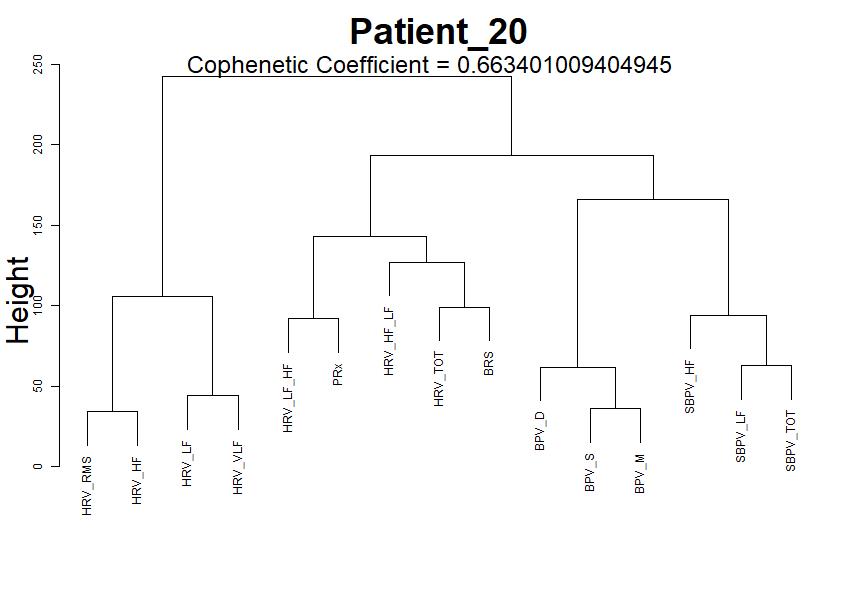

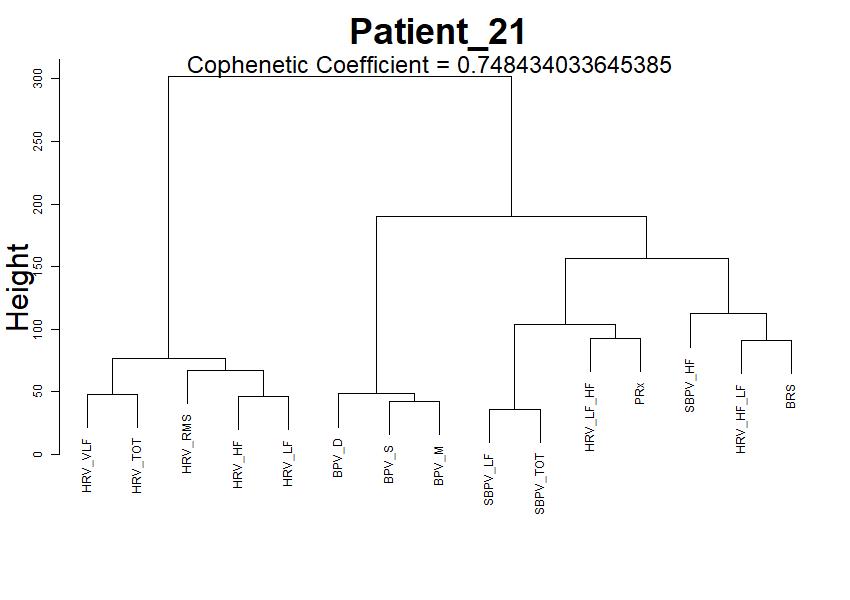

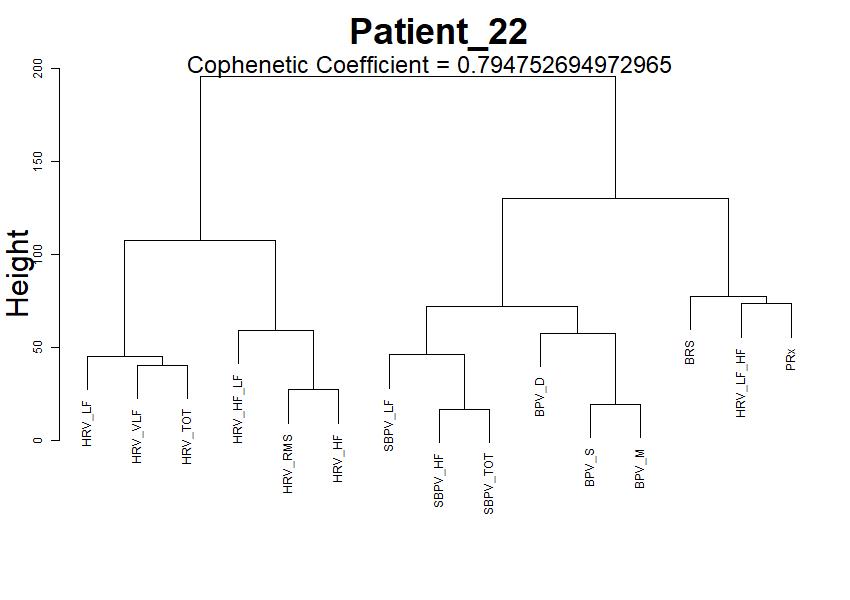

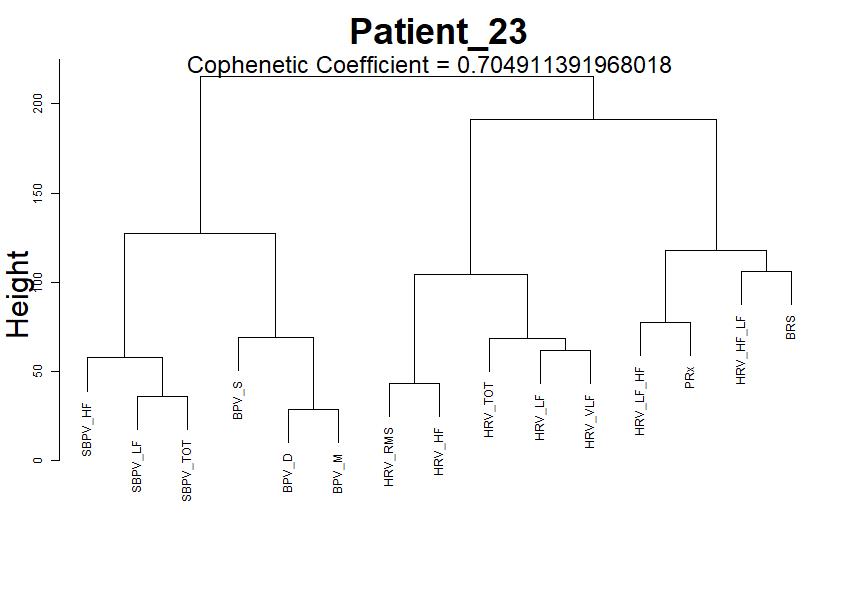

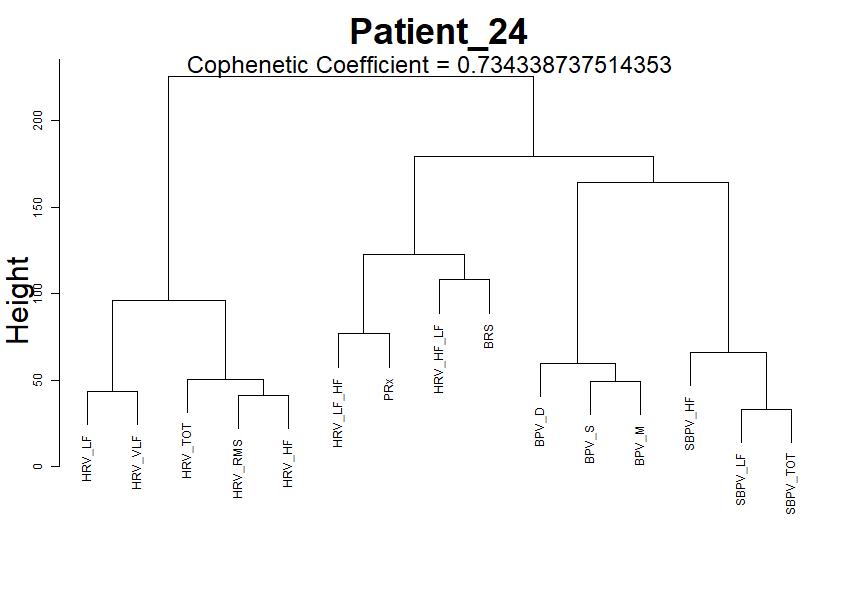

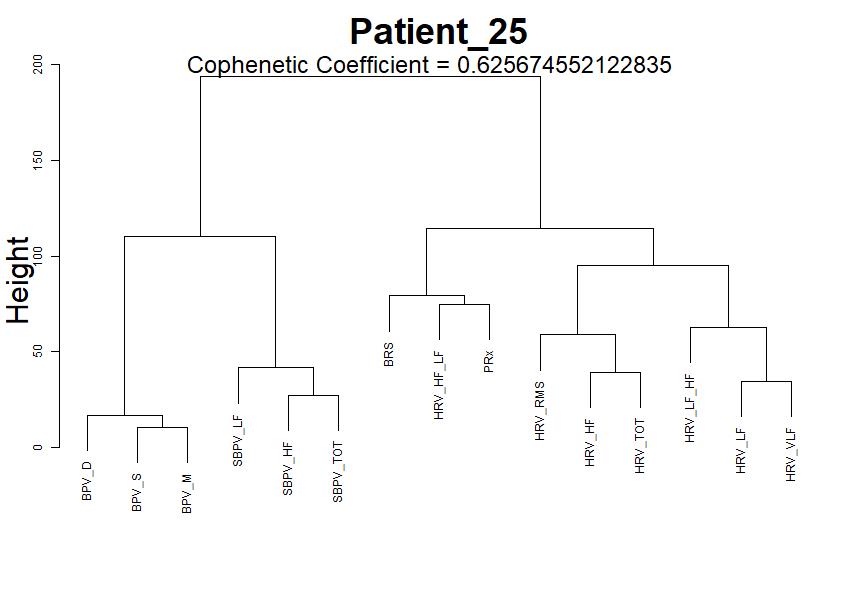

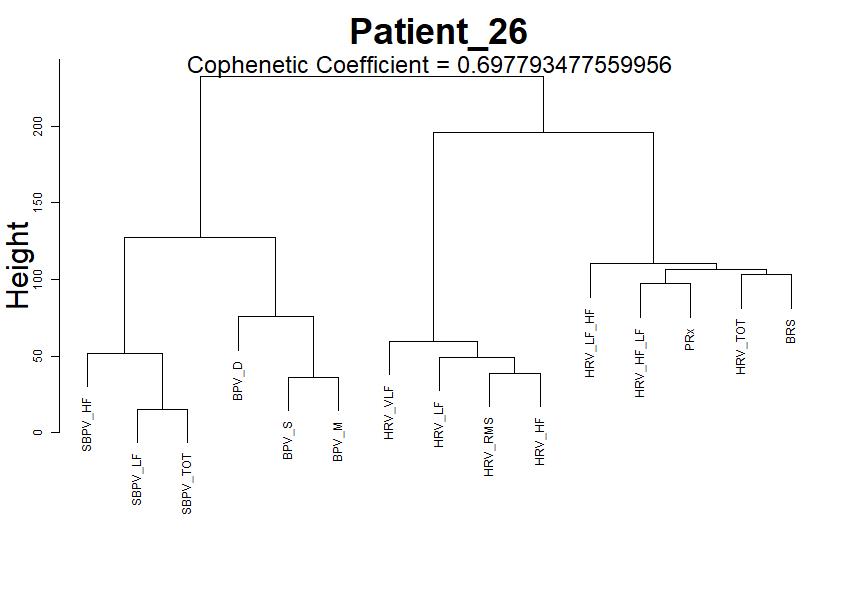

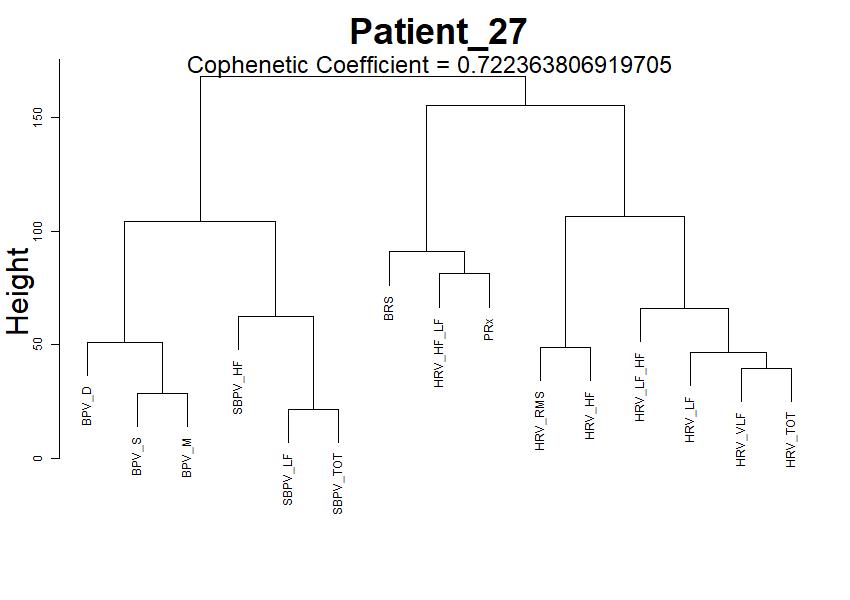

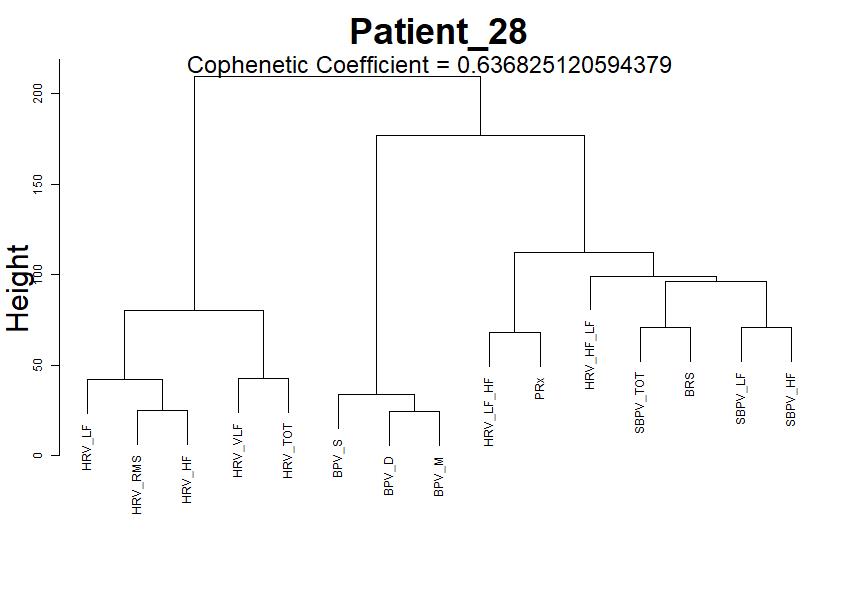

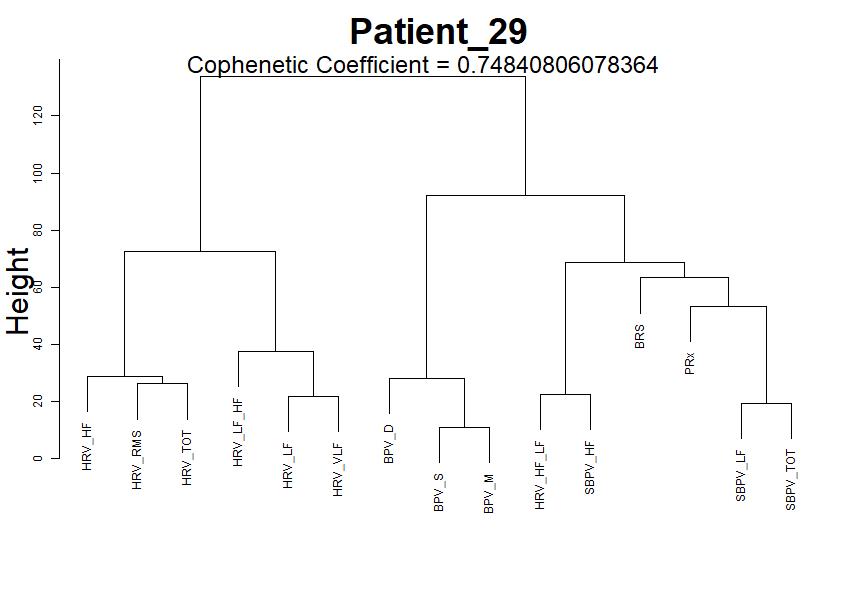

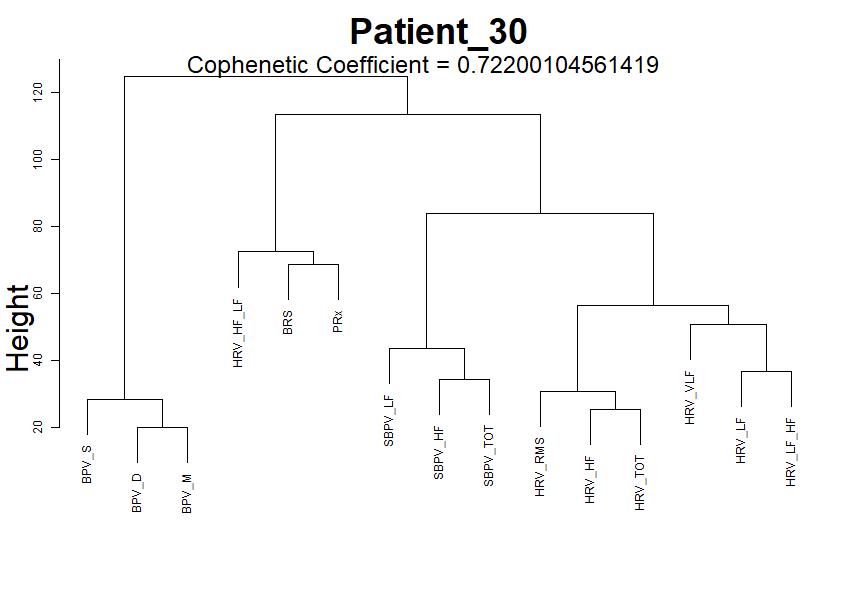

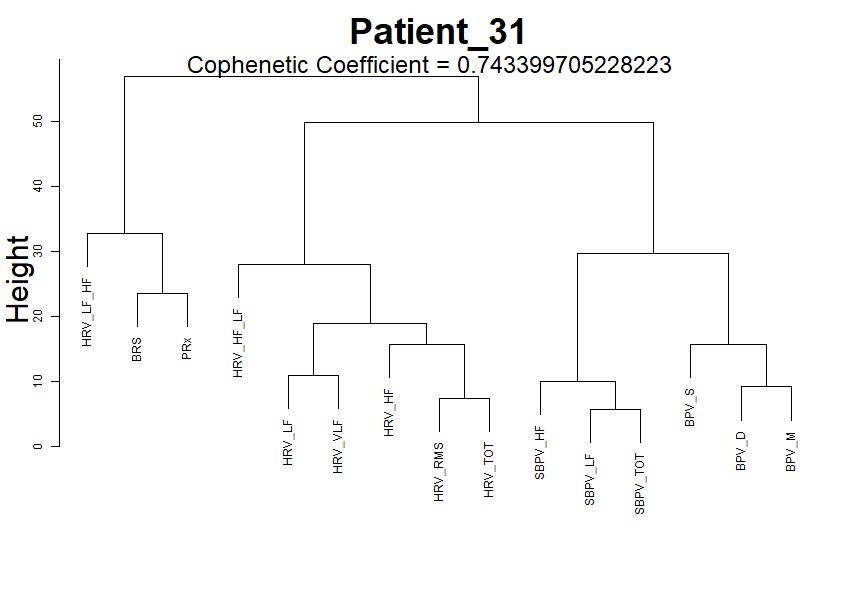

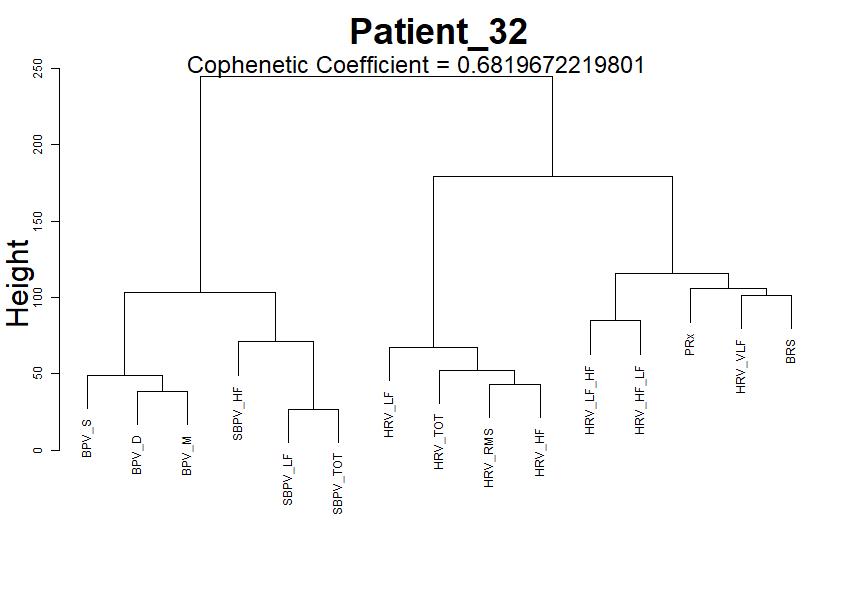

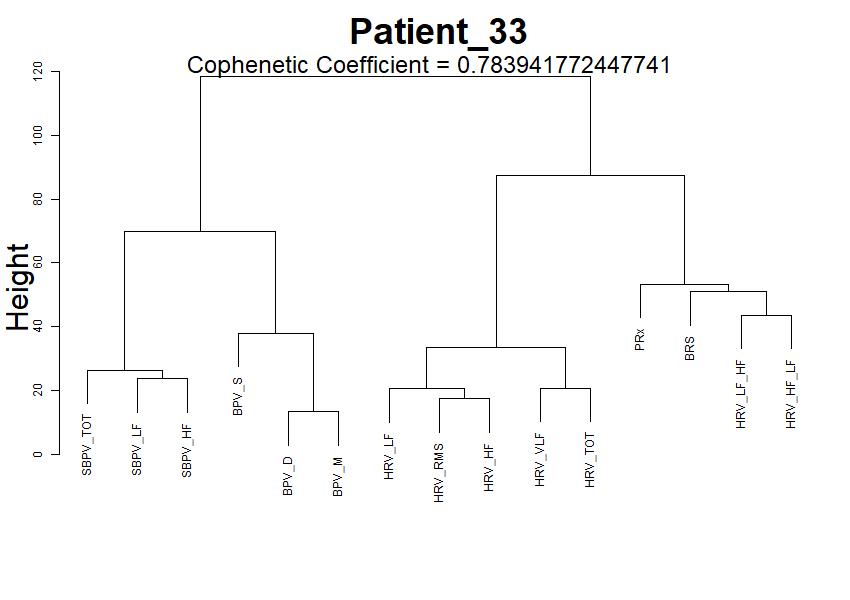

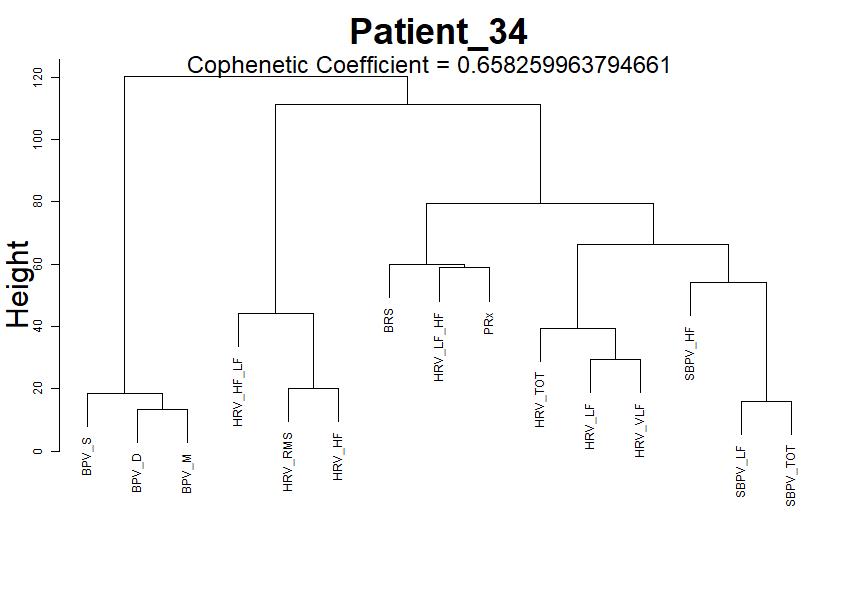

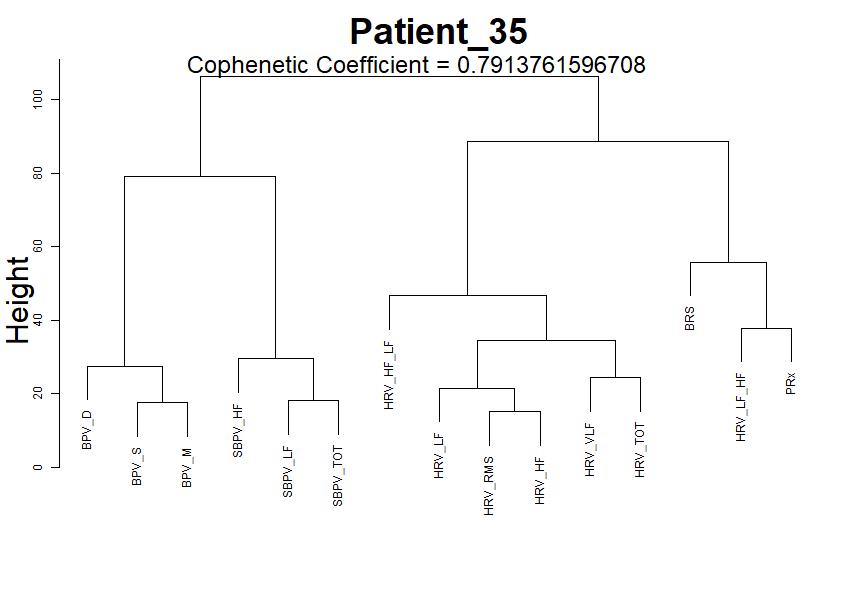

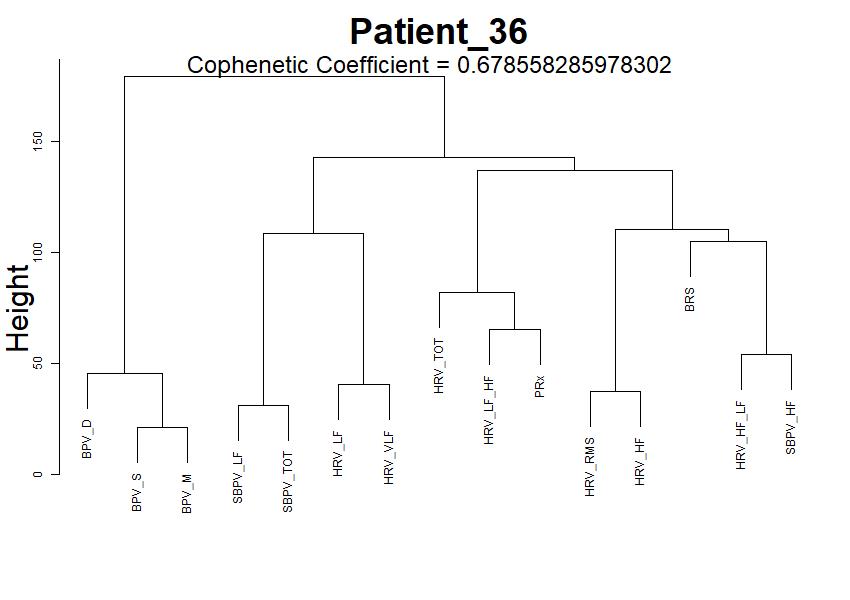

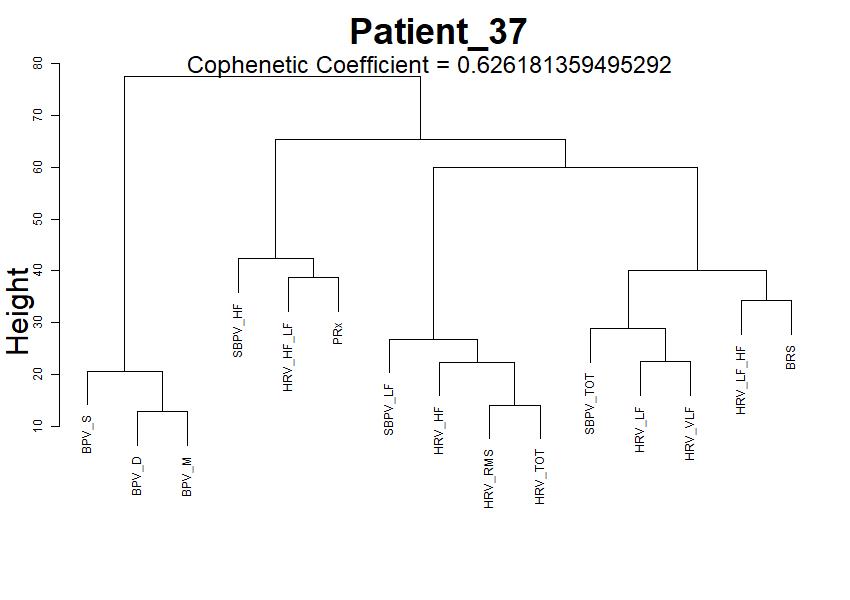

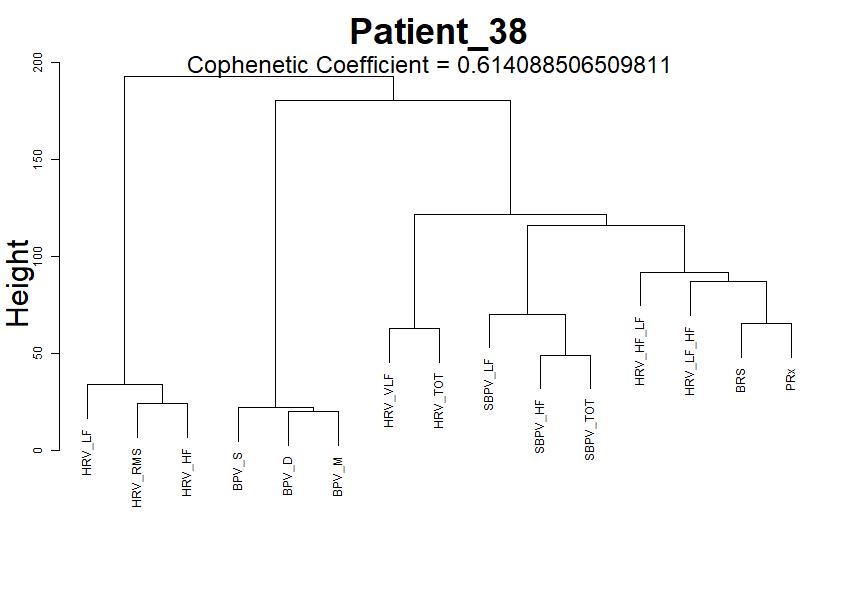

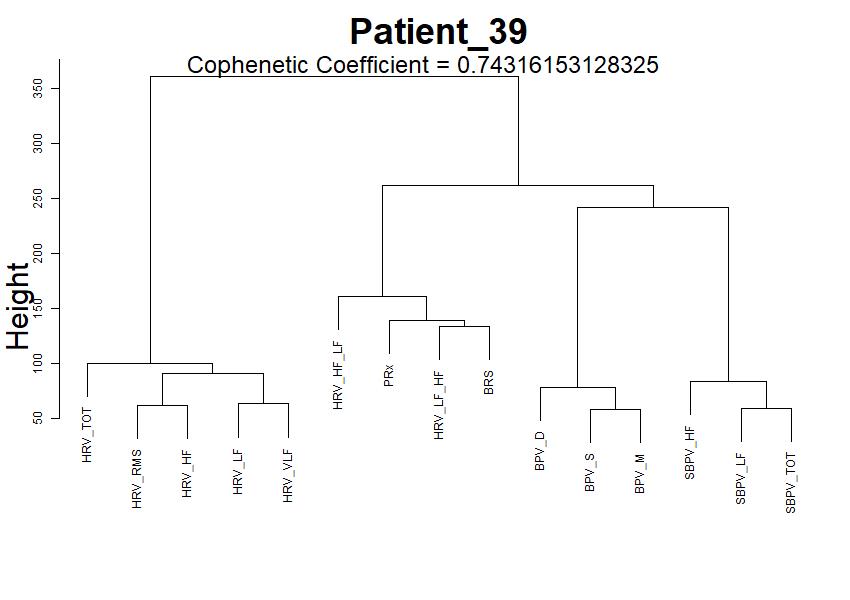

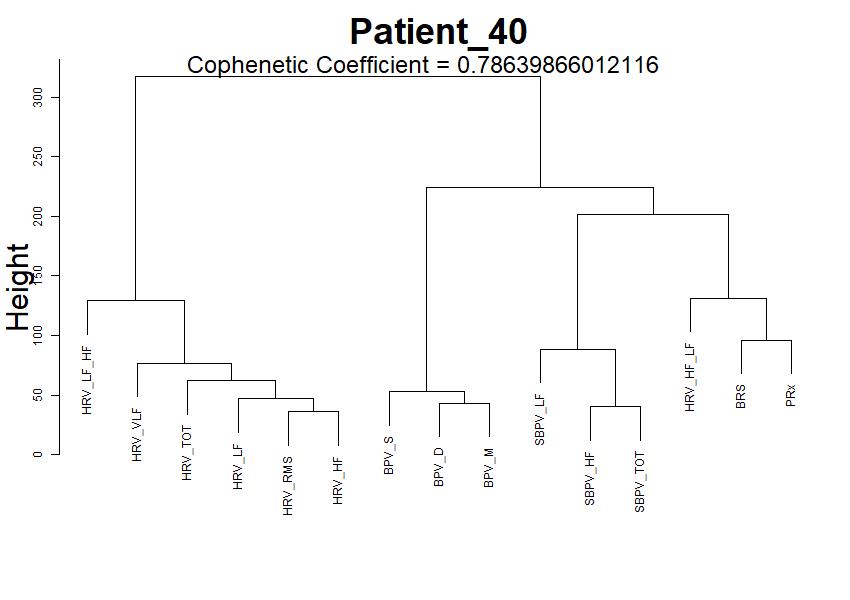

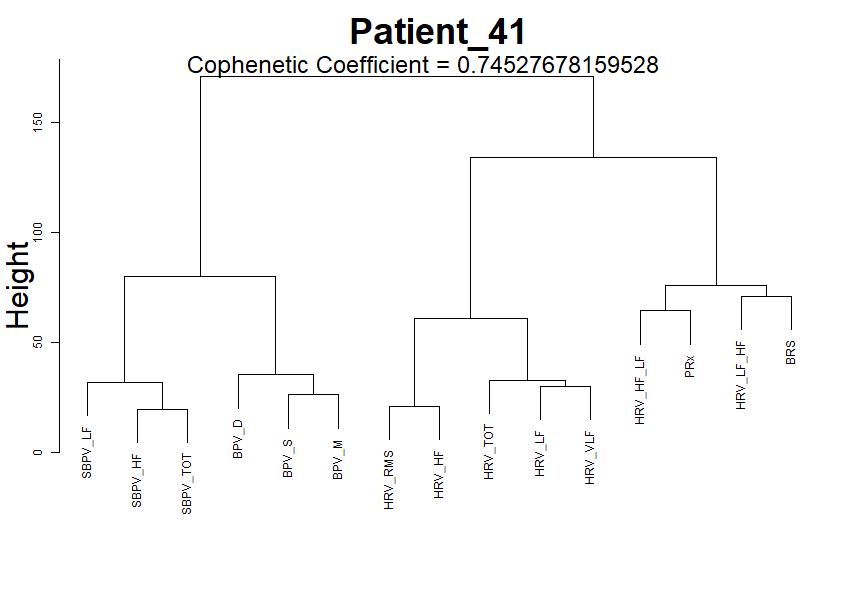

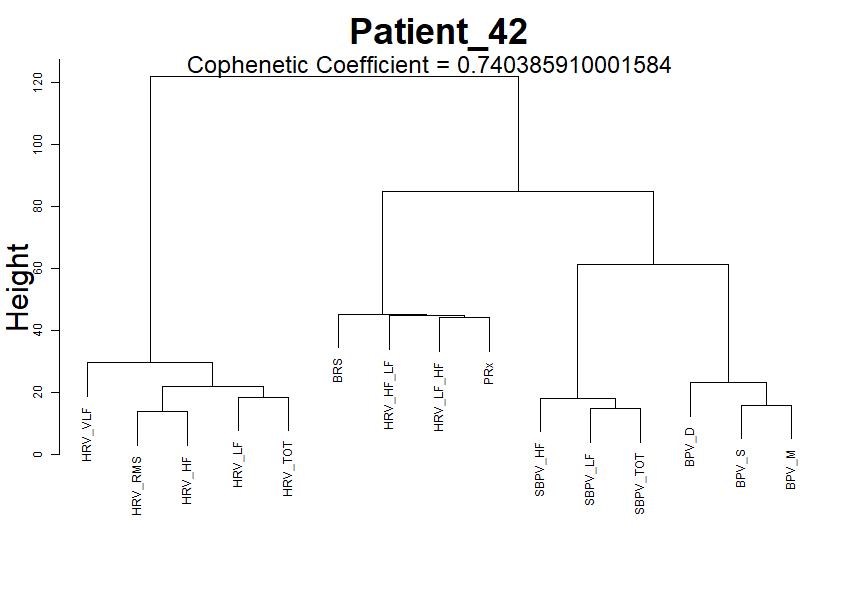

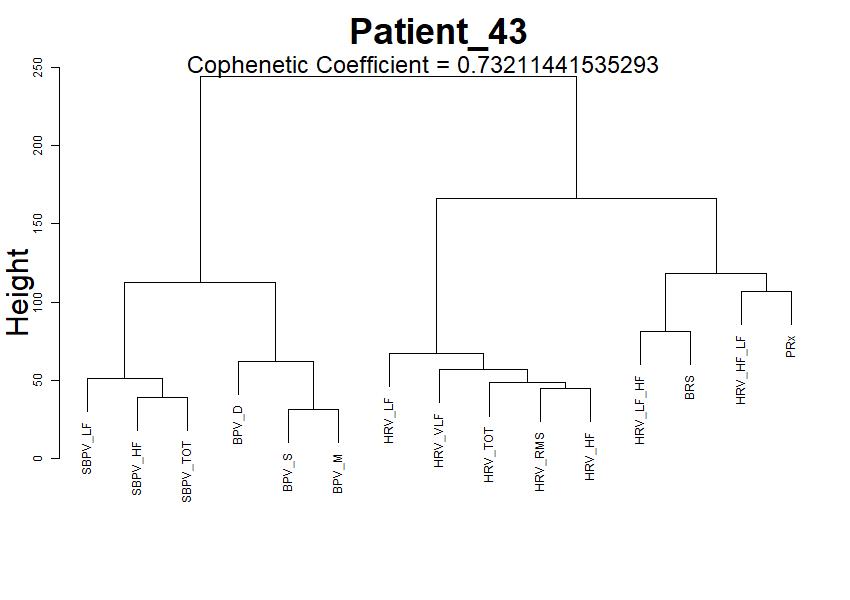

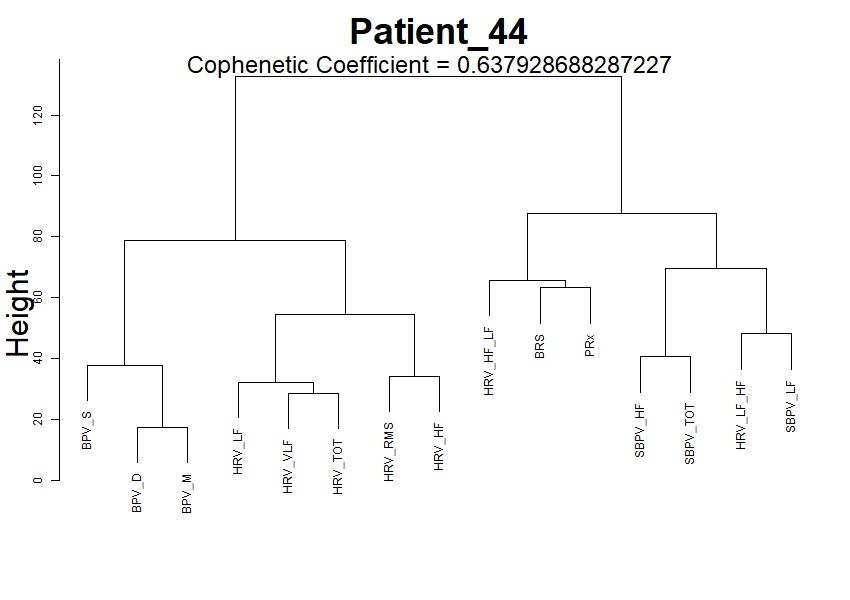

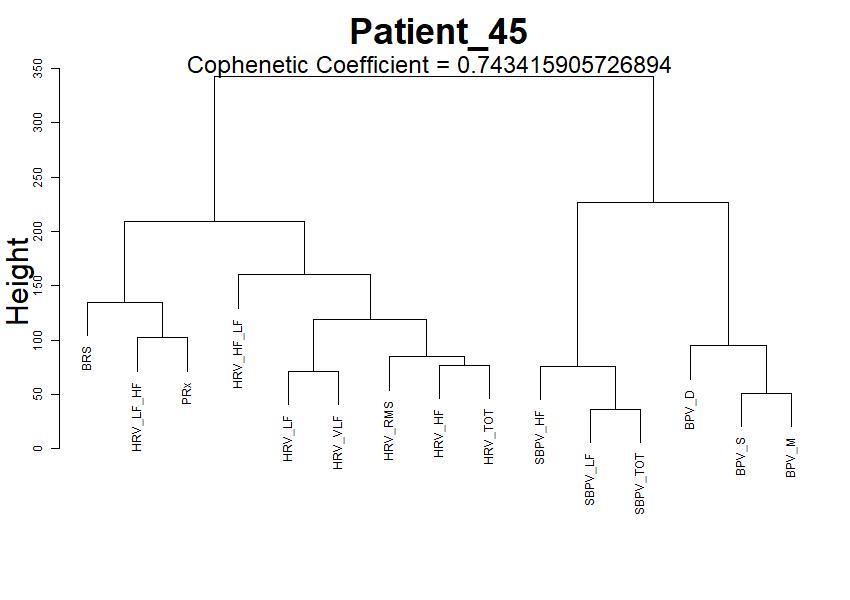

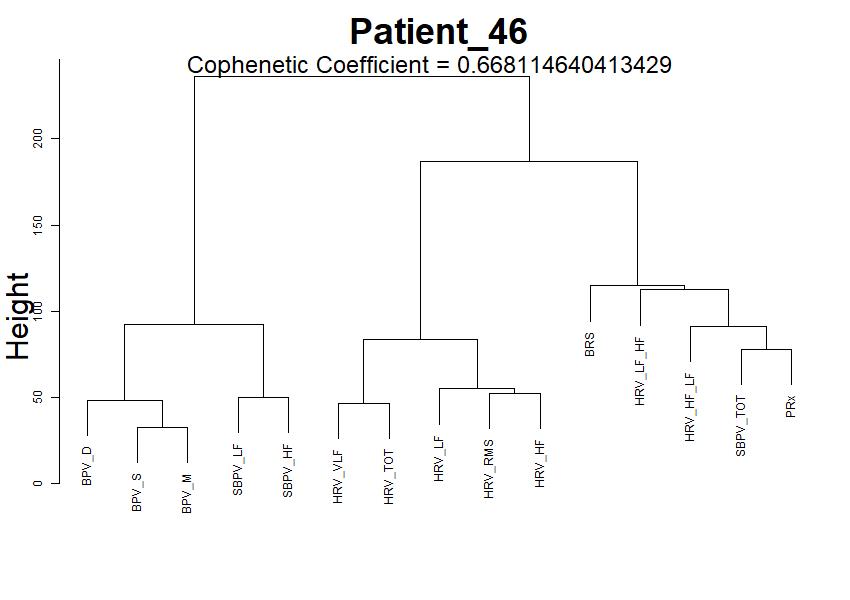

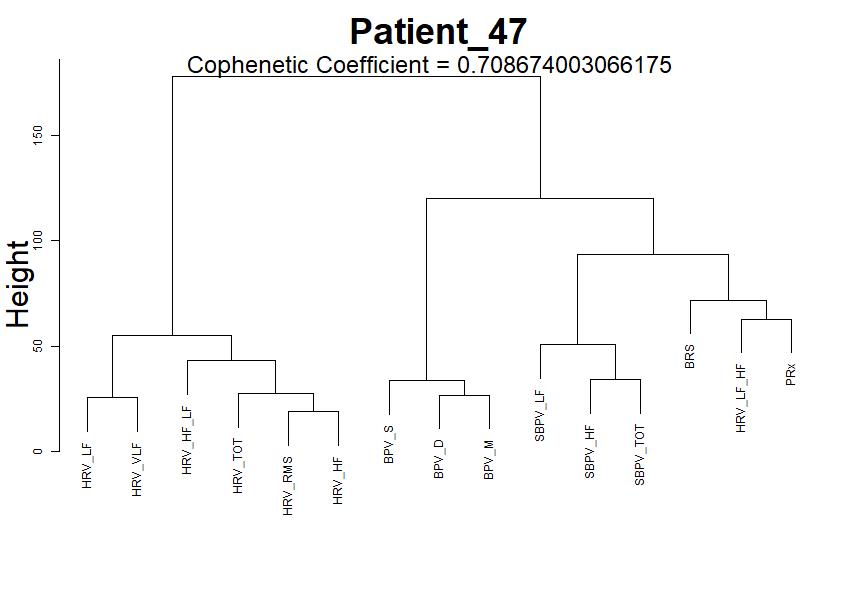

Supplement: Supplementary file 2 [file DataSheet7.DOCX]
