# Supplementary material for: Dynamic Temporal Relationship Between Autonomic Function and Cerebrovascular Reactivity in Moderate/Severe Traumatic Brain Injury
Source: Front Netw Physiol. 2022 Feb 16;2:837860. doi: 10.3389/fnetp.2022.837860 (PMC10013014; doi:10.3389/fnetp.2022.837860)
Supplement: Supplementary file 3 [file DataSheet6.DOCX]

Appendix E. Granger testing for full data PRx and Autonomic variables - each patient

|  | **Granger test: PRx on BPV_S** | | **Granger test: BPV_S on PRx** | | **Granger test: PRx on BPV_D** | | **Granger test: BPV_D on PRx** | |
| --- | --- | --- | --- | --- | --- | --- | --- | --- |
|  | F-test value | P-value | F-test value | P-value | F-test value | P-value | F-test value | P-value |
| Patient 1 | 2.08010875702976 | 0.0806202499423049 | 0.104141431416554 | 0.981095040896511 | 1.19841762830925 | 0.309215728727045 | 0.15996204967099 | 0.958528478990284 |
| Patient 2 | 4.25768689543078 | 0.00198412140322725 | 2.71971926810896 | 0.0283607363041058 | 3.62121719777402 | 0.00606367684302328 | 1.19971120338544 | 0.309079610464951 |
| Patient 3 | 1.5609743189221 | 0.182105624650028 | 1.32475364317655 | 0.258369995691539 | 1.53271302616793 | 0.190030200823334 | 0.211578290276074 | 0.932100357527205 |
| Patient 4 | 1.68883309167911 | 0.151030174521535 | 0.433293421950378 | 0.784609086676873 | 1.15063446200648 | 0.331778401751644 | 2.15960162479268 | 0.0722590903763919 |
| Patient 5 | 3.54312639887805 | 0.00679813432807404 | 1.00209243362139 | 0.404946020500775 | 1.63595343805567 | 0.162172316273429 | 0.552981063831796 | 0.696852455744891 |
| Patient 6 | 2.8237031894446 | 0.0237471996966253 | 0.423316223386941 | 0.791913375466655 | 5.04429569251405 | 0.000483422362825524 | 2.45857257283496 | 0.0437163335554277 |
| Patient 7 | 0.901640521740075 | 0.461914939934208 | 1.81851264156148 | 0.122209788389999 | 0.0223200279403557 | 0.999032567412663 | 0.609089138772216 | 0.656079151038692 |
| Patient 8 | 3.86134205430756 | 0.00395137602562812 | 7.454763413 | 5.87060787923865e-06 | 4.38727488261934 | 0.00155845650623985 | 2.19533124553506 | 0.0672125335711375 |
| Patient 9 | 0.170537794791451 | 0.953427934875242 | 2.2806552495549 | 0.0592979051598982 | 3.52315346525576 | 0.00741275437980183 | 2.51334393786995 | 0.0405670725041108 |
| Patient 10 | 0.99440898587873 | 0.409338827178151 | 1.71499306917288 | 0.143991035708508 | 0.0690784440754444 | 0.991283461883415 | 0.132881261693106 | 0.970338194977227 |
| Patient 11 | 3.53963224176375 | 0.00685319378628906 | 1.36561278043835 | 0.243204487915492 | 2.43633885690135 | 0.0450721409068038 | 0.748927697755553 | 0.558589372222446 |
| Patient 12 | 3.45415644032354 | 0.00813624437452998 | 1.11546179705307 | 0.347633510593333 | 1.14972779274811 | 0.331543247999664 | 1.707506721293 | 0.145905138440914 |
| Patient 13 | 0.319164259828298 | 0.865245611519399 | 0.171696698725998 | 0.952893399097152 | 0.393877510838878 | 0.81312704637753 | 1.01516100398735 | 0.398417846807005 |
| Patient 14 | 2.45007222591327 | 0.0440838295462175 | 1.08644938786882 | 0.361364778638207 | 1.98000950637799 | 0.0947472925471316 | 2.88125720023853 | 0.0213659614701084 |
| Patient 15 | 0.734284831346421 | 0.568473337510875 | 1.6891288946759 | 0.149622193772889 | 2.31039432004631 | 0.0555784753177223 | 1.55536945320121 | 0.183496266717129 |
| Patient 16 | 1.4646147033482 | 0.216333367543574 | 0.828470006668188 | 0.509217175290009 | 0.496723716725839 | 0.73815852514761 | 2.17536412009544 | 0.0749681077614374 |
| Patient 17 | 1.66364963734868 | 0.155987917290067 | 0.995064909648227 | 0.409078989316023 | 0.959195032067353 | 0.428911436164338 | 2.19027441661281 | 0.0679555546457246 |
| Patient 18 | 2.60577145236728 | 0.0339357254229986 | 0.663672142461886 | 0.617181832585864 | 0.803268806159372 | 0.522853170671782 | 1.82865886408798 | 0.120258393852154 |
| Patient 19 | 1.01257159559627 | 0.399332858543717 | 4.05191579794806 | 0.00277568049189386 | 0.417738880035196 | 0.795978525508048 | 6.62526013021785 | 2.56908526316435e-05 |
| Patient 20 | 1.39474626795497 | 0.233005609947011 | 4.42841240183549 | 0.00141728317332282 | 0.842740289358641 | 0.49782392855365 | 2.91139964087907 | 0.0202656214206749 |
| Patient 21 | 2.6892100370773 | 0.0296469299179897 | 0.830717200303732 | 0.50544134074694 | 1.30326727785831 | 0.266414180331084 | 0.57453630239735 | 0.681129148540688 |
| Patient 22 | 2.47412642664846 | 0.0423439660651208 | 1.34232262621088 | 0.251624720872076 | 2.15772790140245 | 0.0711808850421435 | 1.19186764797526 | 0.312143108738702 |
| Patient 23 | 2.90012230575865 | 0.020675144175601 | 2.81776926854926 | 0.0237795573209267 | 5.12185795261246 | 0.000406587029262893 | 2.18346683907926 | 0.0682602406256029 |
| Patient 24 | 4.12641172443592 | 0.00245551571641111 | 1.66641126145142 | 0.154969220416535 | 2.64518059370571 | 0.0319134708154815 | 2.07573165658427 | 0.0813880745466712 |
| Patient 25 | 3.20588060248063 | 0.0123186256253441 | 0.823484989942297 | 0.510029102745086 | 2.47193269564669 | 0.0426388259309521 | 1.37097504855766 | 0.241488052289421 |

|  | **Granger test: PRx on BPV_S** | | **Granger test: BPV_S on PRx** | | **Granger test: PRx on BPV_D** | | **Granger test: BPV_D on PRx** | |
| --- | --- | --- | --- | --- | --- | --- | --- | --- |
|  | F-test value | P-value | F-test value | P-value | F-test value | P-value | F-test value | P-value |
| Patient 26 | 4.85720557711952 | 0.00067853612183432 | 0.389533682981726 | 0.816257997668341 | 1.35395730796608 | 0.247745475911546 | 3.8546829439911 | 0.00401894885582576 |
| Patient 27 | 4.26802859950357 | 0.00190188440081279 | 2.4693108538163 | 0.0427397898708349 | 3.44067670213294 | 0.00817243984277491 | 2.48491483327614 | 0.0416471565240622 |
| Patient 28 | 3.59301374224533 | 0.00637248037305459 | 4.78251340958142 | 0.000781185742974412 | 1.57215013231831 | 0.179262662036047 | 3.29747002149251 | 0.0106244992487018 |
| Patient 29 | 3.89833871693893 | 0.00369245036358308 | 0.620323885914937 | 0.648035967643602 | 2.1068464947466 | 0.0774952781300556 | 3.25834867145907 | 0.0112686429371272 |
| Patient 30 | 0.332280292428686 | 0.856239245721636 | 1.35079875466386 | 0.250299088592739 | 0.697535608473183 | 0.593977494210977 | 1.30203543808546 | 0.268461688524944 |
| Patient 31 | 1.78840394897604 | 0.128170120852199 | 2.33853890521203 | 0.0529784587046983 | 1.40108270627365 | 0.230877890048187 | 1.89593460001451 | 0.108250237710017 |
| Patient 32 | 0.16581499684961 | 0.95573750353071 | 1.81317753259676 | 0.123882485578983 | 2.20374537351438 | 0.0665629091576667 | 1.3136352690564 | 0.262834629223184 |
| Patient 33 | 2.22317402842393 | 0.0643970913647236 | 3.86811985187208 | 0.00393947271663176 | 1.98331833473992 | 0.094671446570414 | 1.69140428240973 | 0.149452044469254 |
| Patient 34 | 1.81873477252985 | 0.122848499038548 | 1.87114650133351 | 0.113179446001876 | 4.75045606717234 | 0.00083607821599997 | 0.321403564379808 | 0.863744318718465 |
| Patient 35 | 0.78118814518283 | 0.537243974072069 | 1.54453394508706 | 0.186468392945913 | 0.65548694947227 | 0.622977321511098 | 1.27702443567269 | 0.276588176969084 |
| Patient 36 | 0.169392817557531 | 0.953990534826978 | 0.403005562727315 | 0.80655167846663 | 1.82462857451609 | 0.122204828393613 | 1.69910143533386 | 0.148332972005808 |
| Patient 37 | 0.431272544846818 | 0.786141780373485 | 2.56426877795278 | 0.036511426930972 | 0.507825740154927 | 0.730003071795796 | 0.986843456678983 | 0.413337282753234 |
| Patient 38 | 2.43807542217555 | 0.0448768544628892 | 2.88577118557596 | 0.0211377468439838 | 1.02003970351339 | 0.395317045645917 | 0.869801818944114 | 0.481082452119792 |
| Patient 39 | 4.66440519528786 | 0.000926485905659419 | 1.49020231282679 | 0.202218672343215 | 1.54710556989126 | 0.185638312850064 | 1.39092548529328 | 0.234305471744496 |
| Patient 40 | 2.81263694263692 | 0.0241219540082382 | 0.342832164275219 | 0.849129668984668 | 0.567545389779772 | 0.686227894472792 | 0.792012958178059 | 0.530239572125457 |
| Patient 41 | 1.54607974538112 | 0.186752701946264 | 2.5620106659573 | 0.0371292190493372 | 1.88039633657461 | 0.111715230060205 | 2.94105426266358 | 0.0196909244056056 |
| Patient 42 | 1.35489207141479 | 0.247101519070012 | 1.61283157298967 | 0.168115576052726 | 1.47873762562408 | 0.205815156798259 | 1.64050577501049 | 0.161157297290359 |
| Patient 43 | 1.48693759577194 | 0.203319389575647 | 0.618747797375353 | 0.649147337554768 | 1.30181897717327 | 0.2668950052363 | 0.842836846264558 | 0.497802917268918 |
| Patient 44 | 2.1905500006152 | 0.0683446226656647 | 0.83637669247109 | 0.502197023749827 | 1.90450554010829 | 0.107748208874777 | 0.306568150167115 | 0.87365573231742 |
| Patient 45 | 0.779680873941069 | 0.538973590541999 | 0.565446871194083 | 0.68788980904896 | 0.848524757863831 | 0.49521117795939 | 2.47006772202177 | 0.04447046552293 |
| Patient 46 | 5.03135959178885 | 0.000481163887228502 | 7.12034544719801 | 1.03901885999361e-05 | 2.70695979994849 | 0.0287068805679378 | 4.79310472920009 | 0.000740021164368631 |
| Patient 47 | 0.936132533860991 | 0.441963495312677 | 0.60493314057893 | 0.659119058837077 | 2.78970501065275 | 0.0251585045832854 | 0.740822547839466 | 0.564131064799909 |

|  | **Granger test: PRx on BPV_M** | | **Granger test: BPV_M on PRx** | | **Granger test: PRx on SBPV_HF** | | **Granger test: SBPV_HF on PRx** | |
| --- | --- | --- | --- | --- | --- | --- | --- | --- |
|  | F-test value | P-value | F-test value | P-value | F-test value | P-value | F-test value | P-value |
| Patient 1 | 2.00497412341037 | 0.0909542619268332 | 0.565105101155213 | 0.687990363119312 | 1.11777731829398 | 0.346064267555986 | 1.10287000421463 | 0.353248765916322 |
| Patient 2 | 3.13921185985273 | 0.0139321239995313 | 3.84347103983868 | 0.00411375691797889 | 2.6922300071477 | 0.0297071495428884 | 0.401747730097698 | 0.807494378275738 |
| Patient 3 | 4.23142754271663 | 0.00205402825154945 | 1.01259286555953 | 0.399503469767224 | 4.30545065154391 | 0.00180769056336618 | 0.496978861632474 | 0.737980513159 |
| Patient 4 | 1.88820595955846 | 0.111004087009288 | 2.99205614154814 | 0.0183907273534595 | 1.57631830602199 | 0.179128652124082 | 0.54798240188612 | 0.700576959756122 |
| Patient 5 | 3.17950624579495 | 0.0127885383779867 | 0.717408490139393 | 0.579901574626518 | 2.02577968328304 | 0.0879883648407874 | 2.20003991886626 | 0.0664003006031218 |
| Patient 6 | 2.48572501244292 | 0.0417960461429005 | 0.318254645387179 | 0.865891210591526 | 2.91048017391487 | 0.0205043399951719 | 0.243350557929576 | 0.913758537153545 |
| Patient 7 | 1.40739854438538 | 0.228677969421772 | 2.3775860704228 | 0.049626137307265 | 2.48224382640301 | 0.0417263847251255 | 3.18435029947937 | 0.0126794218120857 |
| Patient 8 | 4.74470025552605 | 0.00082303856040011 | 3.00490467166949 | 0.0174305547955122 | 2.11659694086187 | 0.0763391604731391 | 11.0646146784564 | 7.09085231162492e-09 |
| Patient 9 | 1.3268507989415 | 0.258441694430732 | 0.552510971251883 | 0.697256374297458 | 0.199537007429394 | 0.938595541149443 | 1.20182462268245 | 0.308794216355397 |
| Patient 10 | 2.30997624886466 | 0.0557990744286381 | 3.04032300549053 | 0.0164312410335534 | 10.8518790610428 | 1.08101308038651e-08 | 1.49516583133456 | 0.201101350933954 |
| Patient 11 | 2.87305358897195 | 0.0216472790595457 | 3.80850723515058 | 0.00427446020521592 | 5.5966027584907 | 0.000171136288236236 | 1.15630548849959 | 0.328084732019643 |
| Patient 12 | 3.23403054942341 | 0.0118844610191236 | 0.963455858250924 | 0.426559744103066 | 0.825150655731669 | 0.50911905430058 | 0.16571733309951 | 0.955783909172898 |
| Patient 13 | 0.887655328378346 | 0.470636806843551 | 0.55284828188461 | 0.696986858868786 | 1.34571441317964 | 0.251057829217234 | 1.25171920735774 | 0.287370277385751 |
| Patient 14 | 2.38711309149186 | 0.0489218341400394 | 1.67258162810254 | 0.153390940602334 | 7.00461597437696 | 1.2872332603299e-05 | 2.68720313315447 | 0.0296757350910287 |
| Patient 15 | 0.691323053006249 | 0.597896869308445 | 2.16807274679039 | 0.0700940452190577 | 3.53199632293974 | 0.00698027252214141 | 0.354255391283361 | 0.84120832053937 |
| Patient 16 | 1.62666502573194 | 0.171010633980135 | 0.242657262541205 | 0.913677018417057 | 2.03862405357742 | 0.0928664969484511 | 0.257176127112059 | 0.904814505553994 |
| Patient 17 | 1.09738923992562 | 0.356317118078108 | 1.05877733368273 | 0.375565658272893 | 0.726456722890428 | 0.573872690643305 | 2.80119584039163 | 0.0247532181821881 |
| Patient 18 | 1.00926511360571 | 0.401068811946145 | 0.0626231367759754 | 0.992780610168966 | 3.80328185504285 | 0.00429809587638007 | 1.55570748092534 | 0.183205156247152 |
| Patient 19 | 0.789163551763078 | 0.532008434370334 | 5.97247411572975 | 8.56900429863002e-05 | 1.18154118956057 | 0.316681791345174 | 0.866989762958984 | 0.482830075941875 |
| Patient 20 | 1.44972226243925 | 0.214815062808439 | 3.37170106427055 | 0.00917424883924084 | 1.14013097775048 | 0.335536663369276 | 2.97068440950149 | 0.0183160582568012 |
| Patient 21 | 1.47758932711176 | 0.206262271052987 | 0.281564190695994 | 0.890055937347773 | 1.96148815790268 | 0.0977077437210496 | 3.21989975672434 | 0.0120095122099931 |
| Patient 22 | 3.31696083065335 | 0.010103472369975 | 1.14607761177673 | 0.332803465570901 | 3.31302620912713 | 0.0101727722698512 | 3.88708247642156 | 0.0037235547455516 |
| Patient 23 | 1.72975307390171 | 0.140404617420087 | 3.89048634708593 | 0.00369932986702184 | 2.94887687010246 | 0.0190270803258216 | 4.16713362073529 | 0.00226479549669936 |
| Patient 24 | 3.3751269726867 | 0.00917915557471766 | 1.09104823041327 | 0.359176184376239 | 2.4245207474196 | 0.0460799322869346 | 3.94949276338783 | 0.00335858558360379 |
| Patient 25 | 1.80404107432653 | 0.125266987438645 | 1.37090162339378 | 0.241513987353112 | 0.119101759092894 | 0.975742818236854 | 0.489524375010238 | 0.743462784185775 |

|  | **Granger test: PRx on BPV_M** | | **Granger test: BPV_M on PRx** | | **Granger test: PRx on SBPV_HF** | | **Granger test: SBPV_HF on PRx** | |
| --- | --- | --- | --- | --- | --- | --- | --- | --- |
|  | F-test value | P-value | F-test value | P-value | F-test value | P-value | F-test value | P-value |
| Patient 26 | 3.53235336389445 | 0.00705475128839567 | 1.39777382327936 | 0.232368766417435 | 0.382980363390256 | 0.820932728672175 | 1.09054708685795 | 0.359596309043504 |
| Patient 27 | 5.08673162687787 | 0.000436716455426737 | 3.70314032625838 | 0.0051661240746906 | 2.12382805740581 | 0.0752654821104906 | 0.143991291834438 | 0.965684218935672 |
| Patient 28 | 3.95361683641651 | 0.00339377428001314 | 4.5484044249344 | 0.00118579009035947 | 2.30258288995682 | 0.0566060524129026 | 7.47719991582682 | 5.87385405480805e-06 |
| Patient 29 | 2.44722635312898 | 0.0444492856550876 | 1.43454044390012 | 0.219963372383017 | 3.92917884375758 | 0.0034975596036365 | 1.07483074042205 | 0.367286852389524 |
| Patient 30 | 0.285838911546134 | 0.887150647556761 | 0.478066010599655 | 0.751856620473982 | 0.410507775715185 | 0.801092747621683 | 0.338244779068922 | 0.852145699083511 |
| Patient 31 | 2.73127145837867 | 0.0275296763603775 | 2.56083784280102 | 0.0366483909030475 | 2.03180133921173 | 0.0871993774555144 | 0.988476513299434 | 0.412384083159893 |
| Patient 32 | 2.61598777051188 | 0.033811218067168 | 1.50073488920343 | 0.199670895567067 | 0.176702475304618 | 0.950438651273447 | 2.77909911266454 | 0.0257458581677445 |
| Patient 33 | 2.62830802808823 | 0.033044860661938 | 1.83386875555891 | 0.119827464722654 | 0.592365164419964 | 0.668209151674506 | 2.66859933708099 | 0.0308982093605969 |
| Patient 34 | 5.41093985031817 | 0.000256439000193425 | 0.732487593229315 | 0.569815425104981 | 3.18932021332698 | 0.0128502248516848 | 2.90494928425931 | 0.0208399631849985 |
| Patient 35 | 0.460262050826758 | 0.764957483211255 | 1.15805214026074 | 0.327337233845813 | 1.81562566628948 | 0.122887930432621 | 3.21155493635827 | 0.0121465825152789 |
| Patient 36 | 1.44725386512177 | 0.21668810721056 | 1.63426289125989 | 0.163752288228433 | 1.59243973975319 | 0.174479476061997 | 1.55667606913496 | 0.184131742328505 |
| Patient 37 | 0.632768121561888 | 0.639117545997112 | 0.885192429876357 | 0.471833060704112 | 3.2155294591069 | 0.0120825664776967 | 3.79970167640743 | 0.00436587543660875 |
| Patient 38 | 2.74529859758965 | 0.0268266358519169 | 2.85572123294482 | 0.0222466992158212 | 2.16465856110163 | 0.0702964216893693 | 1.68246391688531 | 0.150955386239207 |
| Patient 39 | 1.78407125508432 | 0.128987585200231 | 0.502540428364948 | 0.733890011759257 | 1.03830595555009 | 0.385747646146271 | 0.397774594771297 | 0.810382971647543 |
| Patient 40 | 1.98087712902017 | 0.0948123105748225 | 0.735470596487118 | 0.56770148212089 | 3.41486509321722 | 0.00859764540042265 | 4.60792996291941 | 0.0010474368691857 |
| Patient 41 | 1.08932717779894 | 0.36046822788725 | 2.09720184252231 | 0.0792208267869983 | 0.352952577123438 | 0.842062723630097 | 1.07863628039934 | 0.365770499321484 |
| Patient 42 | 2.16705722271946 | 0.0701563516599876 | 2.76989724000775 | 0.0258223337806541 | 11.6537730991729 | 2.07844998206544e-09 | 3.5300139657324 | 0.00698734763987059 |
| Patient 43 | 0.817876740762701 | 0.513546646215083 | 1.26233701866057 | 0.282476415562317 | 2.01807440785758 | 0.0891966719447018 | 4.19666120748418 | 0.0021577439872279 |
| Patient 44 | 1.62416937093163 | 0.166153298754555 | 0.340550720349815 | 0.850643313383057 | 5.73152638259296 | 0.000151132576692027 | 0.34117093307146 | 0.850210723959828 |
| Patient 45 | 0.874579656419367 | 0.479270524454879 | 0.791611981057082 | 0.531223427861881 | 4.4977109909186 | 0.00148668050377065 | 2.02260374038939 | 0.0908589171824957 |
| Patient 46 | 4.83756004652508 | 0.000683002622025315 | 7.95418642193914 | 2.19944503228366e-06 | 2.40533960717005 | 0.0474868150324594 | 5.20301605250932 | 0.000352561389194185 |
| Patient 47 | 0.871571618186621 | 0.480202770414749 | 1.61149421653931 | 0.168776150884363 | 1.99489413087376 | 0.092860377740711 | 3.47511079449758 | 0.00778675799677654 |

|  | **Granger test: PRx on SBPV_LF** | | **Granger test: SBPV_LF on PRx** | | **Granger test: PRx on SBPV_TOT** | | **Granger test: SBPV_TOT on PRx** | |
| --- | --- | --- | --- | --- | --- | --- | --- | --- |
|  | F-test value | P-value | F-test value | P-value | F-test value | P-value | F-test value | P-value |
| Patient 1 | 1.09123490290523 | 0.358938224602158 | 0.713556532225784 | 0.582533507008977 | 2.82969285236283 | 0.0232665687867485 | 0.840248845792888 | 0.499368336412329 |
| Patient 2 | 1.3851589516605 | 0.236791077781479 | 1.78187396623641 | 0.129964113833446 | 2.53180601957291 | 0.038806959304147 | 1.83810697271831 | 0.119045632295141 |
| Patient 3 | 2.12215965823492 | 0.0756964724183731 | 0.0690057600405225 | 0.99130100879646 | 1.96217186167321 | 0.0977732477969947 | 0.502112085029205 | 0.734205802711639 |
| Patient 4 | 2.2995176328885 | 0.0577205613021712 | 2.89460588648921 | 0.0216717396788767 | 1.84448386595259 | 0.118863955921818 | 1.3168756979062 | 0.262400428344006 |
| Patient 5 | 2.41673491567672 | 0.0465221239392927 | 1.48071640312816 | 0.205101047985423 | 2.14682464823792 | 0.0723945525657981 | 0.901581941421003 | 0.461953122952092 |
| Patient 6 | 2.21750018227945 | 0.0648973813000342 | 0.169160836901927 | 0.954146386974755 | 2.28468861442001 | 0.0581719312458715 | 0.311485177319884 | 0.870432874281209 |
| Patient 7 | 1.32144974370392 | 0.259311154644979 | 0.990201338416469 | 0.411402474889723 | 2.16730213562831 | 0.0700234701324028 | 2.17518605018181 | 0.0691326641937443 |
| Patient 8 | 3.57035900163905 | 0.00657543825043887 | 6.59581366784939 | 2.85520488663552e-05 | 3.65590153996995 | 0.00566400060333708 | 10.1577437804758 | 3.86454689416044e-08 |
| Patient 9 | 1.00384835041377 | 0.404780260914082 | 3.34760629203542 | 0.0100188196969829 | 0.623971800751244 | 0.64554631165138 | 0.900025800628424 | 0.463492269671164 |
| Patient 10 | 3.54772168577344 | 0.00685436938298583 | 1.75373202473645 | 0.135622759723419 | 8.65453382249311 | 6.45625499267156e-07 | 2.00008670338125 | 0.0920613620459231 |
| Patient 11 | 4.03475593553947 | 0.00286483488824873 | 0.840003706483671 | 0.499545934744329 | 3.0926178247909 | 0.0148743080780811 | 0.56269897346002 | 0.689750667175878 |
| Patient 12 | 1.09640686686424 | 0.356856477211756 | 0.941507890964844 | 0.438990290663603 | 1.08601203873982 | 0.361964637894062 | 0.0327630767330505 | 0.997941556304111 |
| Patient 13 | 1.37268330833384 | 0.241397683759689 | 0.294913281397753 | 0.881357243589359 | 1.21653872284145 | 0.302059800232608 | 0.347278897523149 | 0.846010328061133 |
| Patient 14 | 2.66714544107796 | 0.0306934399375669 | 0.285738265684989 | 0.887379990620701 | 3.57532730630432 | 0.00645156548050314 | 0.787425362195562 | 0.533163057739567 |
| Patient 15 | 2.05053612823701 | 0.084722822744819 | 1.14273672479974 | 0.334435157163509 | 3.0375474599172 | 0.0164060825856283 | 0.915025208482286 | 0.454097568228931 |
| Patient 16 | 1.49845262297404 | 0.206554543786596 | 0.297275645794407 | 0.879275049476917 | 1.92493057917956 | 0.110233236125371 | 0.297696791332696 | 0.878998702767731 |
| Patient 17 | 1.20287619994692 | 0.307735048316063 | 1.7143170774412 | 0.144313000967209 | 1.37956200372488 | 0.238759391778407 | 3.27358335192462 | 0.0110785312199227 |
| Patient 18 | 0.283401200163216 | 0.888896508085733 | 2.68369769827916 | 0.0297709536887863 | 0.404649407414527 | 0.805440158566623 | 2.29049529141995 | 0.0572531287098756 |
| Patient 19 | 3.44735173123341 | 0.00804449175073801 | 1.08374585068872 | 0.362666655683582 | 0.928702167298631 | 0.446050554745543 | 1.13924239987889 | 0.335955864052946 |
| Patient 20 | 32.4029312332742 | 8.79231399072522e-27 | 17.2449787225495 | 4.42978025643173e-14 | 23.1710960087212 | 4.85647873906959e-19 | 20.2892242142897 | 1.26057988109229e-16 |
| Patient 21 | 0.526372853034541 | 0.716370954550424 | 1.51586487004891 | 0.194783521888765 | 2.47814573469492 | 0.0421730354673627 | 2.10255519755011 | 0.0779606305783693 |
| Patient 22 | 1.44766244200722 | 0.21551262551047 | 1.3261514908451 | 0.257609234245833 | 2.80457359851273 | 0.0243231247829016 | 1.87024945303438 | 0.112731469491089 |
| Patient 23 | 1.14893245935496 | 0.331477050396362 | 2.96058312338297 | 0.0186506835867357 | 0.584985112885714 | 0.673520738730897 | 1.09335370588866 | 0.357942668551039 |
| Patient 24 | 1.83347539420799 | 0.119580126281833 | 2.86624585836186 | 0.0219824850596706 | 1.73751812593935 | 0.138873000937664 | 3.93148053168498 | 0.00346709014444416 |
| Patient 25 | 0.191343939893226 | 0.943003527087661 | 0.456291055441835 | 0.767863404420998 | 0.223771168778187 | 0.925236039782026 | 0.477483700442599 | 0.752313284797354 |

|  | **Granger test: PRx on SBPV_LF** | | **Granger test: SBPV_LF on PRx** | | **Granger test: PRx on SBPV_TOT** | | **Granger test: SBPV_TOT on PRx** | |
| --- | --- | --- | --- | --- | --- | --- | --- | --- |
|  | F-test value | P-value | F-test value | P-value | F-test value | P-value | F-test value | P-value |
| Patient 26 | 0.297231004745913 | 0.879869716414413 | 2.33661197656178 | 0.0534657317654238 | 0.379393397323813 | 0.823483682125389 | 1.89805049313912 | 0.108273614431472 |
| Patient 27 | 9.48314022099967 | 1.2666876129373e-07 | 5.09521742102692 | 0.000430122764511222 | 1.42783271072496 | 0.222013477373996 | 1.79819154381391 | 0.126304741242974 |
| Patient 28 | 2.56357324451654 | 0.036822237036549 | 6.57255692089147 | 3.07498570229629e-05 | 3.04130478385997 | 0.016476069288614 | 5.62633129190907 | 0.000171488212729012 |
| Patient 29 | 5.11820014077787 | 0.000418467073136571 | 2.62680293001029 | 0.0329729326137142 | 4.90106476850586 | 0.000618918506081495 | 3.02079433569964 | 0.016939689050384 |
| Patient 30 | 0.583311713915555 | 0.674891763021498 | 0.316137427303593 | 0.867174781594772 | 0.301021403908404 | 0.877237274423525 | 0.488786047488163 | 0.743990407199825 |
| Patient 31 | 6.39354394602463 | 3.96307296582368e-05 | 3.89414063874423 | 0.00367703072031202 | 2.79622415915144 | 0.0246682782052382 | 2.34501310473725 | 0.0524180523310518 |
| Patient 32 | 0.891278417420968 | 0.468399404345679 | 0.934905942531696 | 0.442784094074652 | 0.419888553479205 | 0.79438713293193 | 2.39010155384851 | 0.0491329656905027 |
| Patient 33 | 1.46253415267682 | 0.211246921776066 | 2.92747909308033 | 0.0199819227543087 | 1.19918406880738 | 0.309312255460976 | 2.15906963273466 | 0.0714448556876737 |
| Patient 34 | 0.939242971071061 | 0.440306330699588 | 2.15805868984776 | 0.0716976813904284 | 1.98545556585916 | 0.09450221286246 | 4.35098752467446 | 0.0016988849709727 |
| Patient 35 | 1.58934218171996 | 0.174239889666685 | 1.49330879661071 | 0.201387962649146 | 2.81617425072929 | 0.0238815097362199 | 1.32849062613275 | 0.256785579748773 |
| Patient 36 | 0.249252251683364 | 0.91014216337001 | 0.881511316862098 | 0.474503921687676 | 2.07364348962833 | 0.082575349609936 | 1.27388110822839 | 0.278746022385417 |
| Patient 37 | 3.66252035534083 | 0.00555475048307561 | 4.66117711157059 | 0.000943153166628444 | 2.86530089678548 | 0.0219990033392984 | 5.56627314492257 | 0.000183272856796256 |
| Patient 38 | 3.61999737712507 | 0.00593193920922084 | 1.11289181150473 | 0.348388407801195 | 2.70039339260598 | 0.0289394011084468 | 0.470551983807445 | 0.757410778564947 |
| Patient 39 | 2.58931165389861 | 0.0349096253062673 | 2.7251773776623 | 0.0277823322077217 | 1.03834363914426 | 0.385728033015289 | 0.657466358749068 | 0.621562266413209 |
| Patient 40 | 3.42096632804749 | 0.00850714961167461 | 1.52036635573297 | 0.193551247353086 | 4.72286779850597 | 0.000852511217820289 | 4.79770410863882 | 0.00074536793125897 |
| Patient 41 | 0.866644718388623 | 0.483392938147721 | 0.813823790344266 | 0.516394318092609 | 1.68376571356628 | 0.151555073611303 | 0.596547962948642 | 0.665213015043619 |
| Patient 42 | 14.0526761416991 | 2.1778234902882e-11 | 2.00469734782272 | 0.0911143362793919 | 16.3767315748862 | 2.59012466090913e-13 | 3.97349897122121 | 0.00320414640240473 |
| Patient 43 | 4.33514853876848 | 0.00168581569265953 | 3.24739661885923 | 0.0114196623526623 | 1.3537587310663 | 0.247520414410427 | 9.71569607688802 | 8.1326904985396e-08 |
| Patient 44 | 1.59224840326627 | 0.17444440084582 | 0.221381900629387 | 0.926535219424496 | 1.28299814412008 | 0.27506545985252 | 3.77705947475995 | 0.00474497893975581 |
| Patient 45 | 2.51806096383027 | 0.041175060823591 | 0.59544780353532 | 0.666160277701818 | 2.38473689344765 | 0.05107812037506 | 0.643893593965437 | 0.631539166906658 |
| Patient 46 | 5.80555346050402 | 0.000117497226849511 | 4.92658220052167 | 0.00058170637927415 | 3.17478388171011 | 0.0129350164419903 | 4.19153202139521 | 0.00217490361511564 |
| Patient 47 | 0.402829901299065 | 0.806722268563083 | 9.2857884002446 | 2.02316610539097e-07 | 1.17196536120808 | 0.321276296804978 | 8.15673743514868 | 1.63835051703882e-06 |

|  | **Granger test: PRx on BRS** | | **Granger test: BRS on PRx** | | **Granger test: PRx on HRV_HF** | | **Granger test: HRV_HF on PRx** | |
| --- | --- | --- | --- | --- | --- | --- | --- | --- |
|  | F-test value | P-value | F-test value | P-value | F-test value | P-value | F-test value | P-value |
| Patient 1 | 0.430250613416178 | 0.786893234208456 | 0.2006793430662 | 0.938074781276033 | 0.795934700276619 | 0.527597273804172 | 0.976395509714304 | 0.418995273714192 |
| Patient 2 | 1.54127561038879 | 0.187832993768523 | 2.92593548711975 | 0.0200554066377128 | 2.84598553461054 | 0.0229394948267849 | 1.12774996047541 | 0.341723690971821 |
| Patient 3 | 0.872644749692195 | 0.479558037753791 | 0.930932874524158 | 0.44497155454356 | 3.2141061827937 | 0.012200576535642 | 1.96961269287539 | 0.0966253624191326 |
| Patient 4 | 0.533196401339724 | 0.711403927907734 | 0.514720910171427 | 0.72495206233516 | 0.715590904896902 | 0.581497234994632 | 0.833480612245076 | 0.50420646727575 |
| Patient 5 | 0.286693499210373 | 0.886769982356427 | 1.04728733264132 | 0.381100104217962 | 0.689268414419036 | 0.59928949862245 | 2.32007839519434 | 0.0545645423343253 |
| Patient 6 | 7.22526203893359 | 9.3187857588757e-06 | 0.63769517575051 | 0.635656688066726 | 2.76818631768284 | 0.0260832785728019 | 0.503205836908708 | 0.733401621990867 |
| Patient 7 | 4.38264765823552 | 0.00153618936314091 | 4.81847359617886 | 0.000701192312864478 | 12.3279497988432 | 5.38965315087217e-10 | 1.05480600281948 | 0.37722416891266 |
| Patient 8 | 1.59827376028343 | 0.172139963146511 | 2.02642311349652 | 0.0882455588207124 | 1.85791730092992 | 0.115224025860514 | 0.650372102244085 | 0.626629903015312 |
| Patient 9 | 0.0820458102159967 | 0.987890105165292 | 0.115229726434336 | 0.977136496326957 | 0.114653365785807 | 0.977351779849336 | 0.934329132285034 | 0.443462558580932 |
| Patient 10 | 0.19844553718331 | 0.939240755301701 | 0.553236226001491 | 0.696686372084934 | 1.40531705752931 | 0.22975967571058 | 2.89041025322434 | 0.021206140145772 |
| Patient 11 | 1.18961383832731 | 0.313131467124511 | 0.553924850043124 | 0.696164584441732 | 2.10300074711176 | 0.0777578817096218 | 0.961170448100531 | 0.427526920094632 |
| Patient 12 | 0.724673682633651 | 0.575117866427732 | 1.89477312420176 | 0.109083826715395 | 3.36890397531223 | 0.00942960108232617 | 2.05382150556109 | 0.0847198303695934 |
| Patient 13 | 1.13559571186439 | 0.338278975708296 | 4.19611899929558 | 0.00225779977802242 | 2.00275412281318 | 0.0920645918499598 | 0.971741351049551 | 0.422035532524573 |
| Patient 14 | 4.5359266386711 | 0.00117703227680804 | 4.65907113038167 | 0.000943581761448368 | 0.996602142643661 | 0.407976357694592 | 0.285321991680502 | 0.887648489825898 |
| Patient 15 | 0.718310150570857 | 0.57932774588256 | 0.964325483376569 | 0.425827086673753 | 1.78034504903614 | 0.12991148353529 | 0.338734398474475 | 0.851963558543438 |
| Patient 16 | 0.403504739132819 | 0.80580677574226 | 0.387926768131237 | 0.81692981417285 | 0.161711758867269 | 0.957317780432256 | 0.407107681026023 | 0.803253919754875 |
| Patient 17 | 0.561941208584479 | 0.690342677863223 | 1.87553196208078 | 0.11247157237371 | 1.62203647891476 | 0.166222769688601 | 0.360306531630836 | 0.836946616166954 |
| Patient 18 | 0.678126662689269 | 0.607045849656598 | 2.01556737590512 | 0.0894166650567768 | 0.653553545201578 | 0.624321476975748 | 2.46657455961052 | 0.0428103632660443 |
| Patient 19 | 3.43733377833039 | 0.00819369764898356 | 0.506253927324706 | 0.731158500300204 | 4.51761707016756 | 0.00120830055583641 | 3.52796692594665 | 0.00698871896619526 |
| Patient 20 | 0.262863932150039 | 0.901883351168363 | 0.635847177634812 | 0.636903390885324 | 2.66402055038428 | 0.0308061230282476 | 2.60873597252198 | 0.0338015976724123 |
| Patient 21 | 3.56315899371731 | 0.0066310434429247 | 0.583708730586068 | 0.674462567977268 | 2.74627605821196 | 0.026934092988291 | 6.60269564909315 | 2.75944381411118e-05 |
| Patient 22 | 0.188100312866708 | 0.944700203043037 | 0.950130114957023 | 0.433777731496456 | 4.16213164178061 | 0.00228629312087355 | 2.51495765801216 | 0.0395646902719352 |
| Patient 23 | 0.466297588483942 | 0.760531689727347 | 0.0759600640347217 | 0.989561555581799 | 2.00303008516093 | 0.0913013704717096 | 0.586333898409128 | 0.67254160342842 |
| Patient 24 | 0.395389300522952 | 0.812078608919135 | 0.0906440705838732 | 0.985415376309727 | 2.64585302236712 | 0.0318784037861752 | 5.19221610685748 | 0.000362921413114104 |
| Patient 25 | 0.649197852190057 | 0.62745292603165 | 0.158195337298368 | 0.959329928243486 | 1.49873561641157 | 0.199893267434962 | 0.724167850007393 | 0.575353978111043 |

|  | **Granger test: PRx on BRS** | | **Granger test: BRS on PRx** | | **Granger test: PRx on HRV_HF** | | **Granger test: HRV_HF on PRx** | |
| --- | --- | --- | --- | --- | --- | --- | --- | --- |
|  | F-test value | P-value | F-test value | P-value | F-test value | P-value | F-test value | P-value |
| Patient 26 | 2.00081414494691 | 0.0920626687545214 | 1.47613830178463 | 0.20700045113628 | 1.11094106072931 | 0.349673640350702 | 0.306022658296272 | 0.874067626707437 |
| Patient 27 | 0.551586543730519 | 0.697879709961377 | 0.557283419821913 | 0.693712770457452 | 0.874819874848912 | 0.478100923746112 | 2.02089006536828 | 0.0888121444799486 |
| Patient 28 | 1.23741711585078 | 0.293086209631521 | 0.552838448675148 | 0.69698418642345 | 1.96758019419013 | 0.0970907596086517 | 3.21618954327363 | 0.0122197888146946 |
| Patient 29 | 1.33427300189709 | 0.254805061931574 | 2.23832737348276 | 0.0626432970648514 | 0.0640189835995015 | 0.992464703837874 | 0.741510457122759 | 0.563632114505764 |
| Patient 30 | 1.52210135373195 | 0.194898778500476 | 0.420972616258283 | 0.793521553382124 | 1.5613283095379 | 0.183753484664707 | 1.56493466504121 | 0.182765775295654 |
| Patient 31 | 0.208896901624957 | 0.933600452286203 | 1.06318613293741 | 0.373006237640643 | 4.97968857382579 | 0.000526506358839917 | 1.76802872446896 | 0.132307951445744 |
| Patient 32 | 2.18382523579677 | 0.0687735925612961 | 0.820518253816074 | 0.51206736069599 | 1.96561538197898 | 0.0974977282746935 | 0.924876075502711 | 0.448583304093941 |
| Patient 33 | 2.3508823103324 | 0.0523764506896557 | 2.38073039148823 | 0.0498760689339078 | 1.43716766949602 | 0.21934078617121 | 0.583368213021279 | 0.674731883118474 |
| Patient 34 | 0.36842369488129 | 0.831225314863768 | 1.19344428016869 | 0.312000860888407 | 1.18072110656513 | 0.317553613050244 | 1.43414717459352 | 0.220462290382223 |
| Patient 35 | 0.816622872708279 | 0.514360461985147 | 1.45035547819556 | 0.214747954357858 | 1.9653242848613 | 0.0970238814376997 | 2.64131130075979 | 0.0320676487142322 |
| Patient 36 | 1.73889388208072 | 0.139717265189644 | 0.520578200164674 | 0.720647695820843 | 1.5408302698457 | 0.188560079917247 | 1.53343404217549 | 0.190659618733498 |
| Patient 37 | 0.849183794651458 | 0.493874553372949 | 0.521027539559721 | 0.720297490578365 | 3.62845737058466 | 0.00589628747434014 | 1.28073004973235 | 0.275163016272239 |
| Patient 38 | 4.41407047165029 | 0.00144916785982554 | 0.634375335292165 | 0.637945438668934 | 0.965361352229081 | 0.425127665485972 | 0.852077532377378 | 0.491990122653941 |
| Patient 39 | 2.97499560767107 | 0.0181778160610398 | 1.75437716231918 | 0.135101574013504 | 6.58370593839459 | 2.76597111853515e-05 | 5.40989991071292 | 0.000239646779929083 |
| Patient 40 | 0.726056747550962 | 0.574084131933093 | 0.212762200497235 | 0.931444036126345 | 3.66386529150023 | 0.00557263824962028 | 2.46494215166078 | 0.0431676770868372 |
| Patient 41 | 0.470831163832289 | 0.757185462391117 | 1.05122516227183 | 0.379641495467214 | 0.768105970547395 | 0.546069618250199 | 0.453528805407023 | 0.769866124837583 |
| Patient 42 | 4.48267753660659 | 0.00129463712691534 | 2.17228212062119 | 0.069571105819448 | 2.17179091939673 | 0.0696211246687874 | 4.45663161638498 | 0.00135587590218656 |
| Patient 43 | 1.37887613238603 | 0.238603769495811 | 1.22740680321408 | 0.296904204071599 | 2.76536391061694 | 0.026027438809761 | 2.20674716802955 | 0.0657856285576676 |
| Patient 44 | 0.902279580833244 | 0.462084832869779 | 1.59297579683367 | 0.174325654974912 | 0.795143674481391 | 0.528478584850568 | 1.54299953415138 | 0.187871631037551 |
| Patient 45 | 0.911457871616892 | 0.457456516162543 | 1.24071134584243 | 0.293551531869001 | 4.48878861159651 | 0.00150955210716505 | 4.67184428201869 | 0.00110328928739447 |
| Patient 46 | 0.296413569495607 | 0.880434002585207 | 0.542411724730307 | 0.704598853824766 | 3.34039043726271 | 0.00971561651774032 | 10.662672468101 | 1.35175612246599e-08 |
| Patient 47 | 2.75785822189185 | 0.0265570997735727 | 1.2852831510361 | 0.273613241838297 | 3.65985051132215 | 0.00564681127942212 | 3.55845124865404 | 0.00673756952817199 |

|  | **Granger test: PRx on HRV_LF** | | **Granger test: HRV_LF on PRx** | | **Granger test: PRx on HRV_VLF** | | **Granger test: HRV_VLF on PRx** | |
| --- | --- | --- | --- | --- | --- | --- | --- | --- |
|  | F-test value | P-value | F-test value | P-value | F-test value | P-value | F-test value | P-value |
| Patient 1 | 1.3602975203291 | 0.245045824232195 | 0.869824361586363 | 0.481079287012553 | 0.662376404894603 | 0.618097916966879 | 0.416337326072741 | 0.796995601233386 |
| Patient 2 | 1.8361170225297 | 0.119416814832558 | 4.55323318114382 | 0.00117505924699237 | 1.5941093803738 | 0.17346496402709 | 1.31475560216701 | 0.262375679950124 |
| Patient 3 | 1.87695787671204 | 0.111868318536498 | 2.53540780829124 | 0.0384730388943165 | 7.00836959960215 | 1.35435555288019e-05 | 1.42466915228606 | 0.223314698476363 |
| Patient 4 | 2.04231096866949 | 0.08713572561371 | 0.654793589516263 | 0.62368905397142 | 2.19708322227646 | 0.0682250922218096 | 1.2705931730636 | 0.280504548053288 |
| Patient 5 | 2.40048454287457 | 0.0477918255085347 | 7.29733117510844 | 7.33645697728951e-06 | 2.50538037081618 | 0.0401697801203345 | 1.69618632810291 | 0.14785228674052 |
| Patient 6 | 0.40251692957325 | 0.806948094377419 | 1.04764578390739 | 0.381177502876286 | 0.0513604397473551 | 0.995066455543805 | 0.672322858256411 | 0.611194287051828 |
| Patient 7 | 9.33031989345388 | 1.60813366806856e-07 | 7.45674005940254 | 5.4382511058891e-06 | 0.4720782324657 | 0.756288798638136 | 0.196367816531853 | 0.940376039342738 |
| Patient 8 | 2.1248364663841 | 0.0753329091002685 | 1.96382208417968 | 0.0974774225136708 | 1.12485994424875 | 0.342980089532014 | 1.88485102295564 | 0.110482845405318 |
| Patient 9 | 0.480794101176488 | 0.749865010610062 | 3.05671319968886 | 0.0164191601872082 | 0.880247285401798 | 0.475406683103845 | 1.74079128474493 | 0.13947484658324 |
| Patient 10 | 0.983821529872251 | 0.41513046573151 | 1.81656601441619 | 0.122987737395946 | 1.78202347123872 | 0.12983492973019 | 0.0896271618314317 | 0.985717307914454 |
| Patient 11 | 0.781600501369097 | 0.536953616495918 | 1.51680834025069 | 0.194351945900895 | 1.03158876125035 | 0.389299311823994 | 0.179201388849902 | 0.949227998478311 |
| Patient 12 | 2.47171468804052 | 0.0429494302771884 | 3.54711017556859 | 0.00693063295073303 | 0.760373898883008 | 0.551148100432236 | 1.26413167615808 | 0.282264884419535 |
| Patient 13 | 1.32516855978101 | 0.258642391363456 | 1.18461430768745 | 0.315928642822448 | 1.1647798076963 | 0.324881928786464 | 2.69734483638365 | 0.0296901467212322 |
| Patient 14 | 0.478977222247209 | 0.751218401512771 | 0.0611917825954669 | 0.993092218903412 | 0.0634766859646112 | 0.992588645125063 | 0.155046312876625 | 0.96078103206807 |
| Patient 15 | 1.57742068500939 | 0.177481925406916 | 0.792096545058567 | 0.53015020465133 | 0.280532318983725 | 0.89071650663158 | 1.1111999131884 | 0.349383132239548 |
| Patient 16 | 0.264106503145431 | 0.900513721139695 | 0.217440820525057 | 0.928314119790849 | 0.980388679769601 | 0.421542068988773 | 0.521845904954648 | 0.71985755955014 |
| Patient 17 | 1.82873284140935 | 0.120833560536339 | 0.891289317043072 | 0.468351009718045 | 2.38372153312398 | 0.0496284944887357 | 0.844407162167091 | 0.497022485926027 |
| Patient 18 | 1.31235573361695 | 0.262730418366114 | 1.63015618642583 | 0.163588063874944 | 1.56958391783511 | 0.179404053219569 | 0.338731833726537 | 0.851981544497075 |
| Patient 19 | 2.76607331169021 | 0.025940079665717 | 0.569735292114954 | 0.684615148614136 | 3.44199432083981 | 0.00812269691699698 | 0.782027265144729 | 0.536669748670399 |
| Patient 20 | 1.88089424423327 | 0.110815020565944 | 1.5980678897932 | 0.17184834559948 | 0.326831309408456 | 0.860118522552276 | 0.687853830932492 | 0.600278281690362 |
| Patient 21 | 3.67858070848217 | 0.00541179467233872 | 2.58701542738959 | 0.0351849637419162 | 0.181253208021411 | 0.948185126113798 | 5.18130238016295 | 0.000372074739305674 |
| Patient 22 | 5.0879827739704 | 0.000432768345251448 | 5.24032267158279 | 0.000328175561273127 | 0.0825529831836419 | 0.987776988026875 | 1.5002994582854 | 0.199260741520477 |
| Patient 23 | 0.474291397820678 | 0.754662277319795 | 0.916234048960244 | 0.453330883431665 | 0.861382029492145 | 0.486289276977211 | 0.0830023613607279 | 0.987651169268343 |
| Patient 24 | 1.1202895440156 | 0.345018517627707 | 1.99222530228789 | 0.0930314893031651 | 2.23749020801589 | 0.0626524892126116 | 1.53940196534289 | 0.188014672788258 |
| Patient 25 | 1.02250172547835 | 0.394193587794238 | 0.429124116226747 | 0.787700749049055 | 0.127629949095829 | 0.972452184598785 | 0.829129100510119 | 0.506472218624582 |

|  | **Granger test: PRx on HRV_LF** | | **Granger test: HRV_LF on PRx** | | **Granger test: PRx on HRV_VLF** | | **Granger test: HRV_VLF on PRx** | |
| --- | --- | --- | --- | --- | --- | --- | --- | --- |
|  | F-test value | P-value | F-test value | P-value | F-test value | P-value | F-test value | P-value |
| Patient 26 | 0.54272990679217 | 0.704377814303734 | 0.750876682764606 | 0.557395244052093 | 0.761848655566513 | 0.550101814753686 | 1.29882362826009 | 0.2683731420767 |
| Patient 27 | 1.66776621042354 | 0.154588269083528 | 7.3880616893363 | 6.37039867904888e-06 | 0.171125007434853 | 0.953209347343045 | 1.06726716684539 | 0.371006418228005 |
| Patient 28 | 0.413158217552548 | 0.799265158446954 | 2.43043262529704 | 0.0458997208818186 | 1.19081460837401 | 0.313027026473764 | 1.53730012595174 | 0.188986420225406 |
| Patient 29 | 1.91086379205475 | 0.10595118088925 | 1.53945898209415 | 0.188072693509643 | 0.44340514120806 | 0.77728785183246 | 0.395672502018011 | 0.811872264251687 |
| Patient 30 | 0.596342152038199 | 0.665474351172935 | 0.493175544125911 | 0.740768637208175 | 0.69546325477614 | 0.595463109849835 | 0.630969206303419 | 0.640675770327777 |
| Patient 31 | 2.11733521936874 | 0.0759875564668191 | 13.933714476322 | 2.62502692874321e-11 | 0.213365122213593 | 0.93112526845188 | 0.428665734515731 | 0.788042364536531 |
| Patient 32 | 1.06487536902401 | 0.372536954105746 | 0.862435368954715 | 0.485885124283982 | 1.13535124212388 | 0.33829879391501 | 0.501972730227519 | 0.734308903990734 |
| Patient 33 | 2.45807236320299 | 0.043843126320288 | 1.24627931274451 | 0.289372558184839 | 3.30309290822711 | 0.0105397703978749 | 1.01872019208384 | 0.396398866517729 |
| Patient 34 | 0.79017654567123 | 0.531560698678595 | 0.53123842858067 | 0.712810744824359 | 1.72749525526803 | 0.141656915489965 | 0.577255702246145 | 0.67919246335484 |
| Patient 35 | 2.66837429194924 | 0.03064352723702 | 0.456937818088048 | 0.767393890885912 | 0.438433689939601 | 0.780922846943858 | 0.272595318529712 | 0.895770986908284 |
| Patient 36 | 0.447077444621494 | 0.774571737039834 | 0.841069839154965 | 0.499319833958521 | 1.24012555406665 | 0.292511891113748 | 1.65638653269226 | 0.158429841936805 |
| Patient 37 | 3.78497591852934 | 0.0044806010896381 | 1.81449397013427 | 0.123154619836624 | 1.34609232226746 | 0.250364667389293 | 0.340442127705728 | 0.850786379367101 |
| Patient 38 | 2.92761648225907 | 0.0196829237277555 | 1.74258468007448 | 0.13754853042303 | 0.26797045618251 | 0.898699449167663 | 0.862786734029004 | 0.485384654818312 |
| Patient 39 | 8.33863864373403 | 1.04624413497491e-06 | 2.9655536894766 | 0.0184708676001397 | 2.76144651421476 | 0.0261420316284205 | 2.79074778909921 | 0.0248776833555551 |
| Patient 40 | 0.61533765110204 | 0.651615537209167 | 1.56103265483784 | 0.182052495460096 | 0.335336472891359 | 0.854285817392217 | 0.367864351620968 | 0.831651864936989 |
| Patient 41 | 1.21631064092009 | 0.302198630354709 | 0.420586696316953 | 0.793870268682293 | 1.05108384168076 | 0.379767106714091 | 0.44392912582429 | 0.776878801228385 |
| Patient 42 | 4.42369372831524 | 0.00143821796275205 | 2.85009100605697 | 0.0225446739751659 | 2.84592366167735 | 0.022734383010993 | 3.96132615641106 | 0.00328334327087757 |
| Patient 43 | 8.99536577994784 | 3.15027245168793e-07 | 1.29301463894616 | 0.270305794379199 | 0.426116048781828 | 0.789892496585993 | 0.0919751021132043 | 0.985012558725054 |
| Patient 44 | 0.600022528021986 | 0.662726657179032 | 0.781151396274084 | 0.53757666063284 | 1.06784872523786 | 0.371415533020967 | 1.27645496138615 | 0.277762770942595 |
| Patient 45 | 1.82432263766272 | 0.1236519379087 | 3.74386821690373 | 0.00536496040271254 | 0.849816410958886 | 0.494497042770879 | 0.837794147875077 | 0.501958455502588 |
| Patient 46 | 5.25526219511547 | 0.000320683068155928 | 15.7176616176058 | 9.0393348554191e-13 | 7.91437106075054 | 2.38348165301659e-06 | 25.3544270592227 | 9.39667357006028e-21 |
| Patient 47 | 3.98298232407683 | 0.00320522245967133 | 5.99593709419942 | 8.65447335273929e-05 | 6.02973754570885 | 8.17825164523979e-05 | 0.91732342326332 | 0.452896401830118 |

|  | **Granger test: PRx on HRV_LF_HF** | | **Granger test: HRV_LF_HF on PRx** | | **Granger test: PRx on HRV_TOT** | | **Granger test: HRV_TOT on PRx** | |
| --- | --- | --- | --- | --- | --- | --- | --- | --- |
|  | F-test value | P-value | F-test value | P-value | F-test value | P-value | F-test value | P-value |
| Patient 1 | 0.273391546227081 | 0.895281816354697 | 0.323632016995342 | 0.862293536146611 | 0.416775076353282 | 0.796678382443265 | 0.63717241939521 | 0.635954940647483 |
| Patient 2 | 0.314019844131037 | 0.868720252488115 | 0.464526394752124 | 0.761817202206335 | 2.18963676041757 | 0.0680653212047795 | 2.04804388471425 | 0.0854664577489145 |
| Patient 3 | 1.37283246207857 | 0.240983574772888 | 5.26574904668132 | 0.000324400016753829 | 1.87367389743996 | 0.112474719167393 | 2.83101265612578 | 0.0234578300735475 |
| Patient 4 | 1.30603281625596 | 0.266670891873297 | 2.33037413415277 | 0.0550527791281807 | 0.811434636338149 | 0.518219299230176 | 3.02340064604971 | 0.017547707233124 |
| Patient 5 | 3.77304514014524 | 0.00454416751953954 | 1.96390197245527 | 0.0971498902567829 | 2.91191419988877 | 0.0202477456771826 | 0.580016860686548 | 0.677128308314599 |
| Patient 6 | 0.508068018892885 | 0.729826960334859 | 0.734103083428881 | 0.568672279037215 | 0.301862953709505 | 0.876819281398113 | 0.425195661285562 | 0.790545676702105 |
| Patient 7 | 1.3889064294988 | 0.235006479146317 | 0.703002533604409 | 0.589789812349512 | 10.0698456111335 | 3.99540761278361e-08 | 2.67423184990838 | 0.0302755552836359 |
| Patient 8 | 0.524620344353628 | 0.717661900839684 | 0.810134346537114 | 0.518586191621188 | 4.12257493173524 | 0.00249887563938081 | 1.40772971488203 | 0.228932727407274 |
| Patient 9 | 0.437771450309006 | 0.781348398031738 | 0.988915317178071 | 0.41297319391467 | 0.0557224605334794 | 0.994215210258067 | 1.50781108465761 | 0.198411646148476 |
| Patient 10 | 0.505257495125033 | 0.73189314532486 | 2.58827663595704 | 0.0352696336648538 | 0.788310671370122 | 0.532698898291835 | 0.889790284748332 | 0.469197104697606 |
| Patient 11 | 0.172251457631608 | 0.952665867744529 | 2.34909326534296 | 0.0520813552628512 | 1.10746035227112 | 0.351089128428408 | 0.373373969431565 | 0.827778418648347 |
| Patient 12 | 0.769276876455503 | 0.545259143600334 | 0.315045362834319 | 0.868021766992708 | 2.79449783651948 | 0.0251333372206635 | 3.17082875853588 | 0.0132843069081803 |
| Patient 13 | 0.679296464377224 | 0.606402253686092 | 1.52403543222957 | 0.193098135972269 | 0.162631102364657 | 0.95722927536007 | 1.87892106528899 | 0.112039277817747 |
| Patient 14 | 3.48871757764441 | 0.00751608556531998 | 0.0860107979937964 | 0.986790732123423 | 0.534110605990731 | 0.710688597703144 | 0.838632039527147 | 0.500446758858727 |
| Patient 15 | 4.12816636381697 | 0.00245062808577192 | 0.520941075224535 | 0.720361637451476 | 1.38015616730767 | 0.238234133991331 | 1.54838501803266 | 0.185487045805798 |
| Patient 16 | 2.52432238343872 | 0.0452015431388646 | 1.90907330551452 | 0.114346320690057 | 0.777339110743676 | 0.54235462497961 | 0.336946496935963 | 0.85251218184314 |
| Patient 17 | 1.59106918969564 | 0.174292829469084 | 0.459762257887846 | 0.765308838746631 | 2.99725400684584 | 0.0177986912530285 | 0.311751798846569 | 0.870236400998001 |
| Patient 18 | 0.908622531503311 | 0.457773745388039 | 0.456091677648769 | 0.768018198912223 | 0.585978013777677 | 0.672794144392049 | 0.448512047556653 | 0.773567366984348 |
| Patient 19 | 1.91281889512805 | 0.10537582692256 | 2.67366951299861 | 0.0303202739601568 | 4.2725993710778 | 0.00187482305778851 | 0.939751126926651 | 0.439683962098519 |
| Patient 20 | 30.6330102938519 | 2.81423347834989e-25 | 3.86410922905642 | 0.00387231715152398 | 0.758696888760963 | 0.552064771061543 | 0.542934188398361 | 0.704212629781348 |
| Patient 21 | 2.42450472429236 | 0.0461235501802621 | 1.01755165288703 | 0.396816526046554 | 1.44751477776778 | 0.215743439564647 | 4.56748109523255 | 0.00112300052258995 |
| Patient 22 | 0.950465009663872 | 0.433594700274754 | 1.02811183428624 | 0.391130720634235 | 2.21396049090663 | 0.0649867551511511 | 1.2799818634128 | 0.275390491019371 |
| Patient 23 | 0.544557253392857 | 0.703024457837567 | 0.530017842286175 | 0.713690769130035 | 1.15482579727319 | 0.328779913777277 | 0.183720099549845 | 0.946946428333162 |
| Patient 24 | 5.30728453277843 | 0.000295053932522323 | 0.246026993882026 | 0.912168581790036 | 2.91839701658728 | 0.0201272734125248 | 1.13591989088395 | 0.337647171461481 |
| Patient 25 | 3.4472867030032 | 0.00812585204642161 | 0.261508264992644 | 0.902703056593931 | 2.07685736860198 | 0.0813273507237365 | 2.45213733889226 | 0.0440916470029179 |

|  | **Granger test: PRx on HRV_LF_HF** | | **Granger test: HRV_LF_HF on PRx** | | **Granger test: PRx on HRV_TOT** | | **Granger test: HRV_TOT on PRx** | |
| --- | --- | --- | --- | --- | --- | --- | --- | --- |
|  | F-test value | P-value | F-test value | P-value | F-test value | P-value | F-test value | P-value |
| Patient 26 | 1.79406075053847 | 0.127476201132133 | 0.800357793682838 | 0.524906628303589 | 0.291744075327029 | 0.883452100020227 | 0.661417972326071 | 0.618855381155621 |
| Patient 27 | 0.36416149143754 | 0.834270335028428 | 1.0545551386825 | 0.37746706437137 | 0.672972563515162 | 0.610690846057569 | 2.70249610445866 | 0.0289847497970446 |
| Patient 28 | 1.46164438287685 | 0.211594532866835 | 1.12034699507848 | 0.345278986331589 | 1.78621853563141 | 0.129148186969328 | 1.59585790094528 | 0.173001927477803 |
| Patient 29 | 2.46033847547876 | 0.0435105847220735 | 5.53360342292121 | 0.000197600931740055 | 0.335512177014315 | 0.854166525821339 | 1.40204249446268 | 0.230786541693722 |
| Patient 30 | 2.75460422277461 | 0.0278303476578668 | 0.13773471099805 | 0.96824641871393 | 0.611714300347943 | 0.654435177671445 | 0.202562026319558 | 0.936898050212383 |
| Patient 31 | 0.482205207458369 | 0.748845901363477 | 0.701138853510858 | 0.591090585035366 | 6.76755214847266 | 1.99355324246351e-05 | 2.82127264458374 | 0.0236584774775281 |
| Patient 32 | 2.50186461608995 | 0.0409354110138444 | 0.560422882216118 | 0.691450672476485 | 0.711771263617212 | 0.583928374863447 | 1.78417378751565 | 0.129711428994714 |
| Patient 33 | 1.4766623465713 | 0.206918544157098 | 1.01982977721856 | 0.395814043088869 | 4.37052210151122 | 0.00163163255877299 | 1.94856817646971 | 0.100101917671901 |
| Patient 34 | 1.47694999850087 | 0.207038257949655 | 0.347702510345821 | 0.845716478038742 | 1.75162453305589 | 0.136478735127089 | 0.849828213235061 | 0.493725051768895 |
| Patient 35 | 0.258388359376406 | 0.90464221284137 | 1.40377133218069 | 0.230065276306462 | 0.350068539311862 | 0.844124394012561 | 2.03308315663229 | 0.087122859702401 |
| Patient 36 | 1.50250384307086 | 0.199740246163135 | 0.964196043479185 | 0.426484021075239 | 0.713824732856803 | 0.582637778165099 | 1.10578903470036 | 0.352725246422778 |
| Patient 37 | 0.126849570379127 | 0.972764053000656 | 4.3955043422503 | 0.0015206106843958 | 0.561899604966789 | 0.690343498338021 | 1.96013698028123 | 0.0979190760144385 |
| Patient 38 | 0.368412936632206 | 0.831291241223162 | 0.975219475305435 | 0.419639487275516 | 1.42389552152056 | 0.223155663194332 | 1.60730903950854 | 0.169399928176597 |
| Patient 39 | 1.66478583325632 | 0.155182822466831 | 1.84107839151525 | 0.117989592913905 | 5.64300180061891 | 0.000156805544714917 | 1.38532970121485 | 0.236257621896919 |
| Patient 40 | 2.05034503209376 | 0.0848934098385656 | 0.699628185273464 | 0.592183200005953 | 2.66385304512185 | 0.0310133408486795 | 2.18433806174326 | 0.0684074492202075 |
| Patient 41 | 0.251030462282702 | 0.909079180242347 | 0.618354471234618 | 0.649528253777808 | 0.908795485049253 | 0.458147613428836 | 0.272880963939369 | 0.895520988313332 |
| Patient 42 | 4.64304882444368 | 0.000974437575544181 | 4.93460542298723 | 0.000576516349487917 | 2.39870250166264 | 0.0480609757244359 | 2.44469390407157 | 0.0445428551932283 |
| Patient 43 | 0.605688413308318 | 0.658547707783945 | 0.0420341841739428 | 0.996656366638708 | 4.7313804768967 | 0.000831125756527047 | 0.412312097388703 | 0.79989864484198 |
| Patient 44 | 0.310424946161326 | 0.871071243410189 | 0.854432685718571 | 0.491047120352915 | 2.23483679363631 | 0.0638007647848019 | 0.0668771152986189 | 0.991793986794425 |
| Patient 45 | 0.921002546444885 | 0.451853977188087 | 0.0398726035724926 | 0.996967672420888 | 1.28724716173806 | 0.274794548176161 | 2.52580508244773 | 0.040781173134594 |
| Patient 46 | 1.46170123921825 | 0.21112149231299 | 1.86319166318842 | 0.114054462159784 | 12.2433423060452 | 6.84772396373766e-10 | 15.1944886397094 | 2.50282411005028e-12 |
| Patient 47 | 1.30660036299243 | 0.265366584710995 | 2.6187426204864 | 0.0335427461072939 | 3.35015711928333 | 0.00967953669361823 | 2.74497409831711 | 0.0271519053558922 |

|  | **Granger test: PRx on HRV_RMS** | | **Granger test: HRV_RMS on PRx** | | **Granger test: PRx on HRV_HF_LF** | | **Granger test: HRV_HF_LF on PRx** | |
| --- | --- | --- | --- | --- | --- | --- | --- | --- |
|  | F-test value | P-value | F-test value | P-value | F-test value | P-value | F-test value | P-value |
| Patient 1 | 1.74148286254928 | 0.137808537448717 | 1.15520044502943 | 0.32854455902646 | 2.67816160175416 | 0.0300593732757608 | 7.23035693330902 | 8.28373416003648e-06 |
| Patient 2 | 1.21974982061356 | 0.300522413999654 | 1.78326279668767 | 0.129741695915312 | 2.22135349972945 | 0.0645943801179243 | 2.48099279794845 | 0.0422119604810507 |
| Patient 3 | 0.731446483163559 | 0.570461400277665 | 3.2344354672206 | 0.0118026246154504 | 0.375121199869965 | 0.826520873992104 | 1.75951703448436 | 0.134373623014692 |
| Patient 4 | 0.266313654998657 | 0.899604653954617 | 3.63016014665885 | 0.00623287118042341 | 4.61100057374173 | 0.00114019387217483 | 3.49594820743881 | 0.00784136788696636 |
| Patient 5 | 0.730401984541081 | 0.571058858258616 | 3.00602492178767 | 0.0172316872526052 | 0.0908294339011176 | 0.985365500931081 | 0.656007007740407 | 0.622591876116458 |
| Patient 6 | 0.0885785391764513 | 0.98603094651469 | 0.812415056354947 | 0.517135998839245 | 1.08995002999395 | 0.359864563425618 | 0.1587280682053 | 0.959077156929777 |
| Patient 7 | 10.8262510750059 | 9.43684161239215e-09 | 0.981955546108225 | 0.415927798731294 | 0.717308299723523 | 0.579968469280821 | 1.42227720123787 | 0.223708091517448 |
| Patient 8 | 5.13468462273833 | 0.000408286207323462 | 1.80267044317905 | 0.12563187874914 | 1.81770305073482 | 0.122716543233408 | 0.512458821480137 | 0.726597926893127 |
| Patient 9 | 0.593731640407467 | 0.667289479079505 | 1.55634261194403 | 0.184360629959138 | 0.129180837272306 | 0.97179280940551 | 0.620631496947351 | 0.647935780159052 |
| Patient 10 | 3.93252904940281 | 0.00349621741411575 | 2.26835778176101 | 0.0597299575702781 | 3.58475501055738 | 0.00642646647311044 | 2.69467588464365 | 0.0294956328599495 |
| Patient 11 | 9.79301871463368 | 6.89061368917629e-08 | 6.72567025708167 | 2.14757924456694e-05 | 4.77798503211717 | 0.000757355020051757 | 1.4596097597133 | 0.211705545937853 |
| Patient 12 | 2.75847158338647 | 0.0266554362208757 | 1.97094862135455 | 0.0966809047989121 | 1.66587085466357 | 0.155540532720408 | 0.852872128309314 | 0.491771739622517 |
| Patient 13 | 1.31777262105263 | 0.261422887085823 | 2.35493960504794 | 0.0521775297621764 | 0.868483147101845 | 0.482248106648491 | 0.907967118529493 | 0.458554428730782 |
| Patient 14 | 1.33560085951528 | 0.254190728422469 | 3.94753864675079 | 0.00336423258998384 | 3.90803175791028 | 0.0035922199119103 | 0.421519132568761 | 0.793234564962993 |
| Patient 15 | 1.58518702439136 | 0.175396831432344 | 1.06072694518072 | 0.374321446599513 | 1.84831993684407 | 0.116798402177515 | 0.669609377270518 | 0.613043607489372 |
| Patient 16 | 0.116304239019013 | 0.976517819564618 | 0.544330786619868 | 0.703490804733368 | 0.289203553994216 | 0.884541551063361 | 0.24630037496276 | 0.911445433838522 |
| Patient 17 | 3.8106430535395 | 0.00436517503028437 | 2.25814445681959 | 0.0608740408326246 | 0.205601083025481 | 0.935370585383683 | 1.15244648887183 | 0.330239335645318 |
| Patient 18 | 1.12325919707666 | 0.343441691837181 | 6.62964727143099 | 2.52541777830826e-05 | 0.914252241483264 | 0.454454214686128 | 1.43242726334346 | 0.220351069381399 |
| Patient 19 | 4.23897165203761 | 0.0019896676865912 | 4.50809978714408 | 0.00122912143377772 | 2.85134977566495 | 0.0224491636372733 | 2.01394935638435 | 0.0896970801703556 |
| Patient 20 | 4.28934234792104 | 0.00181786427074705 | 0.960391478960612 | 0.427949460428321 | 7.21385393603972 | 8.60153328923363e-06 | 6.91455937815069 | 1.50072423131069e-05 |
| Patient 21 | 9.44893191567077 | 1.38511755938702e-07 | 3.24846162938358 | 0.0114325560907584 | 0.764592334111797 | 0.548201671415804 | 3.5423032038717 | 0.00686633906570216 |
| Patient 22 | 4.74591130681258 | 0.000803358345023623 | 0.345377699361006 | 0.847387323396002 | 4.24641487493736 | 0.00196747681503098 | 0.91464646464484 | 0.45426650867687 |
| Patient 23 | 1.47827594872866 | 0.205900635911017 | 0.861119198790861 | 0.486443243567494 | 4.25392583204645 | 0.00194022558639688 | 1.19502188607476 | 0.310752544112918 |
| Patient 24 | 2.30576851556602 | 0.0560255370053035 | 2.58931051912768 | 0.0350417990851156 | 1.14241517966472 | 0.334605295269264 | 4.18862866096907 | 0.00219893048671878 |
| Patient 25 | 0.472376249327305 | 0.756062493964501 | 3.6117344254215 | 0.00612399627191378 | 0.264733767666247 | 0.90070010015407 | 2.46381873912612 | 0.0432184672295168 |

|  | **Granger test: PRx on HRV_RMS** | | **Granger test: HRV_RMS on PRx** | | **Granger test: PRx on HRV_HF_LF** | | **Granger test: HRV_HF_LF on PRx** | |
| --- | --- | --- | --- | --- | --- | --- | --- | --- |
|  | F-test value | P-value | F-test value | P-value | F-test value | P-value | F-test value | P-value |
| Patient 26 | 0.383095264860775 | 0.820850945195956 | 3.67967564842063 | 0.00545871537746631 | 3.06523549427785 | 0.015773913178049 | 1.22492598759838 | 0.298209725117334 |
| Patient 27 | 1.43003015258343 | 0.221293177549035 | 0.981166202622049 | 0.416444176453847 | 1.60955602291776 | 0.168982807393155 | 2.46371536721855 | 0.0431389135784256 |
| Patient 28 | 0.142198494991513 | 0.966435824616824 | 0.796664072719426 | 0.527311118373306 | 2.71303597639648 | 0.0286932080455975 | 1.12878926568924 | 0.341239114593151 |
| Patient 29 | 2.70319047626577 | 0.0290103687187645 | 0.435963077001692 | 0.782719011455596 | 1.243624339541 | 0.290266172554938 | 0.845322655502997 | 0.49631906115571 |
| Patient 30 | 0.924443185221086 | 0.449550958201754 | 0.397746955848614 | 0.81027052203533 | 2.0803423856415 | 0.0825290965474849 | 0.918314353728346 | 0.453098518874862 |
| Patient 31 | 5.38369147369373 | 0.000252754264395924 | 2.30522650359174 | 0.055959770743365 | 0.77798023091421 | 0.539329211481741 | 2.4740962279629 | 0.0423443232136013 |
| Patient 32 | 1.00801607988143 | 0.402152275380396 | 2.38126420609406 | 0.049857402106581 | 2.26698175082576 | 0.0600772774414646 | 1.34091734635863 | 0.252667568756796 |
| Patient 33 | 0.848826011830099 | 0.494235308157262 | 1.24509255461145 | 0.289858856349658 | 0.739584006878091 | 0.564990208062289 | 0.931130370975401 | 0.444896806292098 |
| Patient 34 | 0.941773155344513 | 0.438858224157813 | 0.494606121632614 | 0.73972407006597 | 1.58826533396093 | 0.175085089599344 | 1.00486558533096 | 0.403854028481286 |
| Patient 35 | 0.856580006388294 | 0.489267430772405 | 2.01434347070821 | 0.0897258177323115 | 4.44050433107594 | 0.00139598796033838 | 0.113375717874023 | 0.977858539542478 |
| Patient 36 | 2.04997235044886 | 0.0857498783487629 | 1.27604336239106 | 0.277890152515487 | 0.970502090417913 | 0.422928633295884 | 2.22993217412525 | 0.0642646340495283 |
| Patient 37 | 2.85031606975832 | 0.0225662584522311 | 1.55871134449166 | 0.182568419988613 | 1.7691994526257 | 0.132170663740816 | 0.165211319568439 | 0.956052003638025 |
| Patient 38 | 7.11846961471234 | 1.01585037251651e-05 | 0.970555897158866 | 0.422226118714274 | 3.59866852653948 | 0.0061580796929427 | 2.34635496834092 | 0.0522229690358576 |
| Patient 39 | 7.63914847710607 | 3.88454796696506e-06 | 2.42517763297663 | 0.045886581524196 | 0.088552674753857 | 0.986048152529527 | 0.125358131798525 | 0.973356239231161 |
| Patient 40 | 3.32831467754572 | 0.00998745185936617 | 0.69736865676855 | 0.593736339015369 | 1.4742158418731 | 0.207381395391983 | 1.14595675506412 | 0.333030030466473 |
| Patient 41 | 1.57025584079691 | 0.180128298357251 | 1.38489426435585 | 0.237210575654583 | 1.3700541169122 | 0.242370253903084 | 3.09237799848241 | 0.0152424978652812 |
| Patient 42 | 4.80931787714433 | 0.000719745273818383 | 3.16925276339819 | 0.0130651591480852 | 4.46919427639111 | 0.0013255941921545 | 1.57220776074214 | 0.17882254301416 |
| Patient 43 | 7.02588683139635 | 1.24644600134076e-05 | 1.46810025684443 | 0.209129958495511 | 0.675218812609376 | 0.609105216978263 | 0.837810256728453 | 0.500948522699595 |
| Patient 44 | 0.403032046513956 | 0.806534216594618 | 0.109704581499428 | 0.979136005360962 | 0.53891245423201 | 0.707195831010484 | 0.487134327808941 | 0.745212327614835 |
| Patient 45 | 0.235324634107157 | 0.918349196023316 | 0.146684518956023 | 0.96439361476489 | 3.58642349901827 | 0.00699923311685693 | 0.353393602249648 | 0.841623041363148 |
| Patient 46 | 8.17268821528668 | 1.4633900116501e-06 | 3.58765275223008 | 0.00631560188353602 | 0.361915496306484 | 0.835853373243863 | 0.42597502490152 | 0.789998008246984 |
| Patient 47 | 4.04577473496608 | 0.00286957341642767 | 2.39330991594916 | 0.0487007812537196 | 0.860276693504997 | 0.487118266231592 | 4.45920802753005 | 0.00137925466445995 |

*Table displays Granger causality testing for Autonomic variables on PRx and PRx on Autonomic variables, using the full range of data. The larger F-test value indicates which direction is favored in the relationship between two variables (highlighted in gray). In general, the directional nature of causality favors PRx on an Autonomic response.*

*BPV_D, standard deviation of diastolic blood pressure variability; BPV_M, standard deviation of mean blood pressure variability; BPV_S, standard deviation of systolic blood pressure variability; HRF_HF, heart rate variability high frequency; HRV_HF_LF, heart rate variability ratio between high/low frequency; HRV_LF, heart rate variability low frequency; HRV_LF_HF, heart rate variability ratio between low/high frequency; HRV_RMS, heart rate variability root mean square; HRV_TOT, heart rate variability total; HRV_VLF, heart rate variability very low frequency; PRx, pressure reactivity; SBPV_HF, spectral blood pressure variability high frequency; SBPV_LF, spectral blood pressure variability low frequency; SBPV_TOT, spectral blood pressure variability total;*
