# Supplementary material for: Dynamic Temporal Relationship Between Autonomic Function and Cerebrovascular Reactivity in Moderate/Severe Traumatic Brain Injury
Source: Front Netw Physiol. 2022 Feb 16;2:837860. doi: 10.3389/fnetp.2022.837860 (PMC10013014; doi:10.3389/fnetp.2022.837860)
Supplement: Supplementary file 4 [file DataSheet3.DOCX]

Appendix C. Median values and Interquartile Range for All Patients Variables (n=47)

| Variable | Median Value (Interquartile Range) |
| --- | --- |
| PRx (au) | 0.17 (-0.15 - 0.49) |
|  |  |
| BPV_S (mmHg) | 4.79 (2.86- 8.85) |
| BPV_D (mmHg) | 2.90 (1.76 -5.04) |
| BPV_M (mmHg) | 2.96 (1.85 - 5.49) |
| SBPV_HF (mmHg^2^) | 1.92 (0.85 - 3.77) |
| SBPV_LF (mmHg^2^) | 1.58 (0.50 - 4.88) |
| SBPV_TOT (mmHg^2^) | 7.75 (4.27 - 16.20) |
|  |  |
| HRV_HF (ms^2^) | 26.67 (12.16 - 101.7) |
| HRV_LF (ms^2^) | 40.38 (10.31 - 175.20) |
| HRV_LF_HF (ratio) | 0.94 (0.44 - 1.84) |
| HRV_VLF (ms^2^) | 15 (4 - 58) |
| HRV_TOT (ms^2^) | 82.2 (32.4 - 275.4) |
| HRV_RMS (ms) | 14.17 (10.75 - 23.10) |
|  |  |
| BRS (ms/mmHg) | 6 (3 - 13) |

*Au, arbitrary units; BPV_D, standard deviation of diastolic blood pressure variability; BPV_M, standard deviation of mean blood pressure variability; BPV_S, standard deviation of systolic blood pressure variability; BRS, baroreflex sensitivity; HRF_HF, heart rate variability high frequency; HRV_LF, heart rate variability low frequency; HRV_LF_HF, heart rate variability ratio between low/high frequency; HRV_RMS, heart rate variability root mean square; HRV_TOT, heart rate variability total; HRV_VLF, heart rate variability very low frequency; mmHg, millimeters of mercury; ms, millisecond; PRx, pressure reactivity; SBPV_HF, spectral blood pressure variability high frequency; SBPV_LF, spectral blood pressure variability low frequency; SBPV_TOT, spectral blood pressure variability total;*
