# Supplementary material for: Dynamic Temporal Relationship Between Autonomic Function and Cerebrovascular Reactivity in Moderate/Severe Traumatic Brain Injury
Source: Front Netw Physiol. 2022 Feb 16;2:837860. doi: 10.3389/fnetp.2022.837860 (PMC10013014; doi:10.3389/fnetp.2022.837860)

Appendix D. Granger Causality F Statistic Comparisons

*The box plots show the Granger Causality F-Statistics for all patients, PRx on an ARV and an ARV on PRx. The p-value is a Mann-Whitney U test comparing the mean F value. ARV, autonomic response variable; BPV_D, standard deviation of diastolic blood pressure variability; BPV_M, standard deviation of mean blood pressure variability; BPV_S, standard deviation of systolic blood pressure variability; HRV, heart rate variability; HRF_HF, heart rate variability high frequency; HRV_HF_LF, heart rate variability ratio between high/low frequency; HRV_LF, heart rate variability low frequency; HRV_LF_HF, heart rate variability ratio between low/high frequency; HRV_RMS, heart rate variability root mean square; HRV_TOT, heart rate variability total; HRV_VLF, heart rate variability very low frequency; PRx, pressure reactivity; SBPV_HF, spectral blood pressure variability high frequency; SBPV_LF, spectral blood pressure variability low frequency; SBPV_TOT, spectral blood pressure variability total;*


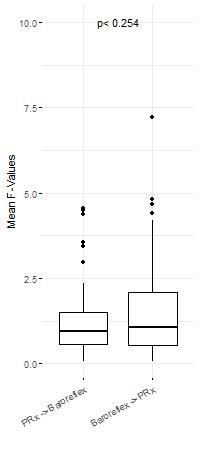

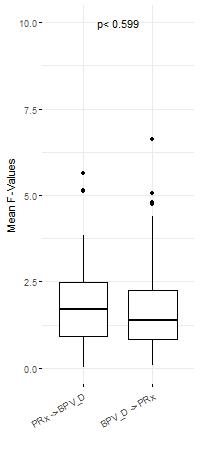

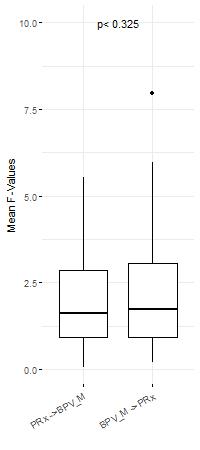


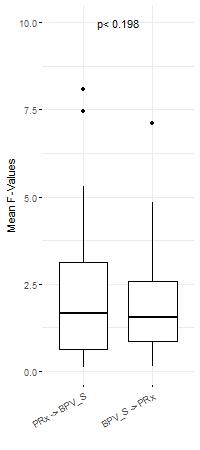

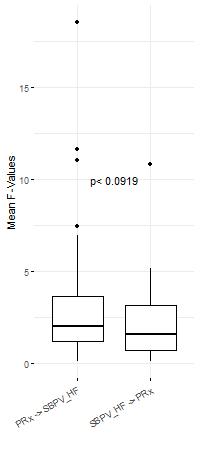

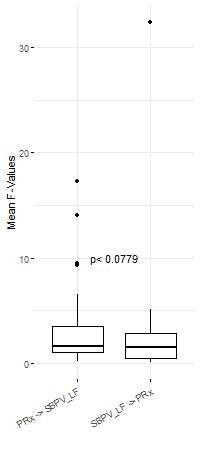

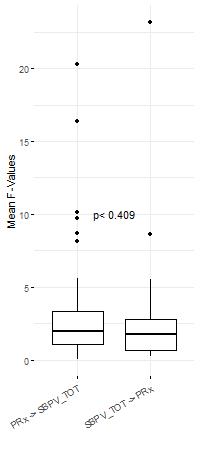

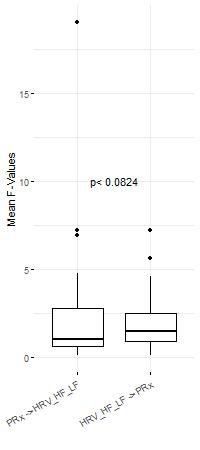

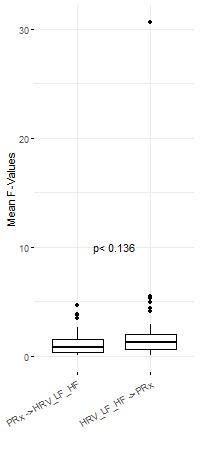

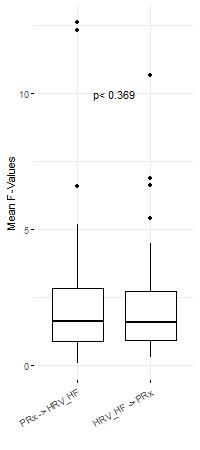

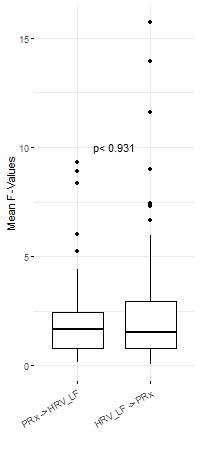

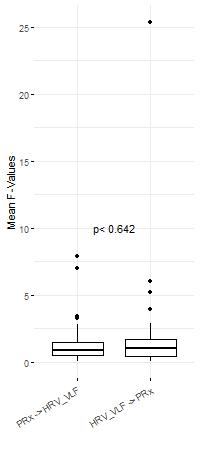

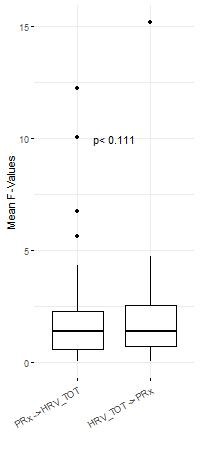

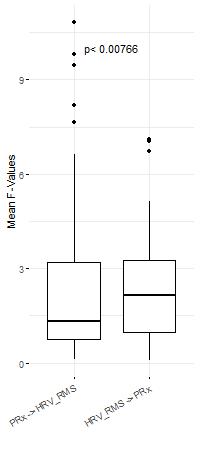

Supplement: Supplementary file 5 [file DataSheet5.DOCX]
