# Supplementary material for: Dynamic Temporal Relationship Between Autonomic Function and Cerebrovascular Reactivity in Moderate/Severe Traumatic Brain Injury
Source: Front Netw Physiol. 2022 Feb 16;2:837860. doi: 10.3389/fnetp.2022.837860 (PMC10013014; doi:10.3389/fnetp.2022.837860)
Supplement: Supplementary file 6 [file DataSheet2.DOCX]

Appendix B. PRx and Autonomic Variable variant ARIMA structure - patient example

| **ARIMA** | **PRx** | | **HRV_HF** | | **HRV_LF** | | **HRV_VLF** | | **HRV_LF_HF** | | **HRV_HF_LF** | |
| --- | --- | --- | --- | --- | --- | --- | --- | --- | --- | --- | --- | --- |
| **Model** | **LL** | **AIC** | **LL** | **AIC** | **LL** | **AIC** | **LL** | **AIC** | **LL** | **AIC** | **LL** | **AIC** |
| (1,0,0) | -3.580287418 | 13.16057484 | -63.65165977 | 133.3033195 | -68.58579073 | 143.1715815 | -89.0866226 | 184.1732452 | 31.32096891 | -56.64193781 | 43.24605634 | -80.49211269 |
| (1,0,1) | -2.253586656 | 12.50717331 | -61.92097587 | 131.8419517 | -67.82721705 | 143.6544341 | -88.43458251 | 184.869165 | 32.55689532 | -57.11379064 | 43.24605634 | -80.49211269 |
| (1,0,2) | -2.164668445 | 14.32933689 | -61.23766204 | 132.4753241 | -62.97566507 | 135.9513301 | -85.42246433 | 180.8449287 | 32.77526197 | -55.55052394 | 43.24605634 | -80.49211269 |
| (1,0,3) | -1.561748082 | 15.12349616 | -59.38244763 | 130.7648953 | -62.0812535 | 136.162507 | -81.33339017 | 174.6667803 | 33.63782285 | -55.2756457 | 43.24605634 | -80.49211269 |
| (1,0,4) | -1.556036888 | 17.11207378 | -58.59541238 | 131.1908248 | -58.03429633 | 130.0685927 | -79.50243712 | 173.0048742 | 33.64247095 | -53.2849419 | 43.24605634 | -80.49211269 |
| (1,0,5) | -1.388634476 | 18.77726895 | -58.46407493 | 132.9281499 | -58.03160076 | 132.0632015 | -79.59305695 | 175.1861139 | 34.43548259 | -52.87096518 | 43.24605634 | -80.49211269 |
| (1,0,6) | 0.673998157 | 16.65200369 | -58.31672135 | 134.6334427 | -57.81258859 | 133.6251772 | -76.61504765 | 171.2300953 | 35.53481393 | -53.06962786 | 43.24605634 | -80.49211269 |
| (1,0,7) | 1.320298056 | 17.35940389 | -58.14149027 | 136.2829805 | -57.81255419 | 135.6251084 | -76.44473994 | 172.8894799 | 35.54113827 | -51.08227654 | 43.24605634 | -80.49211269 |
| (1,0,8) | 1.376294776 | 19.24741045 | -57.57240048 | 137.144801 | -57.80846874 | 137.6169375 | -76.18358497 | 174.3671699 | 36.94869294 | -51.89738589 | 75.21569053 | -128.4313811 |
| (1,0,9) | 2.585633763 | 18.82873247 | -58.11558331 | 140.2311666 | -57.32224628 | 138.6444926 | -75.68161424 | 175.3632285 | 36.52474381 | -49.04948761 | 75.21569053 | -128.4313811 |
| (1,0,10) | 3.354828669 | 19.29034266 | -57.27912234 | 140.5582447 | -57.29626887 | 140.5925377 | -74.54279939 | 175.0855988 | 36.62226107 | -47.24452213 | 76.54129071 | -127.0825814 |
| (2,0,0) | -1.800127929 | 11.60025586 | -63.60421673 | 135.2084335 | -66.84920092 | 141.6984018 | -87.69167706 | 183.3833541 | 31.97313508 | -55.94627017 | 72.66482425 | -137.3296485 |
| (2,0,1) | -1.117231386 | 12.23446277 | -61.53382562 | 133.0676512 | -68.4657476 | 146.9314952 | -82.28614831 | 174.5722966 | 32.6626709 | -55.3253418 | 72.80511976 | -135.6102395 |
| (2,0,2) | -1.886567123 | 15.77313425 | -60.53089591 | 133.0617918 | -59.52128208 | 131.0425642 | -78.66465685 | 169.3293137 | 32.75290721 | -53.50581441 | 72.80511976 | -135.6102395 |
| (2,0,3) | 1.030539938 | 11.93892012 | -58.048203 | 130.096406 | -62.97565846 | 139.9513169 | -78.66885091 | 171.3377018 | 33.69417044 | -53.38834089 | 72.80511976 | -135.6102395 |
| (2,0,4) | 1.035361874 | 13.92927625 | -58.03588987 | 132.0717797 | -58.02795084 | 132.0559017 | -78.32874497 | 172.6574899 | 33.69897343 | -51.39794685 | 77.54496101 | -139.089922 |
| (2,0,5) | 0.213272179 | 17.57345564 | -57.95773367 | 133.9154673 | -57.98969686 | 133.9793937 | -78.30765814 | 174.6153163 | 35.47484484 | -52.94968969 | 77.68593974 | -137.3718795 |
| (2,0,6) | 1.588098838 | 16.82380232 | -57.95010969 | 135.9002194 | -57.81254598 | 135.625092 | -76.38319813 | 172.7663963 | 36.18149929 | -52.36299858 | 75.05540989 | -130.1108198 |
| (2,0,7) | 1.396501949 | 19.2069961 | -58.13725927 | 138.2745185 | -57.72572933 | 137.4514587 | -74.57885949 | 171.157719 | 37.04320745 | -52.08641491 | 75.51464449 | -129.029289 |
| (2,0,8) | 1.720493736 | 20.55901253 | -58.13725233 | 140.2745047 | -57.68348915 | 139.3669783 | -76.76494603 | 177.5298921 | 36.3127596 | -48.62551921 | 64.85062752 | -105.701255 |
| (2,0,9) | 3.308302531 | 19.38339494 | -57.30084289 | 140.6016858 | -56.83008364 | 139.6601673 | -76.0645543 | 178.1291086 | 37.3407512 | -48.6815024 | 77.00999334 | -128.0199867 |
| (2,0,10) | 3.373053793 | 21.25389241 | -57.12180946 | 142.2436189 | -56.82881954 | 141.6576391 | -74.517294 | 177.034588 | 38.06395092 | -48.12790184 | 77.06018025 | -126.1203605 |
| (3,0,0) | -1.461361773 | 12.92272355 | -60.1455452 | 130.2910904 | -59.7826718 | 129.5653436 | -82.50447597 | 175.0089519 | 33.62694224 | -57.25388448 | 72.87923016 | -135.7584603 |
| (3,0,1) | -0.258012857 | 12.51602571 | -59.91037504 | 131.8207501 | -59.75499095 | 131.5099819 | -80.99136088 | 173.9827218 | 33.92071571 | -55.84143142 | 72.68177458 | -133.3635492 |
| (3,0,2) | 0.102846773 | 13.79430645 | -59.76513135 | 133.5302627 | -59.35456817 | 132.7091363 | -78.64704582 | 171.2940916 | 33.92099418 | -53.84198836 | 78.14796774 | -142.2959355 |
| (3,0,3) | -0.579697931 | 17.15939586 | -58.04074649 | 132.081493 | -59.32966672 | 134.6593334 | -78.64704582 | 171.2940916 | 33.96764148 | -51.93528297 | 71.94737062 | -127.8947412 |
| (3,0,4) | 1.262901904 | 15.47419619 | -57.78656014 | 133.5731203 | -57.8276164 | 133.6552328 | -78.55758647 | 175.1151729 | 35.21286881 | -52.42573762 | 69.91334732 | -121.8266946 |
| (3,0,5) | 0.449872221 | 19.10025556 | -57.69423723 | 135.3884745 | -56.7424754 | 133.4849508 | -77.42060079 | 174.8412016 | 35.51500193 | -51.03000385 | 72.19206626 | -124.3841325 |
| (3,0,6) | 1.943626145 | 18.11274771 | -57.95534478 | 137.9106896 | -56.36349612 | 134.7269922 | -76.37204017 | 174.7440803 | 37.17360603 | -52.34721206 | 73.07897156 | -124.1579431 |
| (3,0,7) | 1.846901004 | 20.30619799 | -56.99937574 | 137.9987515 | -56.64816375 | 137.2963275 | -76.77431247 | 177.5486249 | 37.12501299 | -50.25002598 | 77.61798029 | -131.2359606 |
| (3,0,8) | 1.847531017 | 22.30493797 | -57.64413545 | 141.2882709 | -56.64718757 | 139.2943751 | -76.09996908 | 178.1999382 | 37.68930647 | -49.37861295 | 75.99653204 | -125.9930641 |
| (3,0,9) | 3.162331831 | 21.67533634 | -56.8505709 | 141.7011418 | -56.82661192 | 141.6532238 | -72.85766505 | 173.7153301 | 36.68764227 | -45.37528454 | 75.38532187 | -122.7706437 |
| (3,0,10) | 3.532768526 | 22.93446295 | -56.57243663 | 143.1448733 | -55.94393155 | 141.8878631 | -72.75789687 | 175.5157937 | 36.99987749 | -43.99975497 | 78.17515248 | -126.350305 |
| (4,0,0) | -0.840945154 | 13.68189031 | -60.02436462 | 132.0487292 | -59.75101629 | 131.5020326 | -80.97358022 | 173.9471604 | 33.82857665 | -55.65715329 | 73.6246087 | -135.2492174 |
| (4,0,1) | -0.730654064 | 15.46130813 | -59.65750135 | 133.3150027 | -59.69804182 | 133.3960836 | -80.79807978 | 175.5961596 | 33.92088126 | -53.84176251 | 46.55282045 | -79.1056409 |
| (4,0,2) | -0.717985759 | 17.43597152 | -59.64817367 | 135.2963473 | -59.35456299 | 134.709126 | -80.9736894 | 177.9473788 | 33.92239625 | -51.8447925 | 68.04537227 | -120.0907445 |
| (4,0,3) | 0.977688697 | 16.04462261 | -57.93893868 | 133.8778774 | -59.64554042 | 137.2910808 | -78.62694627 | 175.2538925 | 35.6035104 | -53.20702079 | 74.33350027 | -130.6670005 |
| (4,0,4) | 2.398121316 | 15.20375737 | -57.9389503 | 135.8779006 | -57.68553564 | 135.3710713 | -76.36212081 | 172.7242416 | 35.67425968 | -51.34851936 | 74.33350027 | -130.6670005 |
| (4,0,5) | 2.532426356 | 16.93514729 | -57.48521493 | 136.9704299 | -57.5637294 | 137.1274588 | -74.18836583 | 170.3767317 | 35.98422733 | -49.96845465 | 73.61695503 | -125.2339101 |
| (4,0,6) | 2.485676647 | 19.02864671 | -57.44130067 | 138.8826013 | -56.65288191 | 137.3057638 | -74.10087641 | 172.2017528 | 37.19253119 | -50.38506238 | 79.01628515 | -134.0325703 |
| (4,0,7) | 2.803337188 | 20.39332562 | -57.44118353 | 140.8823671 | -56.56371379 | 139.1274276 | -73.94781208 | 173.8956242 | 37.5474675 | -49.094935 | 76.25905987 | -126.5181197 |
| (4,0,8) | 2.914190492 | 22.17161902 | -56.71564866 | 141.4312973 | -56.26228844 | 140.5245769 | -73.76709941 | 175.5341988 | 37.85360941 | -47.70721883 | 73.00639061 | -118.0127812 |
| (4,0,9) | 3.757261517 | 22.48547697 | -57.33683768 | 144.6736754 | -56.46416178 | 142.9283236 | -73.53935926 | 177.0787185 | 37.69333663 | -45.38667326 | 78.71252636 | -127.4250527 |
| (4,0,10) | 4.043014085 | 23.91397183 | -56.51468473 | 145.0293695 | -55.6043076 | 143.2086152 | -72.73456869 | 177.4691374 | 37.09554501 | -42.19109002 | 73.88977567 | -115.7795513 |

| **ARIMA** | **PRx** | | **HRV_HF** | | **HRV_LF** | | **HRV_VLF** | | **HRV_LF_HF** | | **HRV_HF_LF** | |
| --- | --- | --- | --- | --- | --- | --- | --- | --- | --- | --- | --- | --- |
| **Model** | **LL** | **AIC** | **LL** | **AIC** | **LL** | **AIC** | **LL** | **AIC** | **LL** | **AIC** | **LL** | **AIC** |
| (5,0,0) | -0.804040169 | 15.60808034 | -59.93024667 | 133.8604933 | -59.73234082 | 133.4646816 | -80.56123804 | 175.1224761 | 33.93678732 | -53.87357464 | 77.28591283 | -140.5718257 |
| (5,0,1) | -0.724266495 | 17.44853299 | -59.6435596 | 135.2871192 | -59.69796708 | 135.3959342 | -80.21990213 | 176.4398043 | 33.94978371 | -51.89956743 | 72.1083655 | -128.216731 |
| (5,0,2) | 0.1999219 | 17.6001562 | -59.64235868 | 137.2847174 | -59.38714771 | 136.7742954 | -79.5822726 | 177.1645452 | 35.58829052 | -53.17658104 | 74.72301489 | -131.4460298 |
| (5,0,3) | 2.295002122 | 15.40999576 | -58.68161096 | 137.3632219 | -57.73497307 | 135.4699461 | -79.5822726 | 177.1645452 | 34.70490089 | -49.40980178 | 70.02196504 | -120.0439301 |
| (5,0,4) | 0.303300491 | 21.39339902 | -57.93296648 | 137.865933 | -57.68091713 | 137.3618343 | -75.81487545 | 173.6297509 | 35.83158082 | -49.66316164 | 53.96994917 | -85.93989834 |
| (5,0,5) | 0.928158456 | 22.14368309 | -57.44196352 | 138.883927 | -57.56323425 | 139.1264685 | -75.81487545 | 173.6297509 | 36.1903501 | -48.3807002 | 76.52560367 | -129.0512073 |
| (5,0,6) | 2.365141386 | 21.26971723 | -57.44085248 | 140.881705 | -56.64915715 | 139.2983143 | -73.79658121 | 173.5931624 | 37.26465186 | -48.52930372 | 77.16790912 | -128.3358182 |
| (5,0,7) | 5.102956974 | 17.79408605 | -57.4409448 | 142.8818896 | -56.48366047 | 140.9673209 | -73.46332128 | 174.9266426 | 37.39217067 | -46.78434134 | 77.76985295 | -127.5397059 |
| (5,0,8) | 5.222317536 | 19.55536493 | -56.71297481 | 143.4259496 | -56.4853137 | 142.9706274 | -73.4075037 | 176.8150074 | 37.8106656 | -45.6213312 | 74.00277056 | -118.0055411 |
| (5,0,9) | 5.265100149 | 21.4697997 | -57.31122254 | 146.6224451 | -56.18686582 | 144.3737316 | -73.13774756 | 178.2754951 | 37.42980609 | -42.85961219 | 76.76887863 | -121.5377573 |
| (5,0,10) | 6.79618114 | 20.40763772 | -56.31151963 | 146.6230393 | -55.38130761 | 144.7626152 | -73.35691808 | 180.7138362 | 38.30315531 | -42.60631062 | 81.0057429 | -128.0114858 |
| (6,0,0) | -0.751251007 | 17.50250201 | -59.09715089 | 134.1943018 | -59.59419648 | 135.188393 | -79.78101018 | 175.5620204 | 33.97589248 | -51.95178496 | 76.6236207 | -137.2472414 |
| (6,0,1) | -0.666325508 | 19.33265102 | -57.74291618 | 133.4858324 | -59.69768968 | 137.3953794 | -79.7744139 | 177.5488278 | 34.31399523 | -50.62799045 | 75.64075866 | -133.2815173 |
| (6,0,2) | 1.592431089 | 16.81513782 | -57.71022322 | 135.4204464 | -59.20604023 | 138.4120805 | -77.58806655 | 175.1761331 | 35.81138732 | -51.62277463 | 76.05130023 | -132.1026005 |
| (6,0,3) | 2.481787003 | 17.03642599 | -57.9337628 | 137.8675256 | -58.98107059 | 139.9621412 | -78.09416385 | 178.1883277 | 35.18994475 | -48.3798895 | 72.90217226 | -123.8043445 |
| (6,0,4) | 2.756027728 | 18.48794454 | -58.64010585 | 141.2802117 | -57.1505858 | 138.3011716 | -75.80665489 | 175.6133098 | 37.10773089 | -50.21546178 | 76.67964087 | -129.3592817 |
| (6,0,5) | 2.064731253 | 21.87053749 | -57.44195682 | 140.8839136 | -57.0943644 | 140.1887288 | -74.06666002 | 174.13332 | 36.29294251 | -46.58588503 | 78.09224092 | -130.1844818 |
| (6,0,6) | 4.208650602 | 19.5826988 | -57.43977485 | 142.8795497 | -56.32054157 | 140.6410831 | -74.04588314 | 176.0917663 | 36.8817049 | -45.76340979 | 79.21815429 | -130.4363086 |
| (6,0,7) | 5.207832403 | 19.58433519 | -56.9841267 | 143.9682534 | -56.02290338 | 142.0458068 | -73.31824078 | 176.6364816 | 36.87911807 | -43.75823613 | 80.05612903 | -130.1122581 |
| (6,0,8) | 5.227559252 | 21.5448815 | -57.56834862 | 147.1366972 | -56.30536891 | 144.6107378 | -71.85491632 | 175.7098326 | 36.75191429 | -41.50382858 | 78.8106643 | -125.6213286 |
| (6,0,9) | 5.62704623 | 22.74590754 | -56.53027319 | 147.0605464 | -55.3723159 | 144.7446318 | -71.77361029 | 177.5472206 | 37.9948248 | -41.9896496 | 76.48862215 | -118.9772443 |
| (6,0,10) | 4.70223686 | 26.59552628 | -56.21094775 | 148.4218955 | -55.21494423 | 146.4298885 | -71.78170366 | 179.5634073 | 38.35201993 | -40.70403986 | 80.74914242 | -125.4982848 |
| (7,0,0) | -0.547318851 | 19.0946377 | -58.4287157 | 134.8574314 | -59.41060034 | 136.8212007 | -79.7599211 | 177.5198422 | 34.15139796 | -50.30279592 | 80.74914242 | -125.4982848 |
| (7,0,1) | -0.524509078 | 21.04901816 | -58.30234375 | 136.6046875 | -59.02810892 | 138.0562178 | -79.12930001 | 178.2586 | 34.54368661 | -49.08737323 | 80.74914242 | -125.4982848 |
| (7,0,2) | 0.260941432 | 21.47811714 | -57.65234581 | 137.3046916 | -58.72648992 | 139.4529798 | -76.71165346 | 175.4233069 | 34.98326765 | -47.96653531 | 76.63940199 | -131.278804 |
| (7,0,3) | 2.518835567 | 18.96232887 | -58.23658897 | 140.4731779 | -58.72272239 | 141.4454448 | -76.44341637 | 176.8868327 | 36.23204548 | -48.46409096 | 75.59206824 | -127.1841365 |
| (7,0,4) | 3.669049552 | 18.6619009 | -57.17358169 | 140.3471634 | -57.10433585 | 140.2086717 | -75.70348939 | 177.4069788 | 37.30293294 | -48.60586589 | 77.43370693 | -128.8674139 |
| (7,0,5) | 2.754862929 | 22.49027414 | -57.39575914 | 142.7915183 | -56.51307901 | 141.026158 | -73.6622269 | 175.3244538 | 37.37941743 | -46.75883487 | 78.29893654 | -128.5978731 |
| (7,0,6) | 4.338524272 | 21.32295146 | -57.39574129 | 144.7914826 | -56.4850541 | 142.9701082 | -73.61485196 | 177.2297039 | 38.09704692 | -46.19409385 | 79.63584603 | -129.2716921 |
| (7,0,7) | 5.319976994 | 21.36004601 | -56.86671122 | 145.7334224 | -56.07374257 | 144.1474851 | -72.58161691 | 177.1632338 | 38.19024019 | -44.38048038 | 79.72625009 | -127.4525002 |
| (7,0,8) | 7.003048379 | 19.99390324 | -56.82629034 | 147.6525807 | -55.43732229 | 144.8746446 | -71.78008053 | 177.5601611 | 38.17830441 | -42.35660883 | 79.93602004 | -125.8720401 |
| (7,0,9) | 8.13837955 | 19.7232409 | -56.41912398 | 148.838248 | -55.17960112 | 146.3592022 | -71.78190392 | 179.5638078 | 38.32166814 | -40.64333628 | 75.5122357 | -115.0244714 |
| (7,0,10) | 4.74017134 | 28.51965732 | -56.03313933 | 150.0662787 | -55.13506228 | 148.2701246 | -73.2629273 | 184.5258546 | 38.35302448 | -38.70604896 | 78.83925849 | -119.678517 |
| (8,0,0) | -0.426206327 | 20.85241265 | -58.24376341 | 136.4875268 | -58.51090363 | 137.0218073 | -78.86916032 | 177.7383206 | 34.47135876 | -48.94271751 | 78.83925849 | -119.678517 |
| (8,0,1) | -0.493102898 | 22.9862058 | -58.03818478 | 138.0763696 | -57.06799101 | 136.135982 | -78.23272024 | 178.4654405 | 34.77045518 | -47.54091036 | 76.88390459 | -131.7678092 |
| (8,0,2) | 2.085377439 | 19.82924512 | -58.03816427 | 140.0763285 | -56.06301506 | 136.1260301 | -78.12293389 | 180.2458678 | 36.07466121 | -48.14932242 | 78.26205259 | -132.5241052 |
| (8,0,3) | 2.180285341 | 21.63942932 | -57.11607512 | 140.2321502 | -58.4893015 | 142.978603 | -76.73365533 | 179.4673107 | 36.36186822 | -46.72373643 | 78.81393587 | -131.6278717 |
| (8,0,4) | 3.708199201 | 20.5836016 | -56.99576797 | 141.9915359 | -57.09569479 | 142.1913896 | -74.77638125 | 177.5527625 | 37.39565041 | -46.79130081 | 79.12781024 | -130.2556205 |
| (8,0,5) | 2.794834694 | 24.41033061 | -56.82833134 | 143.6566627 | -57.09215975 | 144.1843195 | -74.14462653 | 178.2892531 | 37.41727611 | -44.83455223 | 79.26271312 | -128.5254262 |
| (8,0,6) | 4.836524013 | 22.32695197 | -56.74710436 | 145.4942087 | -55.87892306 | 143.7578461 | -72.58740869 | 177.1748174 | 36.77817125 | -41.55634249 | 80.28473758 | -128.5694752 |
| (8,0,7) | 6.012216634 | 21.97556673 | -56.72701684 | 147.4540337 | -55.97861231 | 145.9572246 | -71.84811478 | 177.6962296 | 36.93229434 | -39.86458867 | 79.43364255 | -124.8672851 |
| (8,0,8) | 7.520128565 | 20.95974287 | -56.03389702 | 148.067794 | -55.3429269 | 146.6858538 | -70.95710886 | 177.9142177 | 37.59047126 | -39.18094251 | 80.2102947 | -124.4205894 |
| (8,0,9) | 8.292677567 | 21.41464487 | -56.08702883 | 150.1740577 | -55.05262508 | 148.1052502 | -70.84884707 | 179.6976941 | 38.38098053 | -38.76196107 | 81.39690653 | -124.7938131 |
| (8,0,10) | 4.79199311 | 30.41601378 | -56.13796841 | 152.2759368 | -55.00932858 | 150.0186572 | -71.00283658 | 182.0056732 | 38.55515844 | -37.11031688 | 81.74855504 | -123.4971101 |

| **ARIMA** | **PRx** | | **HRV_HF** | | **HRV_LF** | | **HRV_VLF** | | **HRV_LF_HF** | | **HRV_HF_LF** | |
| --- | --- | --- | --- | --- | --- | --- | --- | --- | --- | --- | --- | --- |
| **Model** | **LL** | **AIC** | **LL** | **AIC** | **LL** | **AIC** | **LL** | **AIC** | **LL** | **AIC** | **LL** | **AIC** |
| (9,0,0) | 0.473972312 | 21.05205538 | -58.1886577 | 138.3773154 | -58.4100819 | 138.8201638 | -78.83715624 | 179.6743125 | 35.35789873 | -48.71579747 | 76.76783676 | -131.5356735 |
| (9,0,1) | 2.322998873 | 19.35400225 | -58.03817056 | 140.0763411 | -58.40601229 | 140.8120246 | -78.23253158 | 180.4650632 | 35.36730955 | -46.73461909 | 76.93526716 | -129.8705343 |
| (9,0,2) | 2.275188639 | 21.44962272 | -57.28522303 | 140.5704461 | -58.40639266 | 142.8127853 | -76.57523032 | 179.1504606 | 36.01722087 | -46.03444175 | 76.78266397 | -127.5653279 |
| (9,0,3) | 4.300595074 | 19.39880985 | -56.91527695 | 141.8305539 | -57.37950679 | 142.7590136 | -77.69912074 | 183.3982415 | 36.11557791 | -44.23115582 | 78.93790739 | -129.8758148 |
| (9,0,4) | 5.546895672 | 18.90620866 | -56.88067741 | 143.7613548 | -57.09514994 | 144.1902999 | -74.77394594 | 179.5478919 | 36.36518968 | -42.73037935 | 80.58090898 | -131.161818 |
| (9,0,5) | 5.719486343 | 20.56102731 | -56.75662433 | 145.5132487 | -56.95736583 | 145.9147317 | -74.14442401 | 180.288848 | 36.90042015 | -41.80084031 | 79.28585046 | -126.5717009 |
| (9,0,6) | 6.694981849 | 20.6100363 | -56.72984624 | 147.4596925 | -56.85018716 | 147.7003743 | -72.54276189 | 179.0855238 | 38.19317469 | -42.38634938 | 79.73497722 | -125.4699544 |
| (9,0,7) | 6.827410985 | 22.34517803 | -56.2280117 | 148.4560234 | -56.21454793 | 148.4290959 | -71.84809125 | 179.6961825 | 37.65216704 | -39.30433408 | 79.73497722 | -125.4699544 |
| (9,0,8) | 8.322092742 | 21.35581452 | -55.90095127 | 149.8019025 | -55.16999 | 148.33998 | -71.84569274 | 181.6913855 | 38.63449375 | -39.26898749 | 80.48312054 | -122.9662411 |
| (9,0,9) | 8.500230763 | 22.99953847 | -56.1040191 | 152.2080382 | -54.46570445 | 148.9314089 | -71.64154942 | 183.2830988 | 38.63449375 | -39.26898749 | 80.63126693 | -121.2625339 |
| (9,0,10) | 7.919902926 | 26.16019415 | -55.881838 | 153.763676 | -54.47733107 | 150.9546621 | -71.64154942 | 183.2830988 | 38.75824609 | -35.51649219 | 81.34333754 | -120.6866751 |
| (10,0,0) | 0.544234458 | 22.91153108 | -58.123858 | 140.247716 | -58.14600428 | 140.2920086 | -76.8693264 | 177.7386528 | 35.37764906 | -46.75529811 | 81.34333754 | -120.6866751 |
| (10,0,1) | 0.489985751 | 25.0200285 | -58.01698622 | 142.0339724 | -58.37591661 | 142.7518332 | -76.12054378 | 178.2410876 | 35.67245798 | -45.34491595 | 77.19420641 | -128.3884128 |
| (10,0,2) | 2.477044457 | 23.04591109 | -57.65386664 | 143.3077333 | -57.95372291 | 143.9074458 | -75.39102505 | 178.7820501 | 36.02115983 | -44.04231965 | 79.22206059 | -130.4441212 |
| (10,0,3) | 4.53503504 | 20.92992992 | -56.84412871 | 143.6882574 | -56.21443218 | 142.4288644 | -74.6902898 | 179.3805796 | 36.31338536 | -42.62677072 | 79.22713832 | -128.4542766 |
| (10,0,4) | 5.646708189 | 20.70658362 | -56.6173809 | 145.2347618 | -56.43626075 | 144.8725215 | -72.37967938 | 176.7593588 | 36.36862291 | -40.73724582 | 79.19673483 | -126.3934697 |
| (10,0,5) | 5.728729222 | 22.54254156 | -56.64217083 | 147.2843417 | -56.83016232 | 147.6603246 | -72.3793895 | 178.758779 | 37.79615586 | -41.59231171 | 79.22935619 | -124.4587124 |
| (10,0,6) | 6.733182628 | 22.53363474 | -56.43728522 | 148.8745704 | -55.85906803 | 147.7181361 | -71.43683634 | 178.8736727 | 38.39805519 | -40.79611037 | 80.50342312 | -125.0068462 |
| (10,0,7) | 7.636134591 | 22.72773082 | -56.57800824 | 151.1560165 | -54.43349124 | 146.8669825 | -71.32775546 | 180.6555109 | 38.21059259 | -38.42118518 | 81.94175945 | -125.8835189 |
| (10,0,8) | 8.40649851 | 23.18700298 | -56.1262927 | 152.2525854 | -54.7331626 | 149.4663252 | -71.22866564 | 182.4573313 | 38.62487088 | -37.24974177 | 80.52576244 | -121.0515249 |
| (10,0,9) | 8.830097236 | 24.33980553 | -56.10748394 | 154.2149679 | -54.46708878 | 150.9341776 | -71.22532996 | 184.4506599 | 38.83160136 | -35.66320272 | 81.96711266 | -121.9342253 |
| (10,0,10) | 8.830097236 | 24.33980553 | -55.96363456 | 155.9272691 | -54.45183626 | 152.9036725 | -70.10669982 | 184.2133996 | 38.87177131 | -33.74354261 | 80.24560695 | -116.4912139 |

| **ARIMA** | **HRV_RMS** | | **HRV_TOT** | | **BRS** | | **BPV_S** | | **BPV_D** | | **BPV_M** | |
| --- | --- | --- | --- | --- | --- | --- | --- | --- | --- | --- | --- | --- |
| **Model** | **LL** | **AIC** | **LL** | **AIC** | **LL** | **AIC** | **LL** | **AIC** | **LL** | **AIC** | **LL** | **AIC** |
| (1,0,0) | -46.53971979 | 99.07943957 | -137.5776057 | 281.1552115 | -173.6606334 | 353.3212668 | -104.6850673 | 215.3701345 | -78.54195401 | 163.083908 | -126.776549 | 259.553098 |
| (1,0,1) | -46.34891368 | 100.6978274 | -136.8681758 | 281.7363515 | -172.7178161 | 353.4356322 | -104.657078 | 217.3141559 | -76.75939867 | 161.5187973 | -126.7191888 | 261.4383777 |
| (1,0,2) | -46.14822937 | 102.2964587 | -136.8514398 | 283.7028796 | -172.4103094 | 354.8206188 | -104.5936133 | 219.1872265 | -75.17644076 | 160.3528815 | -123.9346952 | 257.8693904 |
| (1,0,3) | -45.64676286 | 103.2935257 | -136.8494463 | 285.6988926 | -172.3872636 | 356.7745272 | -104.5003698 | 221.0007396 | -75.17639054 | 162.3527811 | -123.8133649 | 259.6267299 |
| (1,0,4) | -45.64635929 | 105.2927186 | -136.4770537 | 286.9541073 | -172.3262503 | 358.6525005 | -102.4683863 | 218.9367726 | -73.54259901 | 161.085198 | -123.7686388 | 261.5372776 |
| (1,0,5) | -45.55835822 | 107.1167164 | -135.399305 | 286.7986099 | -172.1919092 | 360.3838184 | -102.4070209 | 220.8140418 | -73.28941804 | 162.5788361 | -124.4095001 | 264.8190002 |
| (1,0,6) | -45.36466759 | 108.7293352 | -133.7297797 | 285.4595595 | -171.8963786 | 361.7927572 | -102.2123329 | 222.4246658 | -72.72031395 | 163.4406279 | -123.6223006 | 265.2446012 |
| (1,0,7) | -44.83194596 | 109.6638919 | -133.643892 | 287.2877841 | -170.2755718 | 360.5511436 | -102.0983285 | 224.196657 | -72.65155233 | 165.3031047 | -123.9152884 | 267.8305769 |
| (1,0,8) | -44.95349148 | 111.906983 | -133.5932076 | 289.1864152 | -170.4215806 | 362.8431612 | -102.0346064 | 226.0692127 | -72.51160209 | 167.0232042 | -123.1807873 | 268.3615745 |
| (1,0,9) | -43.41467119 | 110.8293424 | -133.5756318 | 291.1512635 | -165.3618707 | 354.7237414 | -102.0215234 | 228.0430468 | -71.67879233 | 167.3575847 | -122.5637449 | 269.1274898 |
| (1,0,10) | -44.208054 | 114.416108 | -132.7247871 | 291.4495742 | -165.2608876 | 356.5217753 | -101.0625522 | 228.1251044 | -71.61822953 | 169.2364591 | -121.3390829 | 268.6781659 |
| (2,0,0) | -46.29815947 | 100.5963189 | -136.9917083 | 281.9834165 | -173.1181484 | 354.2362969 | -104.6521019 | 217.3042039 | -75.38772245 | 158.7754449 | -126.6690852 | 261.3381703 |
| (2,0,1) | -46.51514639 | 103.0302928 | -136.8519391 | 283.7038782 | -172.4390052 | 354.8780105 | -103.9249284 | 217.8498568 | -74.64538107 | 159.2907621 | -126.1884601 | 262.3769201 |
| (2,0,2) | -46.51514639 | 103.0302928 | -136.758165 | 285.51633 | -172.3972277 | 356.7944554 | -103.5611609 | 219.1223219 | -74.57047858 | 161.1409572 | -123.7733707 | 259.5467414 |
| (2,0,3) | -46.12073973 | 106.2414795 | -136.746381 | 287.492762 | -172.330707 | 358.6614139 | -103.7026085 | 221.405217 | -73.53042368 | 161.0608474 | -121.8547295 | 257.7094589 |
| (2,0,4) | -45.41754965 | 106.8350993 | -136.4349684 | 288.8699368 | -169.2147256 | 354.4294512 | -102.3679452 | 220.7358903 | -72.94435027 | 161.8887005 | -123.4591596 | 262.9183192 |
| (2,0,5) | -45.24104849 | 108.482097 | -136.4559774 | 290.9119547 | -172.3254276 | 362.6508553 | -102.067387 | 222.134774 | -72.93310715 | 163.8662143 | -123.7685781 | 265.5371562 |
| (2,0,6) | -44.64454711 | 109.2890942 | -133.7050227 | 287.4100454 | -170.6447672 | 361.2895344 | -102.1123657 | 224.2247314 | -72.72421358 | 165.4484272 | -122.6155637 | 265.2311274 |
| (2,0,7) | -44.60631564 | 111.2126313 | -133.6172578 | 289.2345156 | -169.9329758 | 361.8659516 | -102.0758553 | 226.1517106 | -72.6282276 | 167.2564552 | -122.4665078 | 266.9330156 |
| (2,0,8) | -44.42636056 | 112.8527211 | -133.432995 | 290.86599 | -169.8990665 | 363.7981329 | -96.83114818 | 217.6622964 | -72.57572985 | 169.1514597 | -122.4240837 | 268.8481673 |
| (2,0,9) | -43.53919694 | 113.0783939 | -133.2123928 | 292.4247855 | -165.187211 | 356.3744219 | -101.0769825 | 228.153965 | -70.76089981 | 167.5217996 | -120.7939379 | 267.5878758 |
| (2,0,10) | -42.74466132 | 113.4893226 | -132.754445 | 293.50889 | -164.5517719 | 357.1035438 | -100.5797693 | 229.1595387 | -70.57212941 | 169.1442588 | -121.3219314 | 270.6438629 |
| (3,0,0) | -45.9945629 | 101.9891258 | -136.8331086 | 283.6662173 | -172.5945173 | 355.1890346 | -104.5581438 | 219.1162876 | -74.48274099 | 158.965482 | -123.7584928 | 257.5169856 |
| (3,0,1) | -46.20345149 | 104.406903 | -136.6877864 | 285.3755727 | -172.2432407 | 356.4864814 | -104.5581438 | 219.1162876 | -73.9561158 | 159.9122316 | -123.649384 | 259.2987681 |
| (3,0,2) | -43.56089334 | 101.1217867 | -136.1113859 | 286.2227718 | -172.0795098 | 358.1590196 | -104.5581438 | 219.1162876 | -73.71389461 | 161.4277892 | -123.6376317 | 261.2752634 |
| (3,0,3) | -41.25435308 | 98.50870616 | -136.0561522 | 288.1123045 | -172.0301035 | 360.0602069 | -103.1344178 | 222.2688356 | -72.92214892 | 161.8442978 | -123.4603833 | 262.9207666 |
| (3,0,4) | -44.89518075 | 107.7903615 | -135.7390302 | 289.4780604 | -170.9344106 | 359.8688213 | -97.49795898 | 212.995918 | -72.92266588 | 163.8453318 | -121.5812027 | 261.1624054 |
| (3,0,5) | -44.88160134 | 109.7632027 | -133.6558577 | 287.3117155 | -170.4855285 | 360.971057 | -97.49795898 | 212.995918 | -71.79107329 | 163.5821466 | -119.8982591 | 259.7965183 |
| (3,0,6) | -44.61840419 | 111.2368084 | -133.6483979 | 289.2967958 | -169.5290674 | 361.0581347 | -101.0776071 | 224.1552142 | -71.42519991 | 164.8503998 | -122.885434 | 267.770868 |
| (3,0,7) | -44.46603469 | 112.9320694 | -133.4332087 | 290.8664174 | -168.9332665 | 361.8665329 | -101.0715784 | 226.1431568 | -72.01955041 | 168.0391008 | -122.649126 | 269.298252 |
| (3,0,8) | -43.54925745 | 113.0985149 | -133.2097257 | 292.4194513 | -168.5544837 | 363.1089675 | -101.0491351 | 228.0982701 | -70.80586134 | 167.6117227 | -122.4549969 | 270.9099939 |
| (3,0,9) | -43.25807111 | 114.5161422 | -131.7886661 | 291.5773321 | -165.1521859 | 358.3043718 | -101.023552 | 230.047104 | -70.6269868 | 169.2539736 | -116.8620547 | 261.7241094 |
| (3,0,10) | -42.11781307 | 114.2356261 | -131.7514238 | 293.5028477 | -165.1517085 | 360.3034169 | -101.0078552 | 232.0157105 | -70.57212934 | 171.1442587 | -120.9257208 | 271.8514417 |
| (4,0,0) | -45.2822233 | 102.5644466 | -136.7664174 | 285.5328348 | -172.3374855 | 356.6749711 | -104.5491815 | 221.0983631 | -74.45479245 | 160.9095849 | -123.6355434 | 259.2710868 |
| (4,0,1) | -42.93814316 | 99.87628632 | -136.6784651 | 287.3569302 | -172.0413223 | 358.0826446 | -103.5572117 | 221.1144235 | -73.7589965 | 161.517993 | -123.6123015 | 261.2246029 |
| (4,0,2) | -42.91654663 | 101.8330933 | -136.0822834 | 288.1645668 | -170.941534 | 357.8830679 | -103.1430397 | 222.2860793 | -72.79624562 | 161.5924912 | -123.5994862 | 263.1989724 |
| (4,0,3) | -41.78244141 | 101.5648828 | -136.078092 | 290.1561841 | -171.714157 | 361.4283141 | -102.5082563 | 223.0165127 | -71.7521058 | 161.5042116 | -123.4352754 | 264.8705508 |
| (4,0,4) | -41.93747185 | 103.8749437 | -135.7120512 | 291.4241023 | -171.233685 | 362.4673699 | -102.2113334 | 224.4226667 | -72.76606239 | 165.5321248 | -121.5554759 | 263.1109517 |
| (4,0,5) | -41.07358221 | 104.1471644 | -135.7089714 | 293.4179428 | -171.6159356 | 365.2318713 | -101.8197224 | 225.6394448 | -72.47141025 | 166.9428205 | -121.0834301 | 264.1668603 |
| (4,0,6) | -39.95974624 | 103.9194925 | -135.6774366 | 295.3548731 | -171.1510129 | 366.3020258 | -101.0704992 | 226.1409983 | -71.46719225 | 166.9343845 | -118.058667 | 260.1173339 |
| (4,0,7) | -44.41370072 | 114.8274014 | -133.3678482 | 292.7356965 | -168.1490923 | 362.2981847 | -95.41674065 | 216.8334813 | -70.54039882 | 167.0807976 | -118.1780354 | 262.3560709 |
| (4,0,8) | -42.09587902 | 112.191758 | -133.4065546 | 294.8131092 | -167.958937 | 363.917874 | -96.4719499 | 220.9438998 | -69.80607383 | 167.6121477 | -120.078334 | 268.156668 |
| (4,0,9) | -43.01481345 | 116.0296269 | -131.8368541 | 293.6737081 | -165.1502401 | 360.3004802 | -99.03172881 | 228.0634576 | -70.61343424 | 171.2268685 | -121.3800249 | 272.7600499 |
| (4,0,10) | -37.79537941 | 107.5907588 | -131.5124344 | 295.0248689 | -164.3101773 | 360.6203545 | -100.9956707 | 233.9913415 | -70.00650055 | 172.0130011 | -119.434719 | 270.869438 |

| **ARIMA** | **HRV_RMS** | | **HRV_TOT** | | **BRS** | | **BPV_S** | | **BPV_D** | | **BPV_M** | |
| --- | --- | --- | --- | --- | --- | --- | --- | --- | --- | --- | --- | --- |
| **Model** | **LL** | **AIC** | **LL** | **AIC** | **LL** | **AIC** | **LL** | **AIC** | **LL** | **AIC** | **LL** | **AIC** |
| (5,0,0) | -45.24217588 | 104.4843518 | -136.4437613 | 286.8875226 | -172.3371238 | 358.6742475 | -101.8297178 | 217.6594356 | -74.09976355 | 162.1995271 | -123.6160494 | 261.2320988 |
| (5,0,1) | -45.15311801 | 106.306236 | -136.3296642 | 288.6593283 | -171.9419468 | 359.8838936 | -99.04097805 | 214.0819561 | -73.74701466 | 163.4940293 | -123.6094821 | 263.2189643 |
| (5,0,2) | -42.89015483 | 103.7803097 | -134.5062904 | 287.0125809 | -170.9327575 | 359.865515 | -98.89047976 | 215.7809595 | -72.61542989 | 163.2308598 | -122.8286841 | 263.6573682 |
| (5,0,3) | -44.88232467 | 109.7646493 | -135.687238 | 291.374476 | -168.9106163 | 357.8212326 | -96.79427456 | 213.5885491 | -73.59466366 | 167.1893273 | -122.8231899 | 265.6463799 |
| (5,0,4) | -42.38677839 | 106.7735568 | -135.7119595 | 293.423919 | -169.5142464 | 361.0284928 | -101.8522748 | 225.7045496 | -72.54983627 | 167.0996725 | -120.4261813 | 262.8523626 |
| (5,0,5) | -40.24708227 | 104.4941645 | -135.7077758 | 295.4155517 | -170.1989486 | 364.3978972 | -101.8519871 | 227.7039743 | -72.46846279 | 168.9369256 | -120.406856 | 264.813712 |
| (5,0,6) | -39.57881814 | 105.1576363 | -133.0994355 | 292.1988711 | -167.7391843 | 361.4783686 | -101.0551598 | 228.1103197 | -72.47140528 | 170.9428106 | -118.6552498 | 263.3104996 |
| (5,0,7) | -43.74933088 | 115.4986618 | -133.2663264 | 294.5326529 | -167.6540751 | 363.3081501 | -99.7663417 | 227.5326834 | -71.11018253 | 170.2203651 | -118.2229325 | 264.4458651 |
| (5,0,8) | -41.13311552 | 112.266231 | -131.6576215 | 293.315243 | -168.0143604 | 366.0287208 | -96.11925181 | 222.2385036 | -70.76921441 | 171.5384288 | -118.3396899 | 266.6793798 |
| (5,0,9) | -38.78829465 | 109.5765893 | -129.1982783 | 290.3965566 | -165.1077246 | 362.2154493 | -97.31650826 | 226.6330165 | -70.34984405 | 172.6996881 | -115.4506831 | 262.9013662 |
| (5,0,10) | -37.76506538 | 109.5301308 | -130.8838448 | 295.7676895 | -164.1063307 | 362.2126615 | -94.25017076 | 222.5003415 | -68.33750931 | 170.6750186 | -119.2481512 | 272.4963025 |
| (6,0,0) | -44.98615429 | 105.9723086 | -136.1587619 | 288.3175237 | -172.2280857 | 360.4561713 | -101.6346408 | 219.2692816 | -73.66709118 | 163.3341824 | -123.5656838 | 263.1313676 |
| (6,0,1) | -45.01307879 | 108.0261576 | -136.1587608 | 290.3175217 | -170.3188454 | 358.6376908 | -98.96521519 | 215.9304304 | -73.6188074 | 165.2376148 | -123.2594638 | 264.5189276 |
| (6,0,2) | -42.52383177 | 105.0476635 | -134.489376 | 288.9787521 | -171.2363737 | 362.4727475 | -98.6416844 | 217.2833688 | -72.55556498 | 165.11113 | -122.6590067 | 265.3180135 |
| (6,0,3) | -42.80696358 | 107.6139272 | -134.4993987 | 290.9987973 | -171.0508418 | 364.1016835 | -98.89097419 | 219.7819484 | -72.59711796 | 167.1942359 | -122.4682487 | 266.9364974 |
| (6,0,4) | -40.22947414 | 104.4589483 | -134.474644 | 292.949288 | -170.1744272 | 364.3488543 | -96.72908218 | 217.4581644 | -71.41865056 | 166.8373011 | -120.4075689 | 264.8151378 |
| (6,0,5) | -42.17227133 | 110.3445427 | -133.6231791 | 293.2463581 | -168.9777906 | 363.9555813 | -95.44280122 | 216.8856024 | -71.4186315 | 168.837263 | -120.4089167 | 266.8178335 |
| (6,0,6) | -40.46951305 | 108.9390261 | -133.6081542 | 295.2163085 | -169.6005563 | 367.2011127 | -96.44085803 | 220.8817161 | -70.25756454 | 168.5151291 | -117.3261777 | 262.6523555 |
| (6,0,7) | -40.03846817 | 110.0769363 | -133.1947173 | 296.3894346 | -167.3376927 | 364.6753854 | -94.78732201 | 219.574644 | -69.10807485 | 168.2161497 | -117.7480032 | 265.4960064 |
| (6,0,8) | -39.01797159 | 110.0359432 | -133.1911417 | 298.3822834 | -167.6013196 | 367.2026393 | -95.11636225 | 222.2327245 | -70.16415004 | 172.3283001 | -116.2507344 | 264.5014688 |
| (6,0,9) | -39.52490779 | 113.0498156 | -132.2478738 | 298.4957477 | -165.0714372 | 364.1428745 | -93.64847345 | 221.2969469 | -67.9840382 | 169.9680764 | -120.200748 | 274.4014961 |
| (6,0,10) | -39.9877464 | 115.9754928 | -131.6324 | 299.2647999 | -164.0786678 | 364.1573356 | -98.63704414 | 233.2740883 | -67.9539655 | 171.907931 | -117.0081453 | 270.0162905 |
| (7,0,0) | -44.73779486 | 107.4755897 | -136.1587551 | 290.3175103 | -172.1318903 | 362.2637807 | -101.4154041 | 220.8308081 | -73.53634136 | 165.0726827 | -123.0227201 | 264.0454402 |
| (7,0,1) | -44.75631817 | 109.5126363 | -136.0639536 | 292.1279072 | -172.1571629 | 364.3143258 | -98.60941608 | 217.2188322 | -70.65189279 | 161.3037856 | -119.6175742 | 259.2351484 |
| (7,0,2) | -44.18746166 | 110.3749233 | -135.5138143 | 293.0276285 | -170.6109181 | 363.2218362 | -98.63879424 | 219.2775885 | -66.93748772 | 155.8749754 | -116.4911648 | 254.9823296 |
| (7,0,3) | -44.2041495 | 112.408299 | -135.5410728 | 295.0821455 | -168.5812465 | 361.1624929 | -98.30077169 | 220.6015434 | -72.48965435 | 168.9793087 | -120.2233867 | 264.4467734 |
| (7,0,4) | -41.56279165 | 109.1255833 | -133.8362952 | 293.6725905 | -170.1496031 | 366.2992062 | -95.54384441 | 217.0876888 | -71.41863109 | 168.8372622 | -120.2377043 | 266.4754085 |
| (7,0,5) | -41.64404652 | 111.288093 | -134.0904408 | 296.1808816 | -170.7571491 | 369.5142981 | -96.13789631 | 220.2757926 | -71.41863816 | 170.8372763 | -119.7021417 | 267.4042835 |
| (7,0,6) | -39.67104985 | 109.3420997 | -131.6488436 | 293.2976872 | -168.6211777 | 367.2423554 | -93.31490404 | 216.6298081 | -70.2575665 | 170.515133 | -119.703441 | 269.4068819 |
| (7,0,7) | -40.46951082 | 112.9390216 | -131.8178306 | 295.6356612 | -166.1494173 | 364.2988345 | -95.61943631 | 223.2388726 | -69.03674661 | 170.0734932 | -119.6599389 | 271.3198778 |
| (7,0,8) | -38.25031129 | 110.5006226 | -131.4177317 | 296.8354635 | -166.850736 | 367.7014721 | -91.87101853 | 217.7420371 | -69.03665059 | 172.0733012 | -116.525866 | 267.0517319 |
| (7,0,9) | -41.68000613 | 119.3600123 | -127.6165344 | 291.2330687 | -164.9967381 | 365.9934762 | -91.05395808 | 218.1079162 | -67.86973108 | 171.7394622 | -119.7782293 | 275.5564585 |
| (7,0,10) | -41.45261149 | 120.905223 | -130.7676285 | 299.5352569 | -163.8165977 | 365.6331953 | -93.30957143 | 224.6191429 | -67.61672914 | 173.2334583 | -119.1525717 | 276.3051433 |
| (8,0,0) | -44.45981879 | 108.9196376 | -135.0687885 | 290.1375771 | -171.7844378 | 363.5688756 | -101.2616092 | 222.5232183 | -73.26719775 | 166.5343955 | -122.7331983 | 265.4663966 |
| (8,0,1) | -44.47290292 | 110.9458058 | -131.6220233 | 285.2440465 | -168.8222056 | 359.6444113 | -98.198549 | 218.397098 | -70.02138742 | 162.0427748 | -119.3250937 | 260.6501874 |
| (8,0,2) | -43.62242862 | 111.2448572 | -129.0634628 | 282.1269256 | -170.8876571 | 365.7753142 | -100.9523799 | 225.9047599 | -66.90006799 | 157.800136 | -122.4089644 | 268.8179288 |
| (8,0,3) | -42.01725069 | 110.0345014 | -129.2318618 | 284.4637235 | -170.4942028 | 366.9884056 | -98.59577523 | 223.1915505 | -70.59615026 | 167.1923005 | -119.6379689 | 265.2759378 |
| (8,0,4) | -39.99614194 | 107.9922839 | -134.0950082 | 296.1900164 | -169.3374334 | 366.6748668 | -95.52816036 | 219.0563207 | -71.41860404 | 170.8372081 | -120.1594797 | 268.3189595 |
| (8,0,5) | -41.43737341 | 112.8747468 | -134.0961977 | 298.1923953 | -169.270955 | 368.5419101 | -97.53621077 | 225.0724215 | -71.41860737 | 172.8372147 | -119.7008425 | 269.4016851 |
| (8,0,6) | -39.9127358 | 111.8254716 | -127.1354802 | 286.2709605 | -166.9310449 | 365.8620898 | -94.73358203 | 221.4671641 | -70.33576727 | 172.6715345 | -119.6480086 | 271.2960172 |
| (8,0,7) | -39.54385467 | 113.0877093 | -126.9323127 | 287.8646255 | -167.0819699 | 368.1639398 | -95.26955658 | 224.5391132 | -69.03655782 | 172.0731156 | -119.0432579 | 272.0865157 |
| (8,0,8) | -37.74812671 | 111.4962534 | -131.142412 | 298.2848241 | -166.3371969 | 368.6743938 | -95.26955658 | 224.5391132 | -70.09893766 | 176.1978753 | -115.5152968 | 267.0305936 |
| (8,0,9) | -40.91787879 | 119.8357576 | -127.6036741 | 293.2073482 | -164.5280302 | 367.0560603 | -94.27314119 | 226.5462824 | -69.87530388 | 177.7506078 | -116.6127898 | 271.2255796 |
| (8,0,10) | -35.38706291 | 110.7741258 | -127.135509 | 294.271018 | -163.7384272 | 367.4768544 | -93.57179658 | 227.1435932 | -70.86218874 | 181.7243775 | -119.0874984 | 278.1749967 |

| **ARIMA** | **HRV_RMS** | | **HRV_TOT** | | **BRS** | | **BPV_S** | | **BPV_D** | | **BPV_M** | |
| --- | --- | --- | --- | --- | --- | --- | --- | --- | --- | --- | --- | --- |
| **Model** | **LL** | **AIC** | **LL** | **AIC** | **LL** | **AIC** | **LL** | **AIC** | **LL** | **AIC** | **LL** | **AIC** |
| (9,0,0) | -44.2954287 | 110.5908574 | -134.6460027 | 291.2920054 | -168.5748385 | 359.149677 | -101.0485766 | 224.0971532 | -72.89689895 | 167.7937979 | -122.4809677 | 266.9619354 |
| (9,0,1) | -44.14610968 | 112.2922194 | -134.6657363 | 293.3314727 | -168.0230105 | 360.0460209 | -97.45218795 | 218.9043759 | -72.91092346 | 169.8218469 | -122.0592812 | 268.1185624 |
| (9,0,2) | -43.1120585 | 112.224117 | -131.5127282 | 289.0254564 | -167.2121123 | 360.4242246 | -97.68991863 | 221.3798373 | -72.47427934 | 170.9485587 | -121.9738973 | 269.9477945 |
| (9,0,3) | -41.68984179 | 111.3796836 | -130.4206427 | 288.8412853 | -166.9570207 | 361.9140413 | -97.84467296 | 223.6893459 | -69.5670619 | 167.1341238 | -120.8135752 | 269.6271505 |
| (9,0,4) | -38.63012297 | 107.2602459 | -128.2890278 | 286.5780557 | -168.8571593 | 367.7143186 | -93.97760767 | 217.9552153 | -71.39078585 | 172.7815717 | -115.5373755 | 261.0747509 |
| (9,0,5) | -40.78446605 | 113.5689321 | -127.6120572 | 287.2241144 | -167.5239684 | 367.0479368 | -96.93692898 | 225.873858 | -69.47148986 | 170.9429797 | -119.0408091 | 270.0816181 |
| (9,0,6) | -40.07950446 | 114.1590089 | -126.3166437 | 286.6332875 | -165.2605792 | 364.5211583 | -94.53245481 | 223.0649096 | -69.16936765 | 172.3387353 | -115.8087657 | 265.6175313 |
| (9,0,7) | -39.52700551 | 115.054011 | -127.9249758 | 291.8499516 | -165.2269147 | 366.4538294 | -98.09703115 | 232.1940623 | -69.03566292 | 174.0713258 | -116.6663328 | 269.3326655 |
| (9,0,8) | -37.78304662 | 113.5660932 | -124.3529908 | 286.7059815 | -164.3501516 | 366.7003033 | -93.82004593 | 225.6400919 | -68.94921109 | 175.8984222 | -116.7977491 | 271.5954981 |
| (9,0,9) | -39.48315474 | 118.9663095 | -125.0980544 | 290.1961088 | -164.0743136 | 368.1486272 | -94.1238038 | 228.2476076 | -68.20594472 | 176.4118894 | -119.2121993 | 278.4243987 |
| (9,0,10) | -36.89320207 | 115.7864041 | -128.8837681 | 299.7675362 | -161.781374 | 365.562748 | -94.04305656 | 230.0861131 | -64.36153079 | 170.7230616 | -115.3989044 | 272.7978087 |
| (10,0,0) | -44.09074119 | 112.1814824 | -134.5077803 | 293.0155607 | -167.3163985 | 358.632797 | -99.98234141 | 223.9646828 | -71.98094561 | 167.9618912 | -121.7073805 | 267.414761 |
| (10,0,1) | -44.09107005 | 114.1821401 | -131.1667019 | 288.3334037 | -165.4273839 | 356.8547677 | -96.48333896 | 218.9666779 | -68.54361853 | 163.0872371 | -117.7360039 | 261.4720077 |
| (10,0,2) | -42.5859344 | 113.1718688 | -131.52169 | 291.0433799 | -165.2784703 | 358.5569405 | -100.2847915 | 228.5695831 | -71.3926342 | 170.7852684 | -120.4528676 | 268.9057351 |
| (10,0,3) | -41.61158397 | 113.2231679 | -131.4586337 | 292.9172674 | -165.2448903 | 360.4897805 | -96.92510036 | 223.8502007 | -68.07624706 | 166.1524941 | -118.1845839 | 266.3691678 |
| (10,0,4) | -41.87255189 | 115.7451038 | -128.4414226 | 288.8828452 | -164.4618772 | 360.9237545 | -98.82197954 | 229.6439591 | -65.4702674 | 162.9405348 | -119.7476482 | 271.4952964 |
| (10,0,5) | -41.10579134 | 116.2115827 | -127.7854024 | 289.5708049 | -164.401278 | 362.8025559 | -95.77327975 | 225.5465595 | -66.34475393 | 166.6895079 | -117.5549072 | 269.1098144 |
| (10,0,6) | -40.07452413 | 116.1490483 | -128.3416908 | 292.6833817 | -162.6002116 | 361.2004231 | -93.91217916 | 223.8243583 | -65.16019926 | 166.3203985 | -119.0173337 | 274.0346674 |
| (10,0,7) | -37.76987539 | 113.5397508 | -126.7062034 | 291.4124068 | -162.5807833 | 363.1615667 | -95.91447362 | 229.8289472 | -67.78540648 | 173.570813 | -114.7578857 | 267.5157713 |
| (10,0,8) | -37.7794901 | 115.5589802 | -126.7501764 | 293.5003528 | -163.0574407 | 366.1148815 | -94.00358801 | 228.007176 | -64.98258692 | 169.9651738 | -115.9364418 | 271.8728836 |
| (10,0,9) | -38.23767845 | 118.4753569 | -128.7331908 | 299.4663815 | -161.6490364 | 365.2980729 | -94.24263696 | 230.4852739 | -65.30194727 | 172.6038945 | -118.9713066 | 279.9426132 |
| (10,0,10) | -38.10413809 | 120.2082762 | -128.4213546 | 300.8427091 | -161.1529572 | 366.3059144 | -92.41055463 | 228.8211093 | -67.57373205 | 179.1474641 | -111.1907612 | 266.3815224 |

| **ARIMA** | **SBPV_LF** | | **SBPV_HF** | | **SBPV_TOT** | |
| --- | --- | --- | --- | --- | --- | --- |
| **Model** | **LL** | **AIC** | **LL** | **AIC** | **LL** | **AIC** |
| (1,0,0) | -115.7903318 | 237.5806636 | -68.79971317 | 143.5994263 | -139.2005101 | 284.4010202 |
| (1,0,1) | -113.5587048 | 235.1174097 | -67.13804351 | 142.276087 | -137.5486792 | 283.0973584 |
| (1,0,2) | -110.9266024 | 231.8532049 | -65.11866208 | 140.2373242 | -135.3094059 | 280.6188117 |
| (1,0,3) | -110.8965246 | 233.7930491 | -65.10435529 | 142.2087106 | -135.289209 | 282.5784181 |
| (1,0,4) | -110.7352984 | 235.4705968 | -64.91330329 | 143.8266066 | -135.1195075 | 284.239015 |
| (1,0,5) | -110.7052701 | 237.4105402 | -64.88664572 | 145.7732914 | -135.0882592 | 286.1765185 |
| (1,0,6) | -110.7034632 | 239.4069263 | -64.88610116 | 147.7722023 | -135.0882647 | 288.1765295 |
| (1,0,7) | -110.6421064 | 241.2842127 | -64.8394388 | 149.6788776 | -134.6464072 | 289.2928145 |
| (1,0,8) | -110.2829885 | 242.565977 | -64.366251 | 150.732502 | -134.5781749 | 291.1563498 |
| (1,0,9) | -109.1772622 | 242.3545244 | -64.22973542 | 152.4594708 | -134.4460506 | 292.8921012 |
| (1,0,10) | -108.1216093 | 242.2432185 | -62.30757724 | 150.6151545 | -132.1777266 | 290.3554532 |
| (2,0,0) | -112.0027643 | 232.0055286 | -65.99648515 | 139.9929703 | -136.3847742 | 280.7695484 |
| (2,0,1) | -111.9495455 | 233.8990911 | -65.84228051 | 141.684561 | -136.2175696 | 282.4351391 |
| (2,0,2) | -110.8593338 | 233.7186676 | -65.06822866 | 142.1364573 | -135.2510337 | 282.5020674 |
| (2,0,3) | -110.8707422 | 235.7414843 | -65.02304531 | 144.0460906 | -135.2372976 | 284.4745952 |
| (2,0,4) | -110.8037798 | 237.6075596 | -65.08467621 | 146.1693524 | -129.6326726 | 275.2653451 |
| (2,0,5) | -110.7004089 | 239.4008179 | -64.87762129 | 147.7552426 | -135.0752996 | 288.1505991 |
| (2,0,6) | -110.699207 | 241.398414 | -64.87744776 | 149.7548955 | -135.075289 | 290.1505781 |
| (2,0,7) | -103.0306066 | 228.0612132 | -57.75627523 | 137.5125505 | -135.0402285 | 292.080457 |
| (2,0,8) | -109.8179821 | 243.6359643 | -64.20944734 | 152.4188947 | -134.2723755 | 292.5447509 |
| (2,0,9) | -109.7208297 | 245.4416594 | -63.32884842 | 152.6576968 | -133.6283592 | 293.2567184 |
| (2,0,10) | -108.2675226 | 244.5350452 | -63.25048647 | 154.5009729 | -133.5808963 | 295.1617925 |
| (3,0,0) | -111.8933206 | 233.7866412 | -65.68277734 | 141.3655547 | -136.0250184 | 282.0500368 |
| (3,0,1) | -111.9795059 | 235.9590118 | -62.31210429 | 136.6242086 | -132.5754822 | 277.1509644 |
| (3,0,2) | -105.3833293 | 224.7666586 | -59.6563637 | 133.3127274 | -130.0467962 | 274.0935923 |
| (3,0,3) | -105.2264128 | 226.4528257 | -64.90229539 | 145.8045908 | -129.8543908 | 275.7087815 |
| (3,0,4) | -105.0994902 | 228.1989805 | -59.23736544 | 136.4747309 | -129.9419371 | 277.8838741 |
| (3,0,5) | -105.1234603 | 230.2469205 | -59.2605626 | 138.5211252 | -135.075413 | 290.150826 |
| (3,0,6) | -110.2222353 | 242.4444705 | -64.86369229 | 151.7273846 | -134.9974307 | 291.9948613 |
| (3,0,7) | -103.1343811 | 230.2687621 | -61.96479312 | 147.9295862 | -132.1704456 | 288.3408911 |
| (3,0,8) | -108.3651033 | 242.7302066 | -57.72398734 | 141.4479747 | -132.1665453 | 290.3330905 |
| (3,0,9) | -109.2318065 | 246.463613 | -63.05912605 | 154.1182521 | -133.2705864 | 294.5411729 |
| (3,0,10) | -107.2724563 | 244.5449126 | -61.17015019 | 152.3403004 | -131.4694919 | 292.9389839 |
| (4,0,0) | -110.8289885 | 233.657977 | -64.89629647 | 141.7925929 | -135.0500452 | 282.1000905 |
| (4,0,1) | -107.6206557 | 229.2413115 | -61.85263012 | 137.7052602 | -132.1333759 | 278.2667518 |
| (4,0,2) | -105.1902975 | 226.380595 | -61.91000247 | 139.8200049 | -129.7636718 | 275.5273436 |
| (4,0,3) | -105.3089838 | 228.6179675 | -59.52976161 | 137.0595232 | -129.9175555 | 277.8351111 |
| (4,0,4) | -105.2109093 | 230.4218186 | -59.43027488 | 138.8605498 | -129.4307336 | 278.8614672 |
| (4,0,5) | -105.5878075 | 233.175615 | -58.90310774 | 139.8062155 | -129.6185473 | 281.2370946 |
| (4,0,6) | -104.8939744 | 233.7879488 | -58.27378934 | 140.5475787 | -128.4929085 | 280.985817 |
| (4,0,7) | -107.6958071 | 241.3916141 | -61.96472933 | 149.9294587 | -132.1615446 | 290.3230891 |
| (4,0,8) | -107.6950207 | 243.3900414 | -61.96437652 | 151.928753 | -132.1631312 | 292.3262623 |
| (4,0,9) | -107.5631505 | 245.1263009 | -57.24846203 | 144.4969241 | -132.1631312 | 292.3262623 |
| (4,0,10) | -102.4635927 | 236.9271854 | -55.06443874 | 142.1288775 | -124.6444549 | 281.2889098 |

| **ARIMA** | **SBPV_LF** | | **SBPV_HF** | | **SBPV_TOT** | |
| --- | --- | --- | --- | --- | --- | --- |
| **Model** | **LL** | **AIC** | **LL** | **AIC** | **LL** | **AIC** |
| (5,0,0) | -110.4390566 | 234.8781132 | -64.66301227 | 143.3260245 | -134.8211573 | 283.6423145 |
| (5,0,1) | -107.5666727 | 231.1333454 | -61.77137228 | 139.5427446 | -132.1041718 | 280.2083435 |
| (5,0,2) | -107.6203425 | 233.2406849 | -61.78193697 | 141.5638739 | -132.0790474 | 282.1580948 |
| (5,0,3) | -107.8423538 | 235.6847076 | -61.80660121 | 143.6132024 | -131.551795 | 283.10359 |
| (5,0,4) | -105.0968516 | 232.1937032 | -58.98160636 | 139.9632127 | -129.4460618 | 280.8921235 |
| (5,0,5) | -104.8275261 | 233.6550523 | -59.09131118 | 142.1826224 | -134.3459237 | 292.6918474 |
| (5,0,6) | -104.3355985 | 234.671197 | -58.40619881 | 142.8123976 | -128.8816795 | 283.7633589 |
| (5,0,7) | -104.7171089 | 237.4342178 | -57.97965901 | 143.959318 | -128.8816795 | 283.7633589 |
| (5,0,8) | -104.776574 | 239.5531479 | -63.42917511 | 156.8583502 | -133.5633611 | 297.1267222 |
| (5,0,9) | -100.4973753 | 232.9947506 | -54.67225773 | 141.3445155 | -125.2474954 | 282.4949907 |
| (5,0,10) | -100.3873178 | 234.7746356 | -54.84617252 | 143.692345 | -124.920254 | 283.8405081 |
| (6,0,0) | -110.1200058 | 236.2400116 | -64.36574828 | 144.7314966 | -134.5644543 | 285.1289086 |
| (6,0,1) | -107.3238537 | 232.6477074 | -61.37789593 | 140.7557919 | -131.7699515 | 281.5399029 |
| (6,0,2) | -107.5406383 | 235.0812766 | -58.43897699 | 136.877954 | -131.7699515 | 281.5399029 |
| (6,0,3) | -106.9916229 | 235.9832458 | -60.89599496 | 143.7919899 | -131.7699515 | 281.5399029 |
| (6,0,4) | -106.9066336 | 237.8132672 | -61.23736913 | 146.4747383 | -131.4644588 | 286.9289176 |
| (6,0,5) | -106.9066336 | 237.8132672 | -59.40048446 | 144.8009689 | -129.6603363 | 285.3206727 |
| (6,0,6) | -103.9135402 | 235.8270803 | -57.99515888 | 143.9903178 | -129.2527142 | 286.5054283 |
| (6,0,7) | -103.9554663 | 237.9109326 | -56.99580871 | 143.9916174 | -128.7680421 | 287.5360843 |
| (6,0,8) | -104.0403415 | 240.0806831 | -57.86329384 | 147.7265877 | -128.2004538 | 288.4009077 |
| (6,0,9) | -99.49215317 | 232.9843063 | -54.14819159 | 142.2963832 | -123.7441156 | 281.4882313 |
| (6,0,10) | -99.76717607 | 235.5343521 | -54.09986046 | 144.1997209 | -123.6863665 | 283.3727329 |
| (7,0,0) | -110.0263628 | 238.0527255 | -64.26155161 | 146.5231032 | -134.4879734 | 286.9759467 |
| (7,0,1) | -107.0159569 | 234.0319138 | -61.00647927 | 142.0129585 | -131.3937782 | 282.7875563 |
| (7,0,2) | -109.466099 | 240.9321979 | -63.62648797 | 149.2529759 | -133.7615356 | 289.5230712 |
| (7,0,3) | -106.7679529 | 237.5359057 | -60.86234234 | 145.7246847 | -131.1668091 | 286.3336182 |
| (7,0,4) | -106.6844669 | 239.3689338 | -60.77096757 | 147.5419351 | -131.0578401 | 288.1156802 |
| (7,0,5) | -106.3091011 | 240.6182022 | -59.33138135 | 146.6627627 | -129.3635437 | 286.7270874 |
| (7,0,6) | -103.9701101 | 237.9402202 | -58.04195505 | 146.0839101 | -129.5052303 | 289.0104605 |
| (7,0,7) | -103.8942754 | 239.7885508 | -55.89985015 | 143.7997003 | -126.1762424 | 284.3524848 |
| (7,0,8) | -103.4918265 | 240.983653 | -57.06863603 | 148.1372721 | -127.6096157 | 289.2192314 |
| (7,0,9) | -99.33915174 | 234.6783035 | -54.1850781 | 144.3701562 | -123.7946622 | 283.5893243 |
| (7,0,10) | -99.31422999 | 236.62846 | -54.35761004 | 146.7152201 | -123.5339927 | 285.0679855 |
| (8,0,0) | -109.7120883 | 239.4241766 | -63.85866709 | 147.7173342 | -134.1108004 | 288.2216008 |
| (8,0,1) | -106.0076687 | 234.0153374 | -60.01812625 | 142.0362525 | -130.2197144 | 282.4394288 |
| (8,0,2) | -101.6246006 | 227.2492012 | -62.87478422 | 149.7495684 | -133.1356258 | 290.2712516 |
| (8,0,3) | -105.7169314 | 237.4338628 | -59.60401316 | 145.2080263 | -130.02882 | 286.0576401 |
| (8,0,4) | -102.0811766 | 232.1623533 | -56.84245925 | 141.6849185 | -126.1371454 | 280.2742908 |
| (8,0,5) | -106.5880139 | 243.1760278 | -60.66455004 | 151.3291001 | -130.9603215 | 291.9206429 |
| (8,0,6) | -106.4158554 | 244.8317108 | -61.55864908 | 155.1172982 | -128.8496154 | 289.6992308 |
| (8,0,7) | -102.4872235 | 238.9744469 | -57.22752973 | 148.4550595 | -127.8246997 | 289.6493995 |
| (8,0,8) | -103.5219782 | 243.0439563 | -55.80381198 | 147.607624 | -125.7851297 | 287.5702594 |
| (8,0,9) | -101.3326523 | 240.6653046 | -55.36237323 | 148.7247465 | -129.3449975 | 296.6899949 |
| (8,0,10) | -100.5568166 | 241.1136333 | -55.08134867 | 150.1626973 | -125.5604113 | 291.1208227 |

| **ARIMA** | **SBPV_LF** | | **SBPV_HF** | | **SBPV_TOT** | |
| --- | --- | --- | --- | --- | --- | --- |
| **Model** | **LL** | **AIC** | **LL** | **AIC** | **LL** | **AIC** |
| (9,0,0) | -109.1055181 | 240.2110362 | -63.21762944 | 148.4352589 | -133.419569 | 288.8391381 |
| (9,0,1) | -104.3750056 | 232.7500112 | -58.96699012 | 141.9339802 | -128.5009942 | 281.0019884 |
| (9,0,2) | -108.051397 | 242.1027939 | -62.17924148 | 150.358483 | -132.4454512 | 290.8909025 |
| (9,0,3) | -104.6377728 | 237.2755456 | -58.68011033 | 145.3602207 | -128.898812 | 285.7976241 |
| (9,0,4) | -105.3283583 | 240.6567166 | -59.12899846 | 148.2579969 | -129.4812167 | 288.9624333 |
| (9,0,5) | -101.5992502 | 235.1985003 | -56.46604409 | 144.9320882 | -125.5717077 | 283.1434154 |
| (9,0,6) | -106.1241181 | 246.2482363 | -57.58279461 | 149.1655892 | -128.0823757 | 290.1647515 |
| (9,0,7) | -106.0819505 | 248.163901 | -59.85611552 | 155.712231 | -125.7024304 | 287.4048608 |
| (9,0,8) | -101.0766211 | 240.1532422 | -54.89958592 | 147.7991718 | -125.6855507 | 289.3711014 |
| (9,0,9) | -103.5216325 | 247.043265 | -55.43468043 | 150.8693609 | -128.2778259 | 296.5556518 |
| (9,0,10) | -99.37422475 | 240.7484495 | -54.10122944 | 150.2024589 | -124.6959697 | 291.3919394 |
| (10,0,0) | -108.0363561 | 240.0727123 | -62.27451845 | 148.5490369 | -132.3095903 | 288.6191806 |
| (10,0,1) | -103.0760949 | 232.1521899 | -58.23802653 | 142.4760531 | -127.2250778 | 280.4501555 |
| (10,0,2) | -107.5259329 | 243.0518659 | -61.48804642 | 150.9760928 | -131.6914398 | 291.3828796 |
| (10,0,3) | -107.4827922 | 244.9655845 | -61.43908576 | 152.8781715 | -131.6807694 | 293.3615388 |
| (10,0,4) | -100.1068191 | 232.2136383 | -56.44772842 | 144.8954568 | -124.0945553 | 280.1891107 |
| (10,0,5) | -103.2707525 | 240.541505 | -56.91478948 | 147.829579 | -125.1636344 | 284.3272688 |
| (10,0,6) | -105.8637897 | 247.7275794 | -56.50984393 | 149.0196879 | -125.3689942 | 286.7379885 |
| (10,0,7) | -105.2034305 | 248.4068611 | -54.85283676 | 147.7056735 | -124.2199504 | 286.4399009 |
| (10,0,8) | -98.49319342 | 236.9863868 | -53.41797699 | 146.835954 | -123.8943861 | 287.7887722 |
| (10,0,9) | -102.4042781 | 246.8085563 | -56.67947436 | 155.3589487 | -127.5175434 | 297.0350868 |
| (10,0,10) | -103.4423195 | 250.8846389 | -57.55790491 | 159.1158098 | -125.987993 | 295.975986 |

*Optimal ARIMA models are determined by lowest AIC and highest LL (highlighted in gray). ARIMA model (autoregressive order, integrative order, moving average order). The Optimal ARIMA model demonstrates the model that is best fitted to the original data.*

*AIC, Akaike information criteria; ARIMA, autoregressive integrated moving average. BPV_D, standard deviation of diastolic blood pressure variability; BPV_M, standard deviation of mean blood pressure variability; BPV_S, standard deviation of systolic blood pressure variability; HRF_HF, heart rate variability high frequency; HRV_HF_LF, heart rate variability ratio between high/low frequency; HRV_LF, heart rate variability low frequency; HRV_LF_HF, heart rate variability ratio between low/high frequency; HRV_RMS, heart rate variability root mean square; HRV_TOT, heart rate variability total; HRV_VLF, heart rate variability very low frequency; LL, log likelihood; PRx, pressure reactivity; SBPV_HF, spectral blood pressure variability high frequency; SBPV_LF, spectral blood pressure variability low frequency; SBPV_TOT, spectral blood pressure variability total;*
