# Supplementary material for: Dynamic Temporal Relationship Between Autonomic Function and Cerebrovascular Reactivity in Moderate/Severe Traumatic Brain Injury
Source: Front Netw Physiol. 2022 Feb 16;2:837860. doi: 10.3389/fnetp.2022.837860 (PMC10013014; doi:10.3389/fnetp.2022.837860)
Supplement: Supplementary file 7 [file DataSheet8.DOCX]

**Appendix G. Summary of all indexes and corresponding abbreviations**

*G1. Variables*

ARV (autonomic response variables) – entails all the surrogate variables used in this study to determine autonomic functionality

BRS (calculated using a modification of the sequential cross-correlation method) - provides a useful synthetic index of neural regulation at the sinus atrial node

BPV_D (diastolic blood pressure) - unknown association

BPV_M (mean blood pressure) - unknown association

BPV_S (systolic blood pressure) - unknown association

HRV (heart rate variability) - measures the variability in heart rate over a time window

HRV_HF (0.15 - 0.4 Hz) - reflects parasympathetic (vagal) activity

HRV_LF (0.04 - 0.15 Hz) - sympathetic modulation or parameter that includes both sympathetic and vagal influences

HRV_LF_HF (HRV_LF divided by HRV_HF) - minor sympathetic vagal balance or sympathetic modulations

HRV_RMS (root mean square differences between consecutive RR intervals of a heartbeat waveform) - estimates the vagally mediated changes in autonomics

HRV_TOT (<0.15Hz) - a non-specific variable that reflects the overall autonomic activity

HRV_VLF (<0.04 Hz) - reflects slow mechanisms of sympathetic activity

ICP (mean intracranial pressure)

MAP (mean arterial pressure)

PRx (correlation between intracranial pressure and mean blood pressure) - surrogate measure of cerebrovascular reactivity

SBPV _LF (0.077 - 0.15 Hz) - variability is modulated by sympathetic modulation of vascular tone and endothelial-derived

SBPV _HF (0.15 - 0.4 Hz) - influenced by changes in cardiac output

SBPV _TOT (total power over the full frequency range) - unknown association

*G2. Statistical*

ACF (autocorrelation function)

ADF (augmented Dickey Fuller)

AIC (Akaike Information Criterion)

ARIMA (autoregressive integrative moving average)

ARMA (autoregressive moving average)

IRF (impulse response function)

KPSS (Kwiatkowski–Phillips–Schmidt–Shin)

LL (Log-Likelihood)

PACF (partial autocorrelation function)

VARIMA (vector autoregressive integrative moving average)

VARMA (vector autoregressive moving average model)
