# Supplementary material for: Dynamic Temporal Relationship Between Autonomic Function and Cerebrovascular Reactivity in Moderate/Severe Traumatic Brain Injury
Source: Front Netw Physiol. 2022 Feb 16;2:837860. doi: 10.3389/fnetp.2022.837860 (PMC10013014; doi:10.3389/fnetp.2022.837860)
Supplement: Supplementary file 8 [file DataSheet1.DOCX]

Appendix A.1. General Box-Jenkin’s ARMA model for PRx

1. ${PRx}_{t}= c + \varepsilon_{t} + \sum_{i=1}^{p} \varphi_{i}{PRx}_{t-i} + \sum_{j=1}^{q} \theta_{j}\varepsilon_{t-j}$

*ARMA= autoregressive moving average, c = constant, i = integer, j = integer, p = autoregressive order, PRx = pressure reactivity index, q = moving average order, t = time “t”, θ = moving average coefficient at time “t–j”, φ = autoregressive coefficient at time “t–i”, Ɛ = error term*

Appendix A.2. General Box-Jenkin’s VARMA model for PRx and an ARV

(2) $[\begin{matrix} {PRx}_{t} \\ {ARV}_{t} \end{matrix}] = c +\boldsymbol{E}_{t} +\sum_{i=1}^{p} \boldsymbol{\varphi}_{i}[\begin{matrix} {PRx}_{t-i} \\ {ARV}_{t-i} \end{matrix}] + \sum_{j=1}^{q} \boldsymbol{\theta}_{j}\boldsymbol{E}_{t-j}$

*VARMA = vector autoregressive moving average, ARV = an autonomic response variable, c = constant,* ***E*** *= error terms matrices of 2 elements, i = integer, j = integer, p = VARMA autoregressive order, PRx = pressure reactivity index, q = VARMA moving average order, t = time “t”,* ***θ****= moving average 2x2 matrices at time “t–j”,* ***φ*** *= autoregressive 2x2 matrices at time “t–i”*
